# Supplementary material for: A biofeedback-enhanced therapeutic exercise video game intervention for young people with cerebral palsy: A randomized single-case experimental design feasibility study
Source: PLoS One. 2020 Jun 22;15(6):e0234767. doi: 10.1371/journal.pone.0234767 (PMC7307764; doi:10.1371/journal.pone.0234767)
Supplement: S1 File — (PDF) [file pone.0234767.s001.pdf]

S1 Biofeedback technology development

Integrating biofeedback into an adapted commercial game for therapeutic exercise was achieved through an iterative participatory design process.

Co-creation process

Nine individuals with CP and three occupational therapists were active members in the development of the goals of the ICP and in the implementation of the control and feedback mechanisms used in the game. Our participatory design process involves conducting interviews to understand priorities and iterative design sessions to improve the use of and feedback in the game. Initial biofeedback elements to include were derived from a systematic review [1].

After establishing the participants' and therapists' priorities, we began building and testing the game through design sessions. During each design session, the participants wore the muscle activity sensor (Myo Armband) and played the game. The participant was encouraged to verbalize their thoughts related to biofeedback timing, aesthetic design, comprehension, and motivation during and after play-testing. After each design session, participant responses were synthesized, and changes were made to the game for the next session. After the end of the design phase, the therapy video game with integrated biofeedback was ready for testing in-home.

Biofeedback Implementation

A sample of the biofeedback elements can be seen in this video. Game elements were added to connect the therapeutic and recreational goals:

1. Rewards (points, trophies, characters) were given based on movement quality and timing:

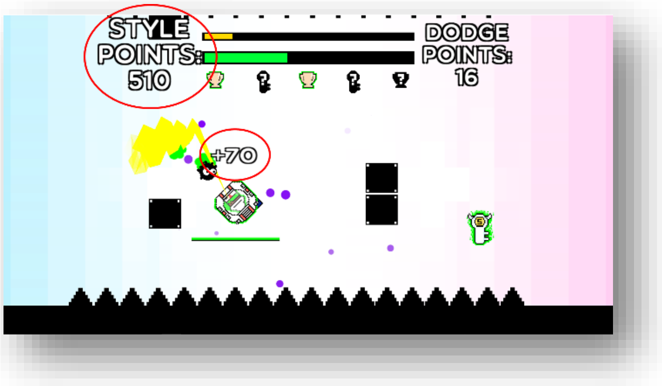

2. Instructional tutorial panes were inserted and appeared as the participant needed them:

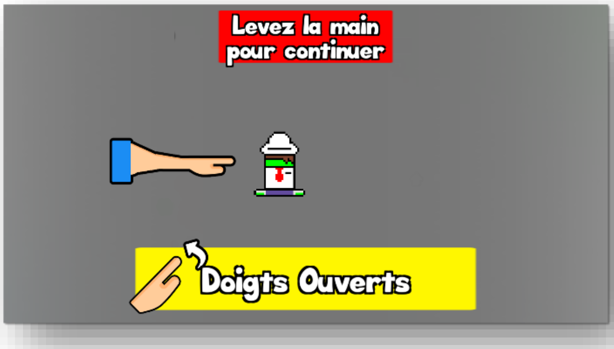

3. Participants had the choice to review feedback and some biofeedback elements were removed based on the participants progress/ proficiency:

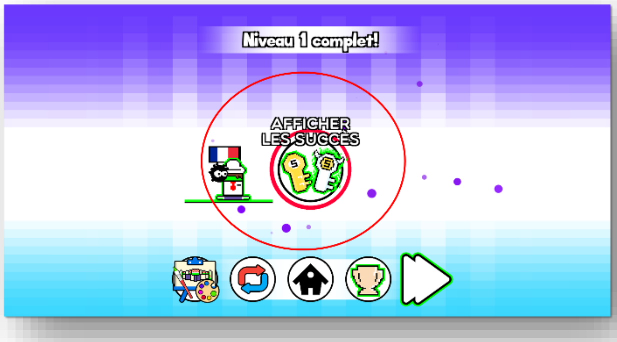

Biofeedback use

After using the system at home for four weeks, the following observations were made regarding use of the new biofeedback elements:

- Participants immediately changed their movements in response to biofeedback while playing. Wrist movement was more isolated directly after a biofeedback prompt. The forearm resultant acceleration was 10.2±4.0% lower directly after biofeedback.
- Participants scored points more quickly and required fewer tutorials, even after advancing to more difficult levels. After practicing, in a typical 2-minute level the average participants scored 39% (+11) more points related to movement timing, 8% (+131) more style points related to movement quality and needed 20% (-1) fewer practice panels.
- Participants reviewed their biofeedback 65.4±22.4% of the time at the end of the level demonstrating autonomy and active engagement with the system.

Details of this co-creation process, its findings and use of biofeedback during home practice are in a manuscript under revision: MacIntosh, Vignais, Vigneron, Fay, Musielak, Desailly, Biddiss. The design and evaluation of biofeedback in motor therapy gaming. *Assistive Technology. [Submitted June 2019].*

Reference

1. MacIntosh A, Lam E, Vigneron V, Vignais N, Biddiss E. Biofeedback interventions for individuals with cerebral palsy: a systematic review. Disabil Rehabil. 2019;41: 2369–2391. doi:10.1080/09638288.2018.1468933
