# Supplementary material for: A biofeedback-enhanced therapeutic exercise video game intervention for young people with cerebral palsy: A randomized single-case experimental design feasibility study
Source: PLoS One. 2020 Jun 22;15(6):e0234767. doi: 10.1371/journal.pone.0234767 (PMC7307764; doi:10.1371/journal.pone.0234767)
Supplement: S2 File — (PDF) [file pone.0234767.s002.pdf]

The Single-Case Reporting guideline In BEhavioural interventions (SCRIBE) 2016 Checklist

Item number	Topic	Item description	Notes
TITLE and ABSTRACT			
1	Title	Identify the research as a single-case experimental design in the title	p1
2	Abstract	Summarise the research question, population, design, methods including intervention/s (independent variable/s) and target behaviour/s and any other outcome/s (dependent variable/s), results, and conclusions	p1-2
INTRODUCTION			
3	Scientific background	Describe the scientific background to identify issue/s under analysis, current scientific knowledge, and gaps in that knowledge base	p3-4
4	Aims	State the purpose/aims of the study, research question/s, and, if applicable, hypotheses	p4-5
METHODS			
	DESIGN		
5	Design	Identify the design (e.g., withdrawal/reversal, multiple-baseline, alternating-treatments, changing-criterion, some combination thereof, or adaptive design) and describe the phases and phase sequence (whether determined *a priori* or data-driven) and, if applicable, criteria for phase change	p5-6
6	Procedural changes	Describe any procedural changes that occurred during the course of the investigation after the start of the study	p6
7	Replication	Describe any planned replication	N/A
8	Randomisation	State whether randomisation was used, and if so, describe the randomisation method and the elements of the study that were randomized	p8
9	Blinding	State whether blinding/masking was used, and if so, describe who was blinded/masked	p6
	PARTICIPANT/S or UNIT/S		
10	Selection criteria	State the inclusion and exclusion criteria, if applicable, and the method of recruitment	p6-7
11	Participant characteristics	For each participant, describe the demographic characteristics and clinical (or other) features relevant to the research question, such that anonymity is ensured	p15
	CONTEXT		
12	Setting	Describe characteristics of the setting and location where the study was conducted	p6
	APPROVALS		
13	Ethics	State whether ethics approval was obtained and indicate if and how informed consent and/or assent were obtained	p6
	MEASURES and MATERIALS		
14	Measures	Operationally define all target behaviours and outcome measures, describe reliability and validity, state how they were selected, and how and when they were measured	p10-12
15	Equipment	Clearly describe any equipment and/or materials (e.g., technological aids, biofeedback, computer programs, intervention manuals or other material resources) used to measure target behaviour/s and other outcome/s or deliver the interventions	p8-9
	INTERVENTIONS		
16	Intervention	Describe intervention and control condition in each phase, including how and when they were actually administered, with as much detail as possible to facilitate attempts at replication	p8-9
17	Procedural fidelity	Describe how procedural fidelity was evaluated in each phase	p6,8
	ANALYSIS		
18	Analyses	Describe and justify all methods used to analyse data	p8
RESULTS			
19	Sequence completed	For each participant, report the sequence actually completed, including the number of trials for each session for each case. For participant/s who did not complete, state when they stopped and the reasons	p14,17
20	Outcomes and estimation	For each participant, report results, including raw data, for each target behaviour and other outcome/s	p14,17
21	Adverse events	State whether or not any adverse events occurred for any participant and the phase in which they occurred	p14
DISCUSSION			
22	Interpretation	Summarise findings and interpret the results in the context of current evidence	p19
23	Limitations	Discuss limitations, addressing sources of potential bias and imprecision	p22-23
24	Applicability	Discuss applicability and implications of the study findings	p19-22
DOCUMENTATION			
25	Protocol	If available, state where a study protocol can be accessed	p2
26	Funding	Identify source/s of funding and other support; describe the role of funders	positioned by journal
