# Supplementary material for: A biofeedback-enhanced therapeutic exercise video game intervention for young people with cerebral palsy: A randomized single-case experimental design feasibility study
Source: PLoS One. 2020 Jun 22;15(6):e0234767. doi: 10.1371/journal.pone.0234767 (PMC7307764; doi:10.1371/journal.pone.0234767)
Supplement: S4 File — (PDF) [file pone.0234767.s004.pdf]

S4 Procedural Fidelity Resource

Contents

1 First Visit ... 2
1.1 SFC resource ... 3
2 Reference Phase ... 6
2.1 Baseline data collection ... 6
2.2 Game setup ... 7
3 Intervention Phase ... 8
3.1 Weekly Check-In ... 8
3.1.1 Measurements ... 8
3.1.2 Gameplay ... 8
3.1.3 Solution-Focused Coaching: EARS Check-in conversation pattern ... 9
4 Post-Intervention ... 10
4.1 Recover hardware data ... 10
4.2 Final discussion ... 10
4.2.1 Open Interview Questions Resource ... 11
4.3 Impressions of protocol – Phone call resource ... 12

1 First Visit

- ☐ Discuss intervention using SFC resource
- ☐ Assess COPM
- ☐ Assess AHA
- ☐ Assess B&B

1.1 SFC resource

1. Introduce the find goals

a. Appreciate their participation

« Thank you for coming and agreeing to participate »
« What would you be doing if you were not here today? »

b. Explore and define motivations for participating

   i. Cycle of exploration of motivation (see page 3)
   ii. There is a link between the actions in the game and the movements that you do everyday
   iii. Present the game, if useful

« By plying the game, you do some movements that you use everyday such as …. »
« …. Are there other actions or movements that you do regularly? [confirm their understanding] »

« What are your best hopes for participating in the study? »
« Imaging that tomorrow, all your best hopes were realized, what would be different for you? »

c. COPM:
Immediately ask for COPM goal area and scaling questions

« On a scale of 10 to 1, where 10 is you are [goal specific], and 1 is the opposite, where are you now? …And how satisfied are you with that score, on a scale of 1 to 10? »

Fill in COPM primary goal

   i. Performance (1 to 10)
   ii. Satisfaction (1 to 10)
   iii. Importance (1 to 10)

« You were a 4 on that scale – how did you manage to get to a 4, and not lower? ….and what else?... »
« What would be different at one point higher?... and what else? »

2. Reformulate goals

If I understand you, to be at a 4…. (indirect complement)
And to be at 4 ½ would be …

3. Develop an action plan

a. Planning

Imagine you can look into the future, but on which days in the coming week could you see yourself playing?

When I see you next time, and I ask you on which days you played what will you tell me?
Would it be helpful if we looked at your schedule this coming week and find times when it works well for you to play?
We have a calendar here and you can it in as you like?

b. Finding support strategies

Is there someone who can help you to make sure you follow your action plan? or
Do you want to tell someone what you will be doing? Or do you want to keep your plan to yourself?
How can someone help make sure you follow your action plan?
How and when will you ask them?

4. Confirmation

a. Acknowledge their competencies and resource already in place
b. Review their long-term goals
c. Review their short-term goals and how the game links to this
d. Reaffirm their action plan

5. Evaluate the discussion

a. On a scale from 1 to 10

Have we discussed everything you wanted to today?
Do you feel we understood what you want from participating?

Cycle of exploration of motivation

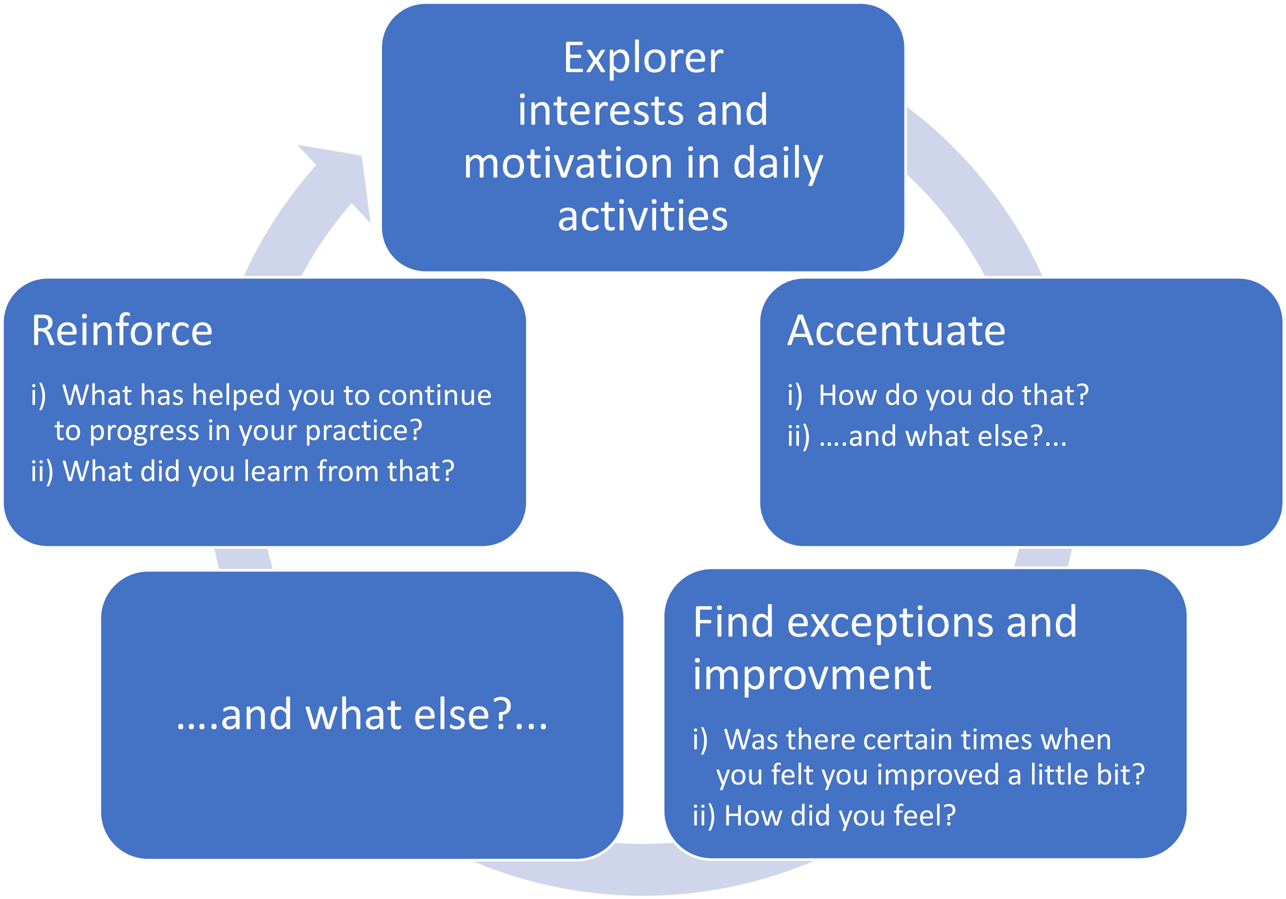

2 Reference Phase

2.1 Baseline data collection

Time and Date:			
	1st	2nd	3rd
AROM (deg) – Open			
AROM (deg) – Closed			
PROM (deg) – Open			
PROM (deg) – Closed			
Grip (mmHg) D			
Grip (mmHg) ND			
Note:			

2.2 Game setup

ASSERT CAN PLAY THE GAME AT HOME:

1. Starting the game
   - ☐ Can put on Myo
   - ☐ Can start MyoDashy
   - ☐ Can close/restart MyoDashy
2. Playing the game
   - ☐ Can select levels
   - ☐ Can pause game
   - ☐ Can return to menu
   - ☐ Can see trophies
   - ☐ Can change characters
3. Closing the system
   - ☐ Can escape a level
   - ☐ Can quit from home screen
4. Maintaining the system
   - ☐ Plugs in Myo
   - ☐ Has instruction manual
5. Take home materials
   - ☐ Laptop Name: ____________________
   - ☐ Laptop power cord
   - ☐ Myo Name: _____________________
   - ☐ USB charger
   - ☐ User Manual

3 Intervention Phase

3.1 Weekly Check-In

3.1.1 Measurements

1. Complete Data Collections forms

    - ☐ Range of motion and Grip
    - ☐ Check-In conversation EARS
    - ☐ SEAS (week 1 and 4 only)

3.1.2 Gameplay

2. Recover data

    - ☐ Copy Results folder form participant computer
    - ☐ Run performance summary for therapists

3. Game Configuration

    - ☐ Edit feedback according to schedule
        - ☐ Untoggle progress bars in week 3
        - ☐ Toggle to always use decision panel in week 4
    - ☐ Change speed as required
    - ☐ Export player config

3.1.3 Solution-Focused Coaching: EARS Check-in conversation pattern

When you think about your practice goals, how do feel you have done this past week, on a scale of 1 to 10, with 10 being what you want, and 1 being the opposite, where are you on the scale right now?

1. Elicit the Exception
    a. What has worked *to help make sure you practice*?
    b. What is better since I last saw you?

2. Amplify the Exception
    a. What did you do *to help make sure you practice*? How did you do that?
    b. How have you managed *to make sure you practiced this past week*?
    c. What might others say you've done to help *make sure you practiced this past week*?
    d. What was helpful for you along the way? What else…?
    e. What is different for you as a result of this? What else…?

3. Reinforce the Successes and Strengths
    a. What can help you maintain these improvements *in how you have been practicing*?
    b. What have you learned as a result?

4. Start Over
    a. Given what you've been learning, what's needed now *to make sure you meet your practice goal next week*?

4 Post-Intervention

4.1 Recover hardware data

- ☐ Participant returned take-home material:
  - ☐ Laptop Name: ___________________________________
  - ☐ Laptop power cord
  - ☐ Myo Name: _________________________________
  - ☐ USB charger
- ☐ Copy Results folder form participant computer
- ☐ Run performance summary for therapists

4.2 Final discussion

- ☐ Discussion - Use the SFC coaching checklist
- ☐ Reassess COPM
- ☐ Reassess AHA
- ☐ Reassess B&B
- ☐ Get game Feedback - Open Interview Questions resource
- ☐ Impression call (Post final discussion meeting) – Impressions resource

4.2.1 Open Interview Questions Resource

6. USIBILITY

Ease of play

1. *What helped to make the game easier to play?*
2. *What helped to make the game harder to play?*
3. *Did you feel like you were in control of the game?*
4. *Did anything hold you back from playing the way you wanted?*

Strategy

5. *Was there anything that you used when playing the game to help you succeed?*
6. *Did the score change how you played the game?*
7. *Did the characters change how you played the game?*
8. *What was your strategy to avoid the obstacles?*
9. *Name the game you have played that is most similar to this game.*

Focus

10. *What was the most important part of the game for you?*
11. *If you could change any aspect of the game or your experience, what would it be? Unlimited budget and time.*

Open Interview Questions (2 of 2)

2. FUN

a. Find exception and improvement

1. What parts of the game did you find most fun?
2. What was your favorite moment or interaction?
3. What was your least favorite moment or interaction?
4. Which game mode do you like the best? (Jump, Dash, Fly), Why?

4.3 Impressions of protocol – Phone call resource

Impressions of MyoDashy protocol<br><br>Do you think any aspects of MyoDashy were particularly "positive" or "negative" for your children? - Which ones? Why?
Positive<br><br>Has anyone in your family experienced a positive/exciting/enlightening situation using MyoDashy? What happened?
Negative<br><br>Has anyone in your family experienced a difficult/unpleasant situation with MyoDashy? What happened?
Independence<br><br>- How independent was your child in his/her use of MyoDashy?<br>- How involved parents were in setting time to practice?

Impact on family life<br><br>1. How did MyoDashy fit into your family's routine? (when/how much/with who did your child play)<br><br>2. Do you think that your child's use of MyoDashy affected in any way (positive and/or negative) your family life? (E.g. family interaction is decreasing or increasing)
