# Supplementary material for: A biofeedback-enhanced therapeutic exercise video game intervention for young people with cerebral palsy: A randomized single-case experimental design feasibility study
Source: PLoS One. 2020 Jun 22;15(6):e0234767. doi: 10.1371/journal.pone.0234767 (PMC7307764; doi:10.1371/journal.pone.0234767)
Supplement: S6 File — (PDF) [file pone.0234767.s006.pdf]

Protocol

"Myo Dashy"

Biofeedback-enhanced interactive computer-play for youth with cerebral palsy: a feasibility pilot study measuring the effect of play on wrist and hand activity.

November 23, 2018

Contents

1. Abstract : ... 3
2. Introduction : ... 4
   2.1 Cerebral Palsy ... 4
   2.2 Rehabilitation strategies ... 4
   2.3 Solution Focused Coaching ... 4
   2.4 Previous work ... 5
   2.5 Rationale ... 5
3. Objectives : ... 7
   3.1 Primary ... 7
   3.2 Secondary ... 7
4. Methods : ... 7
   4.1 Study design ... 7
   4.2 Participants ... 7
   4.3 Eligibility criteria ... 8
   4.4 Recruitment ... 9
   4.5 Pilot feasibility study overview ... 9
   4.6 ICP intervention game description ... 12
   4.7 Outcome measures ... 13
5. Analysis : ... 15
6. Significance : ... 15
7. Study Timeline : ... 16
8. Budget : ... 16
9. References : ... 17
10. Appendices: ... 21
   10.1 Appendix 1: Intervention Schedule ... 21
   10.2 Appendix 2. Guiding the initial dialogue ... 24
   10.3 Data collection link to questions ... 25
   10.4 Appendix 3: Check-in conversation resource ... 27
   10.5 Appendix 4a: Conversation additional resources ... 27
   10.6 Appendix 4b: Conversation additional resources (Figure 1) – Example action plan ... 29
   10.7 Appendix 5: Discussion resources ... 29
   10.8 Appendix 6: Template data collection forms ... 32

1. Abstract :

The protocol aims to evaluate the feasibility and potential efficacy of an Interactive Computer Play (ICP) intervention. The ICP intervention is built to help youth with Cerebral palsy (CP) who have difficulty performing activities of daily living with their hand.

The ICP intervention is a video game controlled by performing gestures with the non-dominant hand. Using Low-cost commercial technology (Myo Armband, Thalmic Labs) muscle activity and arm movement is used to recognize the gestures which control the game. Players will get feedback in the game about the quality of their movements through the built-in points and rewards system. This repetitive practice and feedback will help the participants build strength and control in their arm. To evaluate this ICP intervention, 10 participants, with hemiplegic CP and 8-18 years old, from Holland Bloorview will be recruited for a pilot feasibility study using a single-case experimental design (SCED). The design is as follows:

1. Phase 1. Participants will speak with therapists / researchers in an Initial Dialogue to:
    a. Introduce the study/game and what it offers types of daily activities
    b. Set Occupational Performance Measure (COPM) goal areas, and
    c. Develop an action plan to facilitate the successful achievement of their goals.

2. Phase 2. Participants will perform baseline functional assessments including: active range of motion (AROM), Assisting Hand Assessment (AHA), Box and Blocks Test (B&B). They will also have precatory training to make sure they understand how to run and play the game at home.

3. Phase 3. During the 4-week intervention, participants will play the ICP game from their home according to the goals they define during the initial dialogue. This is expected to be 20-30 min * 5 days per week. Once per week, participants will play the ICP intervention with a researcher in clinic or at home who will also measure AROM while recording the play session.

4. Phase 4. After the intervention, participants will complete clinical measures of functional performance (AROM, AHA, B&B) a final time and speak with therapist and researcher to re-evaluate COPM goals and give their subjective evaluations of the game.

By leveraging the motivational and immersive aspects of ICP and combining it with evidence-based movement feedback this protocol has the potential to improve home-based ICP therapies for persons with CP.

2. Introduction :

2.1 Cerebral Palsy

Cerebral palsy (CP) is a common disability related to an injury or abnormality of the brain occurring near birth which persists from childhood through adulthood [1]. CP can impact a person's motor control, perception, intellectual function, ability to perform daily activities (e.g. walking, eating), and participation in society [2]. Persons with CP can have impaired hand or arm function and it is important to address strategies for improving motor activities.

Motor activities may be affected by many factors including sensory deficits, biomechanical and postural limitations. Impairment can be a result of both positive and negative motor signs [3]. Positive motor signs are those that create an unintended increase in activity, such as spasticity or dystonia. Conversely, negative motor signs are those related to insufficient activity, such as weakness or impaired selective motor control [4]. Since these presentations can be linked, it is important to consider intervention strategies that address both [4].

2.2 Rehabilitation strategies

Rehabilitative intervention strategies for improving motor activities can involve intense practice. For hemiplegia and spastic triplegia, concentrated use of the non-dominant limb is common. In addition to frequent and intense practice, motor activities are influenced by: the focus of attention during training, the motivation during practice and the feedback they receive [5].

Feedback can help increase awareness and control that would normally be unnoticed [6]. Biofeedback is a form of feedback, where information about a motor performance is communicated back to the individual. An example of this would be the speed at which someone completed a task like grabbing an moving an object. This can be particularly helpful for some persons with CP who may have sensory impairments and are less able to use intrinsic feedback [1]. Additionally, persons with CP have shown difficulty with movement initiation and prediction [7], and a greater reliance on visual strategies [8]. Given the extent of motor and sensory deficits, biofeedback is well suited to enhance quality of training. As such, it is important to identify biofeedback interventions effective in improving motor activities for persons with CP.

Similarly, Interactive computer play (ICP) can enhance quality of training. ICP is "any kind of computer game or virtual reality technology where the individual can interact and play with virtual objects on a computer generated environment" [9]. For this project, we will use ICP in the form of a video game where a player will do therapeutic movements to control game actions on-screen. ICP can provide a motivational, goal-based environment, where motor signs including muscle weakness and selective motor control are addressed.

To see changes in motor activities, a significant practice is often necessary. However, low adherence is a concern to the potential efficacy of exercise interventions, and therapeutic ICP is no exception [10]. Adherence in home-based interventions has historically ranged from 34-67% [11,12]. In a previous upper limb ICP intervention, Bilde et al (2011) found that children trained for the prescribed time on 62% of the total training days [13]. To maintain adherence researchers and practitioners have attempted many strategies including: self-monitoring, coaching, counselling and monitoring, rewards, increasing the intervention in small increments and cognitive behavioural therapy [14,15].

2.3 Solution Focused Coaching

Recent developments in coaching have shown promise for promoting an individual's investment in physical activity interventions. Solution-focused coaching in pediatric rehabilitation (SFC-Peds) is a model of coaching recommended for youth with disabilities [16]. SFC-Peds uses tools which builds the person's intrinsic motivation and self-determination, leading to genuine interest and engagement in

health behaviour changes [17]. To promote this investment, SFC-Peds has eight evidence-based coaching tenets [18] :

1. taking a client-centered approach
2. collaboration between coach and client
3. promoting client reflection
4. fostering capacity-building
5. ecological (i.e., in the client's environment)
6. strengths-based
7. using positive language and
8. focusing on a client-identified goal

Since SFC-Peds is tailored to the individual it is well suited for a wide range of age and abilities. It has recently been used to develop personal goals and plans for physical activity and diet improvement with people (11-19 years) who have Duchenne muscular dystrophy [19]. This pilot study found clinically significant increases in goal attainment and performance [19].

Practically, SFC-Peds requires coaches to work collaboratively with children to help them envision their "preferred future" [16]. This involves asking the children strategic questions to let the child express their goals and develop their solution plan. Coaches use positive language to highlight the child's successes and focus on what they can do, as opposed what they are limited by. Through this process, therapy goals and supporting plans are developed. The goals and plan are directed by the child, and as such, align with their priorities.

SFC-Peds principles and strategies are being used in this study to:

- take a client-centered approach to identifying goals related to reaching and grasping
- drive and monitor intrinsic motivation
- help participants identify realistic action plans and strategies to support their goals

2.4 Previous work

A client-centered approach is important not only for structuring an intervention, but also for the development of the intervention itself. The participatory design process has, at its centre, the needs of the user in mind [20]. To develop the current ICP intervention, we have consulted individuals with CP and clinicians. Through interviews and game testing sessions the client's priorities have been embedded into the core of the game.

Development of the IPC intervention started during the summer of 2017 at Holland Bloorview Kids Rehabilitation Hospital and continued throughout 2018 at Fondation Ellen Poidatz, Saint Fargeau Ponthierry, France. During each game design and testing session, participants used a muscle activity sensor on the forearm to control actions on-screen in a video game. Participants gave their opinions of the game and of how it felt to use the controller. From these opinions, we have improved the sensitivity of the controller and the game content.

2.5 Rationale

The goal of this project is to improve reach and grasp activities in youth with cerebral palsy by leveraging:

- Motivational and immersive ICP
- Evidence-based biofeedback approaches, and
- Solution Focused Coaching principles that strengthen intrinsic motivation

The ICP intervention requires participants to practice therapeutic movements such as wrist extension or finger-thumb pinching. Forearm muscle activity during these movements are recorded and used to

control the game. Intuitive biofeedback will be provided through visual and audio cues in the form of game rewards. Solution-Focused Coaching to help participants identify their motivation around the intervention and build support plans to help them achieve their training goals. This project will provide the framework for effective home-based therapies for persons with CP.

3. Objectives :

3.1 Primary

A. To determine the impact of a biofeedback and coaching home-based interactive computer-play intervention on active range of motion (AROM) of the wrist for youth with cerebral palsy.

3.2 Secondary

B. To evaluate if a biofeedback-enhanced interactive computer play activity is a feasible approach to improve wrist extension and grasping goals in youth with cerebral palsy.

C. To explore the potential impact of biofeedback-enhanced interactive computer-play on wrist motion and hand function.

Evaluations of each objective are specified in the following section: Outcome Measures

4. Methods :

4.1 Study design

The pilot feasibility study will be conducted a single-case experimental design (SCED). Participants will be randomly assigned to begin the intervention at staggered starting-points [21]. This method is appropriate for analyzing differences between phases, particularly when the intervention is expected to cause a sustained effect and allows for smaller sample sizes (5-10 participants)[22]. Before the intervention, participants will meet with the researcher and therapist to complete an initial dialogue. The discussion will be in a Solution-focused Coaching (SFC-Peds) style and will serve to help participants identify their motivation around participating in the intervention and to specify their training goals. A summary of the study process can be seen in Figure 1 below, with corresponding details in the following sections:

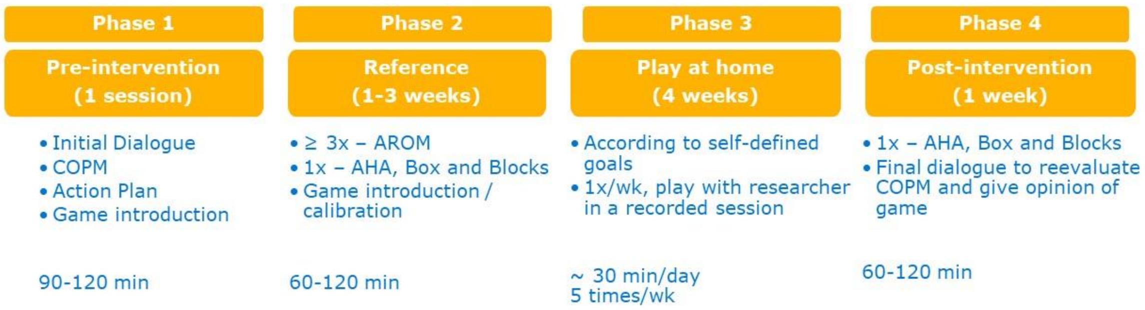

Figure 1: Summary of pilot feasibility study process and elements. Items in yellow are the study phases and duration. Items in blue indicate the tasks during each phase. See Appendix 1 for full details.

4.2 Participants

Ten youth with CP will be recruited from the client base at Holland Bloorview. This sample size is in line with the pilot nature of this work, the SCED study design and financial resources available. While

sample size calculations are not necessarily required for feasibility studies, Table 1 below, provides varying estimates of the effect of biofeedback interventions on wrist active range of motion. This table is used to acknowledge the uncertainty surrounding the pilot estimates of effect for active range of motion at the wrist, based on similar studies [23]. Ten participants will also be recruited at Fondation Ellen Poidatz Saint Fargeau Ponthierry, France.

Table 1: Sample size estimates and variability in active range of motion at the wrist pre-post-biofeedback intervention.

	Observed changes		Desired change range					
	Literature Min[24]	Literature Max[25]	5°	5°	10°	10°	20°	20°
Pre-post change (°)	6.3	18.2	5	5	10	10	20	20
Expected variability (SD)	3.6	12.2	3.6	12.2	3.6	12.2	3.6	12.2
N- required	7	9	23	80	8	22	4	8

Four similar studies identified through literature [24–27]. Sample size estimates based on dependent t-test, competed with Gpower3 (v.3.1)[28].

4.3 Eligibility criteria

Inclusion criteria:

1. Clinical diagnosis of Cerebral Palsy.
2. Age: 8-18 years. This age range was selected given the popularity of video games and the size of the muscle sensors available.
3. Manual Abilities Classification System levels I-III [29].
4. Have a goal relating to improving hand / wrist function.
5. Dominantly spastic presentation (as confirmed by The Hypertonia Assessment Tool[‡])
6. Able to co-operate, understand, and follow simple instructions for game play. This will be assessed during the information and assent process by the researcher.
7. Having passive ROM of at least 10° greater than AROM.

Exclusion criteria:

1. History of unmanaged epilepsy. Video game systems are not recommended for individuals with a history of epilepsy as per manufacturer's Health and Safety Precautions.
2. Has received a Botulinum Toxin treatment within 3 months or constraint-based movement therapy within 6 months of the study enrollment.
3. Visual, cognitive or auditory disability at a level that would interfere with game play. The child must have normal or corrected to normal vision and hearing.
4. Dominantly dystonic presentation (as confirmed by the Kinematic Dystonia Measure[*])
5. Unable to commit an estimated minimum of 10 hours to their training plan over four weeks.

Participants will not be required to stop any usual care. However, if the participant decides to complete botulinum toxin injections or surgery on the affected upper limb, they may be withdrawn

from the study. Participants who have such a procedure scheduled during the intervention may be excluded.

ǂ The Hypertonia Assessment Tool (HAT) will be used to confirm the presence of spasticity. The HAT is a 7-item tool developed to discriminate between spasticity, dystonia and rigidity. The test takes five minutes to complete. In children and youth with CP (4-19 years old) the HAT has shown good interrater reliability (prevalence-adjusted bias-adjusted kappa (PABAK) = 0.43-1.0) and validity (PABAK = 0.43-1.0) [30,31].

* The Kinematic Dystonia Measure (KDM) quantifies the amount of involuntary movement in a dystonic upper extremity during voluntary actions [32]. In the test, the subject taps the unaffected hand to the beat of an auditory cue, abnormal posturing and movement in the dystonic arm is recorded using reflective markers. Joint positions and angular rotations of extremity are recorded through a motion capture system. These changes in position and angles are summed to produce a Kinematic Dystonia Measure score. Test-retest reliability of the KDM was excellent (CCI = 0.95). KDM also correlated with total Barry-Albright Dystonia Scale scores (Pearson r = 0.79, P = .003).

4.4 Recruitment

Researchers will make initial contact with potential participants identified through the HEMI-NET database and Connect2Research database. Only individuals who have agreed to be contacted for future studies will be passed to the researchers. Recruitment material will be sent by mail to the identified families (Flyer, Information letter). An opt-out number will be provided for individuals who do not wish to be contacted. One week after the recruitment materials have been sent, potential participants (those who have not opted out) will receive a follow-up phone call by a researcher, unknown to the participant. The researcher will inform them of the study and offer participation using the Script- researcher.

The Occupational and Physical Therapists of the Neuromotor team will be asked to identify potential participants from their client base. To identify potential participants the researcher will meet with and give the therapists: a summary of the study protocol and objectives, the study selection criteria, and the recruitment Flyer. The process and materials have been developed with the clinician-scientists working on this project, Linda Fay. Therapists will make initial contact by phone or in person, depending on the prospective participants next scheduled visit (see 'Script – Therapist').

Therapists will inform the potential participant about the study and offer the recruitment Flyer and Information letter. If a potential participant agrees, the therapist will forward the participant's contact information to the researcher who will follow-up with a phone call using the 'Script-researchers'.

The parent will be consulted to ensure that the child can provide consent or assent. The researcher will also talk with the child and ask him/her questions about the research study to ensure that they understand the information presented and would like to participate in the study. If any hesitation or anxiety is apparent, then the child will not be asked to participate. If the child appears capable they will be asked to provide consent/assent. The parent will also be asked to complete a consent form, if they are willing to have their child participate in the study.

4.5 Pilot feasibility study overview

Phase 1: Pre-Intervention

Before the intervention, participants will meet with the researcher and therapist for approximately 90 minutes to complete an initial discussion. The discussion will be in a Solution-focused Coaching

(SFC-Peds) style and will serve to help participants identify their motivation around participating and to specify their training goals.

The initial Solution-Focused Coaching style dialogue in serves to improve home-play adherence by:

1) Having the client identify intrinsic motivations for participating
2) Letting the client build their own practice schedule for home-play
3) Helping the client set practical goals relevant to everyday activities that playing the game may help to improve (e.g. making it easier to pull up a zipper on their jacket.)

The process of the initial dialogue is as follows:

a) Introduce the study/game and what it offers

The introduction will serve to remind the participant of the *purpose of the study and frame the conversation* and goal setting accordingly. They will see how the game is played, ask questions and talk about the types of daily, real-life, activities the intervention may relate to (related to reach and grasp function).

b) Set COPM goal areas within the context of the study

Then the *COPM goal areas* will be identified used SFC style questioning. Along with the performance and satisfaction of each goal, participants will discuss how they have been successful in the past, envision their preferred future, and reaffirm their goals.

c) Develop an action plan

Finally, during the conversation, an *action plan* will be clarified with the client and recorded by the coach. Given the notes/ information given by the client during the conversation, the participant will reflect on their goals and identify a training schedule or practice goal for the intervention. Acknowledging that developing strict schedules may be helpful for some but counter-productive for others, the coach may ask questions or present strategies to help the participant construct their practice goal for the intervention. Coaches may use light suggestive questions or calendar templates Appendix 4: Conversation additional resources (Figure 1), according to the needs of the participant.

There will not be a standard script, however coaches will be trained in this SFC framework and guide the conversation using Appendix 2. Guiding the initial dialogue. The exact phrasing will be adjusted according to the person and their goals.

The data elements collected during initial conversation, along with the link to SFC principles, can be seen in Appendix 2. Guiding the initial dialogue (Table 1). Coaches will also draw from SFC-training provided by Holland Bloorview Kids Rehabilitaiton Hospital resources to guide conversations: Appendix 4: Conversation additional resources.

Before playing the game at home, participants will also undergo preparatory training and familiarization to calibrate the game system. Participant will learn how to set up the game and maintain the system. Before being given the system for home play, the participant will demonstrate twice that they are able to setup, start, play, and close the system by themselves. They will learn how the game is played and the features they can unlock as the play. While learning the game they will also be calibrating the controller to their specific muscle activity patterns. We will use this calibration of the participant's gestures to control the game. After programming these controls, the game will be deployed to the computer the participant will use at home to play.

Phase 2: Reference (1-3 weeks, until AROM stability is reached)

a) Participants will complete the active range of motion (AROM) measurement a minimum of three times, to demonstrate stability (i.e. non-significant variability and absence of trends) [33]. The researcher will arrange to meet the participants several times (approximately every 2 days) during the Reference Phase. They may meet at a location convenient to the participant.

b) Participants will complete clinical measures of functional performance one time:
   i) Assisting Hand Assessment (AHA)
   ii) Box and Blocks Test (B&B).
   iii) Finalize Canadian Occupational Performance Measure (COPM) goal areas established during initial discussion.

Details of the usage and psychometric properties of these clinical measures can be found in the following section titled Outcome measures.

After establishing AROM stability, the participant will wait a randomized amount of time (1-10 days) before playing the ICP game at home in Phase 3.

2) Phase 3: Playing ICP game at home

a) Participants will play the ICP game (full description below) from their home according to the goals they defined during the initial dialogue. This is expected to be 30 min * 5 days / week for 4 weeks.

b) Once per week, participants will play the ICP game with a researcher. The sessions are not expected to exceed 60 minutes.
   i) Participant will be video recorded and instrumented with an electro-goniometer at the wrist during play. (30 min)
   ii) AROM will be evaluated before and after the play session (< 5 min).
   iii) Box and Blocks will be evaluated before the play session (<10 min).
   iv) Participants will complete a short questionnaire (~10 min)
      (1) This Self-Reported Experiences of Activity Settings (SEAS) questionnaire addresses their interaction, engagement, and sense of control while playing the ICP game at home. Appendix 5: Discussion Resources
   v) Participants and researchers will have a short (< 5 min) check-in conversation to determine if the participant is satisfied with their progress, gauge their motivation, and help them modify the action plan if necessary. The structure of the check-in conversation follows the SFC approach and the resource to guide the conversation can be found in Appendix 3: Check-in conversation resource.

At the same time as the first AROM assessment, Passive ROM and grip strength will also be measured

Phase 4: Post-intervention

a) Within the week after the intervention end, participants complete clinical measures of functional performance a final time:
   i) Assisting Hand Assessment (AHA)
   ii) Box and Blocks Test (B&B).

b) Participants will have a speak with therapists / researchers for 60 minutes at the end to:

i) Re-evaluate COPM goals using the SFC-peds conversation resources (Appendix 4. Conversation additional resources).

ii) To give their subjective evaluations of the game using a semi-structured interview and questionnaire (Appendix 5: Discussion resources)

c) A separate member of the research team (other than who completed the home visits) will conduct one phone call (5-10 minutes) with the parents of the participant (or if the participant provided consent and completed the assessments alone, they will be called). The researcher will ask the parent/participants questions and take notes on their responses related to the use of the exercise video game system during the past month through a semi-structured interview format. (Appendix 7: Post-intervention phone interview resource)

4.6 ICP intervention game description

To play the game, 'Dashy', participants must:

- Put on the Myo armband
- Open the game on the computer.
- Gameplay involves controlling the character using wrist/hand movements:
  - Resting
  - Extension with fingers flexed
  - Extension with fingers extended
  - Extension with finger-thumb pinching
- The objective of the game is to navigate to the end of the level (1-2 minutes) while avoiding obstacles. To avoid obstacles, participants can make the avatar: stop move, jump, and fly. Each of these actions are caused by a wrist/hand movement.

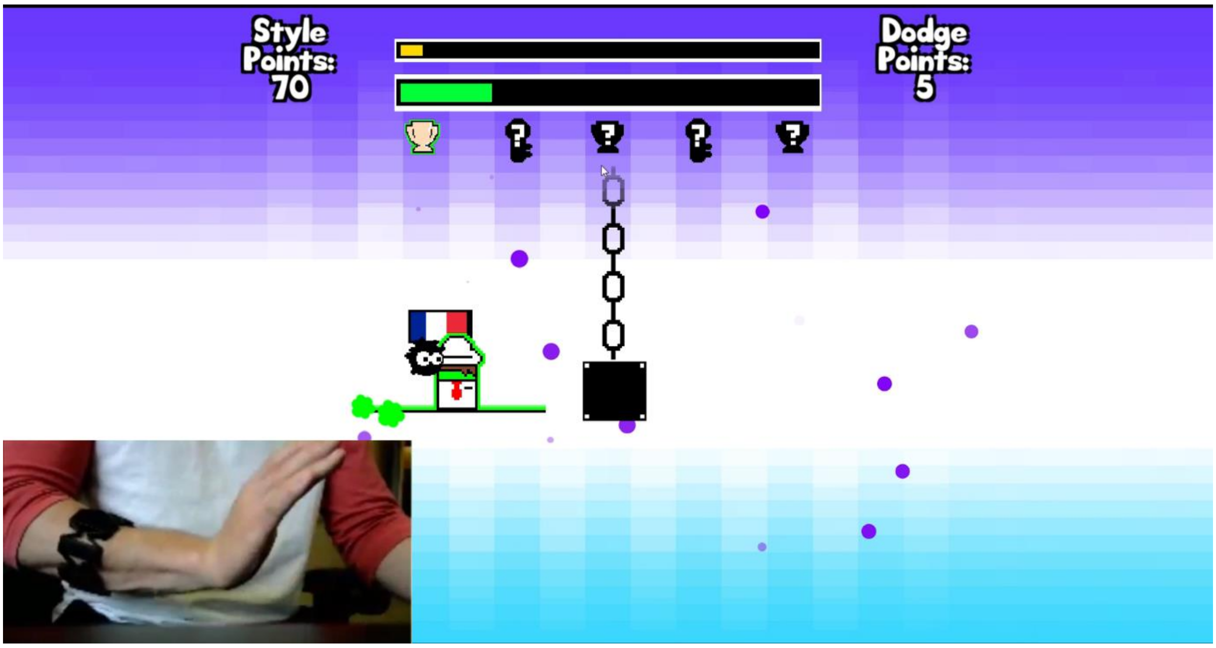

Figure 2: Screen shot if Myo Dashy, the ICP game, with insert of player controlling avatar by extending the wrist.

An example of the game, and how it is controlled can be found here: https://youtu.be/1PI9LDeToLI. The Myo armband collect 8 channels of electromyography (EMG) recordings from around the

forearm, and internal measurements (accelerometer and gyroscope (IMU)). These data are transmitted to the game by Bluetooth (https://www.myo.com/).

4.7 Outcome measures

To the primary objective of determining the impact of the ICP intervention on wrist extension, a goniometric measurement of the wrist AROM will be recorded with and without fingers extended.

Procedure: Wrist extension measurements start with the elbow 90 degrees of flexion, the forearm fully pronated, and the upper arm alongside the trunk. The movements are demonstrated by the assessor after which the child performs the required wrist extension [34]. To keep the test functionally applicable, participants are required to keep their wrist in the same position, but the arm is permitted to move by the elbow and shoulder joints.

Psychometric properties: In the relevant population, AROM has shown test–retest reliability of measuring passive ROM was considered very good (ICCs 0.81–0.94) and interrater reliability correlation coefficients between 0.48–0.73 [35].

Towards the first secondary objective of evaluating the feasibility of the ICP intervention, feasibility criteria have been defied according to best practices[23]. Feasibility studies can reduce the risk of failure for future studies by addressing methodological aspects [36]. Thabane et al (2010) identifies that the purpose of a pilot feasibility study is to asses the: process, resources, management and effectiveness of an intervention. They recommend defining the study objectives in the context of priori success criteria, and reporting results in a standardized manner using the CONSORT format [23].

For this project, we have adopted these recommendations to evaluate the feasibility of the biofeedback-enhanced interactive computer-play for youth with cerebral palsy. The a priori, feasibility success criteria values are based on randomized controlled trial results and feasibility testing of a another upper extremity home based intervention in similar population [13,37] and include:

1) ≥10% response rate from all eligible participants (the minimum feasible response rate for RCTs[38])
2) ≥80% of the participants successfully completed the study. (i.e. completed at least 3 repeated measures during phase A and B, and complete outcome measures and assessments/interviews at both reference and post-intervention)
3) Participants meet their self-identified practice goal. (within ≥66% of the identified frequency or duration)
4) Participants were not prohibited from practicing due to technical constraints (e.g. After instruction, participants could start and play the game, technical challenges were overcome with the provided aid, and they were not forced to cancel a practice session due to technical limitations).

Towards the second secondary objective of exploring the potential impact of the ICP intervention on hand function, the following measures will be evaluated:

-Assisting Hand Assessment (AHA)

Purpose: To quantify functional bimanual performance in youth and adolescents with unilateral CP.

Procedure: Participants complete a sequence of bimanual tasks through the semi-structured progression of a board game (~15 minutes). The board game includes objects that require bimanual handling such as opening a box or shuffling cards. There are 20 tasks, scored on a 4-point scale.

Psychometric properties: In adolescents with unilateral CP up to age 18, AHA shows good construct validity and excellent interrater (ICCs 0.94-0.98) and test-retest reliability (ICCs 0.98-0.99) [39,40].

The most appropriate form of the AHA will be administered according to the participants ability. Generally, for youth 8-12 the 'school kids' version of the AHA will be used whereas for older participants 13-18 years, the adolescent version 'Ad-AHA' will be used [39,40]. For participants who do not have a hemiplegia diagnosis but still have asymmetric hand function an alternative version of the AHA will be used to assess bimanual performance. The Both Hands Assessment (BoHA) was developed from the same concept as the AHA and can be used regardless of the degree of symmetric or asymmetric hand use [41]. The procedure is like the AHA however the scoring items are different. The BoHA is scored on 16-items (11 unimanual, 5 bimanual) using a 4-point rating scale expressed as logit-based BoHA units from 0-100, the same as the AHA. The BoHA has shown good: content validity, item and person reliability.

Smallest detectible change for the AHA is 5 logit units. This value has not been established yet with the BoHA. However, Rasch analysis indicates good potential responsiveness to change, with seven difference performance levels.

-Box and Blocks Test (B&B)

Purpose: To quantify gross manual dexterity.

Procedure: Beginning with the dominant hand, the participant has one minute to move blocks from one side of a box, over a center divider, and place on the other side of the box. The number of blocks that were placed on the opposite side of the box is recorded. The nondominant hand repeats the same process [42].

Psychometric properties: The B&B test shows high interrater reliability (ICCs >0.95) and test-retest reliability (ICCs >0.95) in children 6-19 years [43] and in adults with hemiplegia [44].

-Canadian Occupational Performance Measure (COPM)

Purpose: To evaluate changes in perceived function and satisfaction of performance in self-identified goal areas.

Procedure: COPM goal areas and ratings will be collected during the initial dialogue, as described above in the protocol. The COPM takes 20-40 minutes depending on the engagement of the client. During the conversation with the therapist, participants identify goal areas related to daily activities. They rate the importance of the goal areas along with their performance and satisfaction of the activity from 1-10. At the end of the intervention, the participant re-evaluates their performance and satisfaction for each goal area [45].

Psychometric properties: The COPM has been reported to be responsive to detect clinically significant changes in children with CP [46]. COPM shows good test-retest reliability (spearman rho 0.88-0.89) in adults with stroke [47].

Additional demographic data to be documented includes: gender, age, diagnosis and history of video game use. At the same time as AROM assessments, passive range of motion and grip strength will be collected to be used as complementary measures to AROM.

5. Analysis :

Primary Objective, AROM

Level- and slope-change differences between the reference and home-play phases will be calculated for AROM and compared using the d-statistic [33,48,49]. This analysis offers a standardized measure of the difference between phases between multiple participants [48]. Visual analysis will also be completed to assess the stability and overlap between phases [50]. Single-case experimental design (SCED) is appropriate for analyzing differences between phases in small sample sizes, particularly when the intervention is expected to cause a sustained effect [22].

Secondary Objective, Feasibility

Each a priori feasibility success criteria will be compared to actual observed value. Depending on how closely the observed values match the success criteria, the study will then be given one of the following recommendations [23]:

1. Stop - study not feasible;
2. Continue, but modify protocol - feasible with modifications;
3. Continue without modifications, but monitor closely - feasible with close monitoring; and
4. Continue with- out modifications - feasible as is.

Secondary Objective, Hand Function

Towards the secondary objective of *estimating the treatment effect and its variance,* as opposed to determining definitive changes caused by the intervention, the effect size (Cohen's *d, standardized mean difference*) and 95% confidence intervals will be reported for each measure (AHA, B&B and COPM) [51]. This information facilitates comparison of the effects of different treatments related to hand function, and between similar studies [51]. This information will also inform the sample size required for future randomized controlled trials of this intervention [23].

Additionally, a post-hoc power analysis will be conducted to determine if it is appropriate to evaluate the global changes in functional performance (as measured by AHA, BBT and COPM combined). If sufficient power is found, a multi-variate analysis of variance MANOVA will be conducted. Dependent variables will include scores of each measure AHA, B&B and COPM, and the independent variable will be assessment time (pre- or post-intervention).

Demographic data will also be summarized decrepitly.

6. Significance :

Benefits

Directly within the study, participants may experience measured and perceived improvements in hand function and as assessed by the outcome measures (AROM, AHA, B&B, COPM).

Risks

While exercising, mild muscle strain/ soreness in the upper extremity could be experienced by participants due to frequent use of muscles while playing the ICP intervention. A recent review of similar interventions in this population showed no serious adverse events and infrequent minor reversible events related to exercise such as muscle soreness (MacIntosh et al 2017 submitted to Disability and Rehabilitation). During discussion, participants will be encouraged to reflect on their

habits and motivations. This reflection may unintentionally bring about feelings of discomfort or sadness.

Anticipated results

5-15-degree improvement in AROM after playing the ICP intervention for one month. The intervention is expected to be a feasible option for practicing specific repetitive movements in an engaging environment from the home. Clinically meaningful changes in hand function are not expected due to the relatively short intervention period and specific movement practiced.

Impact

Broadly, the market for rehabilitation therapies is steadily growing alongside the aging population. Supplementing these high-cost services with low-cost, home-based ICP systems may result in considerable cost savings, while providing patients with equal, and in some cases greater, access to rehabilitation therapies. Development of home-based ICP is greatly needed to harness the rehabilitation potential of these technologies and to obtain tangible products that can help individuals meet their rehabilitation goals.

By leveraging the motivational and immersive aspects of ICP and combining it with evidence-based biofeedback approaches this project improves the potential for effective home-based ICP therapies for persons with CP. The process of building user-specific home-based ICP therapies for people with CP can the be expanded to match the increasing market of rehabilitation needs.

7. Study Timeline :

1. September 2018 to October 2018 – Ongoing recruitment
2. October 2018 to December 2018 – Data collection
3. January 2019 - June 2019 – Data analysis
4. August 2019 – Dissemination

8. Budget :

Items	Funds
Software developer	$10,000
Research assistant	$4,000
Occupational Therapist	$3,000
Myo Armbands	$1,400
Laptops to run ICP software	$3,200
Participant Honorariums	$700
Mailing costs for recruitment documents	$100
**TOTAL**	**$22,400**

9. References :

1. Rosenbaum, P. *et al.* A report: The definition and classification of cerebral palsy April 2006. *Dev. Med. Child Neurol.* 2007; 49**,** 8–14
2. Novak, I. *et al.* A systematic review of interventions for children with cerebral palsy: State of the evidence. *Dev. Med. Child Neurol.* 2013; 55**,** 885–910
3. Levin, M. F. & Feldman, A. G. The role of stretch reflex threshold regulation in normal and impaired motor control. *Brain Res.* 1994; 657**,** 23–30
4. Sanger, T. D. *et al.* Definition and classification of negative motor signs in childhood. *Pediatrics* 2006; 118**,** 2159–2168
5. Wulf, G., Shea, C. & Lewthwaite, R. Motor skill learning and performance: a review of influential factors. *Med. Educ.* 2010; 44**,** 75–84
6. Van Dijk, H., Jannink, M. J. a, Hermens, H. J. & Van Dijk, H. Effect of augmented feedback on motor function of the affected upper extremity in rehabilitation patients: a systematic review of randomized controlled trials. *J Rehabil Med* 2005; 37**,** 202–211
7. Green, D. & Wilson, P. H. Use of virtual reality in rehabilitation of movement in children with hemiplegia--a multiple case study evaluation. *Disabil. Rehabil.* 2012; 34**,** 593–604
8. Williams, J. *et al.* A comparison of motor imagery performance in children with spastic hemiplegia and developmental coordination disorder. *J. Clin. Exp. Neuropsychol.* 2011; 33**,** 273–282
9. Sandlund, M., Lindh Waterworth, E. & Häger, C. Using motion interactive games to promote physical activity and enhance motor performance in children with cerebral palsy. *Dev. Neurorehabil.* 2011; 14**,** 15–21
10. Biddiss, E. & Irwin, J. Active video games to promote physical activity in children and youth: a systematic review. *Arch. Pediatr. Adolesc. Med.* 2010; 164**,** 664–672
11. Rone-adams, S. A., Stern, D. F. & Walker, V. Stress and Compliance with a Home Exercise Program Among Caregivers of Children with Disabilities. *Pediatr. Phys. Ther.* 2004; 16**,** 140–148
12. Stergiou, N., Harbourne, R. T. & Cavanaugh, J. T. Optimal Movement Variability: A New Theoretical Perspective for Neurologic Physical Therapy. *J. Artic. Biomech. Res. Build. J. Artic. Pap.* 2006; 9**,**
13. Bilde, P. E. *et al.* Individualized, home-based interactive training of cerebral palsy children delivered through the Internet. *BMC Neurol.* 2011; 11**,** 32
14. McDonald, H. P., Garg, A. X. & Haynes, R. B. Interventions to Enhance Patient Adherence to Medication Prescriptions. *JAMA* 2002; 288**,** 2868
15. Jordan, J. L., Holden, M. A., Mason, E. E. & Foster, N. E. in *Cochrane Database of Systematic Reviews* (ed. Jordan, J. L.) (John Wiley & Sons, Ltd, 2010). doi:10.1002/14651858.CD005956.pub2
16. Baldwin, P. *et al.* Solution-Focused Coaching in Pediatric Rehabilitation: An Integrated Model for Practice. *Phys. Occup. Ther. Pediatr.* 2013; 33**,** 467–483
17. Abraham, C., Kelly, M. P., West, R. & Michie, S. The UK national institute for health and clinical excellence public health guidance on behaviour change: A brief introduction.

doi:10.1080/13548500802537903

18. Schwellnus, H., King, G. & Thompson, L. Client-centred coaching in the paediatric health professions: a critical scoping review. *Disabil. Rehabil.* 2015; 37**,** 1305–1315

19. McPherson, A. C. *et al.* A feasibility study using solution-focused coaching for health promotion in children and young people with Duchenne muscular dystrophy. *Dev. Neurorehabil.* 2017; 00**,** 1–10

20. Schuler, D. & Namioka, A. *Participatory design: Principles and practices*. (L. Erlbaum Associates, 1993).

21. Kratochwill, T. R. & Levin, J. R. Enhancing the scientific credibility of single-case intervention research: Randomization to the rescue. *Psychol. Methods* 2010; 15**,** 124–144

22. Smith, J. D. Single-case experimental designs: A systematic review of published research and current standards. *Psychol. Methods* 2012; 17**,** 510–550

23. Thabane, L. *et al.* A tutorial on pilot studies: the what, why and how. *BMC Med. Res. Methodol.* 2010; 10**,**

24. Olama, K., Hegazy, F. & Thabt, N. Combined effects of myofeedback and isokinetic training on hand function in spastic hemiplegic children. *Egypt. J. Med. Hum. Genet.* 2012; 13**,** 183

25. Rios, D. C. *et al.* NeuroGame Therapy to improve wrist control in children with cerebral palsy: a case series. *Dev. Neurorehabil.* 2013; 16**,** 398–409

26. Wood, K. C., Lathan, C. E. & Kaufman, K. R. Feasibility of gestural feedback treatment for upper extremity movement in children with cerebral palsy. *IEEE Trans. Neural Syst. Rehabil. Eng.* 2013; 21**,** 300–305

27. Sevick, M. *et al.* Using Free Internet Videogames in Upper Extremity Motor Training for Children with Cerebral Palsy. *Behav. Sci. (Basel).* 2016; 6**,** 10

28. Faul, F., Erdfelder, E., Lang, A.-G. & Buchner, A. G*Power 3: a flexible statistical power analysis program for the social, behavioral, and biomedical sciences. *Behav. Res. Methods* 2007; 39**,** 175–91

29. Eliasson, A.-C. *et al.* The Manual Ability Classification System (MACS) for children with cerebral palsy: scale development and evidence of validity and reliability. *Dev. Med. Child Neurol.* 2006; 48**,** 549

30. Jethwa, A. *et al.* Development of the Hypertonia Assessment Tool (HAT): a discriminative tool for hypertonia in children. *Dev. Med. Child Neurol.* 2010; 52**,** e83–e87

31. Knights, S., Datoo, N., Kawamura, A., Switzer, L. & Fehlings, D. Further Evaluation of the Scoring, Reliability, and Validity of the Hypertonia Assessment Tool (HAT). *J. Child Neurol.* 2013; 00**,** 1–5

32. Kawamura, A., Klejman, S. & Fehlings, D. Reliability and Validity of the Kinematic Dystonia Measure for Children With Upper Extremity Dystonia. *J. Child Neurol.* 2012; 27**,** 907–913

33. Solanas, A., Manolov, R. & Onghena, P. Estimating slope and level change in N = 1 designs. *Behav. Modif.* 2010; 34**,** 195–218

34. Aarts, P. B., Jongerius, P. H., Geerdink, Y. A., van Limbeek, J. & Geurts, A. C. Modified Constraint-Induced Movement Therapy combined with Bimanual Training (mCIMT–BiT) in children with unilateral spastic cerebral palsy: How are improvements in arm-hand use established? *Res. Dev. Disabil.* 2011; 32**,** 271–279

35. Klingels, K. *et al.* Upper limb motor and sensory impairments in children with hemiplegic cerebral palsy. Can they be measured reliably? *Disabil Rehabil* 2010; 32**,** 409–416

36. Leon, A. C., Davis, L. L. & Kraemer, H. C. The role and interpretation of pilot studies in clinical research. *J. Psychiatr. Res.* 2011; 45**,** 626–9

37. S., J. *et al.* Randomized controlled trial of web-based multimodal therapy for unilateral cerebral palsy to improve occupational performance. *Dev. Med. Child Neurol.* 2015; 57**,** 530–538

38. Rothwell, P. M. Factors That Can Affect the External Validity of Randomised Controlled Trials. *PLoS Clin. Trials* 2006; 1**,** e9

39. Louwers, A., Krumlinde-Sundholm, L., Boeschoten, K. & Beelen, A. Reliability of the Assisting Hand Assessment in adolescents. *Dev. Med. Child Neurol.* 2017; 59**,** 926–932

40. Louwers, A., Beelen, A., Holmefur, M. & Krumlinde-Sundholm, L. Development of the Assisting Hand Assessment for adolescents (Ad-AHA) and validation of the AHA from 18 months to 18 years. *Dev. Med. Child Neurol.* 2016; 58**,** 1303–1309

41. Elvrum, A.-K. G., Zethræus, B.-M., Vik, T. & Krumlinde-Sundholm, L. Development and Validation of the Both Hands Assessment for Children With Bilateral Cerebral Palsy. *Phys. Occup. Ther. Pediatr.* 2017; 1–14 doi:10.1080/01942638.2017.1318431

42. Wagner, L. V. & Davids, J. R. Assessment Tools and Classification Systems Used For the Upper Extremity in Children With Cerebral Palsy. *Clin. Orthop. Relat. Res.* 2012; 470**,** 1257–1271

43. Mathiowetz, V., Volland, G., Kashman, N. & Weber, K. Adult Norms for the Box and Block Test of Manual Dexterity. *Am. J. Occup. Ther.* 1985; 39**,** 386–391

44. Platz, T. *et al.* Reliability and validity of arm function assessment with standardized guidelines for the Fugl-Meyer Test, Action Research Arm Test and Box and Block Test: a multicentre study. *Clin. Rehabil.* 2005; 19**,** 404–411

45. Law, M. *et al.* The Canadian occupational performance measure: an outcome measure for occupational therapy. *Can. J. Occup. Ther. Rev. Can. d Ergotherapie* 1990; 57**,** 82–87

46. Sakzewski, L., Boyd, R. & Ziviani, J. Clinimetric properties of participation measures for 5 to 13 year old children with cerebral palsy: a systematic review. *Dev. Med. Child Neurol.* 2007; 49**,** 232–240

47. Cup, E. H. C., Scholte op Reimer, W. J. M., Thijssen, M. C. E. & van Kuyk-Minis, M. A. H. Reliability and validity of the Canadian Occupat189ional Performance Measure in stroke patients. *Clin. Rehabil.* 2003; 17**,** 402–409

48. Shadish, W. R., Hedges, L. V & Pustejovsky, J. E. Analysis and meta-analysis of single-case designs with a standardized mean difference statistic: A primer and applications. *J. Sch. Psychol.* 2014; 52**,** 123–147

49. Manolov, R., Gast, D. L., Perdices, M. & Evans, J. J. Single-case experimental designs: Reflections on conduct and analysis. *Neuropsychol. Rehabil.* 2014; 24**,** 634–660

50. Kratochwill, T. R. *et al.* What Works Clearinghouse Single-case design technical documentation. 2010;

51. Faraone, S. V. Interpreting estimates of treatment effects: implications for managed care. *P T* 2008; 33**,** 700–711

52. Law, M. *et al.* The Canadian occupational performance measure: an outcome measure for

occupational therapy. *Can. J. Occup. Ther.* 1990; 57**,** 82–87

53. Ratner, H., George, E. & Iveson, C. *Solution Focused Brief Therapy : 100 Key Points and Techniques*. (Routledge, 2012).

54. Berg, I. K. & De Jong, P. Solution-building conversations: Co-constructing a sense of competence with clients. *Fam. Soc.* 1996; 77**,** 376–391

55. De Jong, P. & Berg, I. K. *Interviewing for solutions*.

56. Brooke. Brooke, J.: SUS: A 'quick and dirty' usability scale. *Jordan, P.W., Thomas, B., Weerdmeester, B.A., McClelland, I.L* 1996; dustrypp**,** 189–194

57. Engeser, S. *Advances in flow research*. *Advances in Flow Research* (Springer New York, 2014). doi:10.1007/978-1-4614-2359-1

58. Ni, L. T., Fehlings, D. & Biddiss, E. Design and Evaluation of Virtual Reality-Based Therapy Games with Dual Focus on Therapeutic Relevance and User Experience for Children with Cerebral Palsy. *Games Health J.* 2014; 3**,** 162–171

10. Appendices:

10.1 Appendix 1: Intervention Schedule

Timeline	Activity	Person responsible	Participant time (min)
	RECRUITMENT		
T0	Recruitment initial contact<br>- Identified from therapist caseload	Researcher<br>Therapist	-
+1-week	Contact by phone or in person by researcher<br>- Information<br>- Discuss consent process<br>- Schedule 1st visit	Researcher	10
	BASELINE		
Baseline 1st visit	Initial conversation<br>- Introduction to study<br>- Demonstrate game<br>- COPM<br>- Action plan<br>Baseline assessment<br>- AROM test-1<br>- Game calibration – to develop gesture classification	Therapist/<br>Researcher<br><br><br><br>Researcher/<br>Therapist	90-120
Baseline 2nd visit<br><br><1 weeks past 1st visit	Familiarize with game<br>- Environment<br>- Action instruction<br>Baseline assessments<br>- AROM test-2<br>- Game calibration<br>- AROM test-3	Researcher	30-60
Baseline 3rd visit<br><br><1 weeks past 1st visit	Baseline assessments<br>- AROM test-4 – if needed<br>- AHA<br>- Box and Blocks<br>- AROM test-5 – if needed	Therapist	30-60
	INTERVENTION		
Intervention Practice Week 1	Play as per self-identified schedule	Participant	~30 * 3-5 sessions
Intervention 1st Visit	AROM test-1<br>Play session - same as home practice, but with researcher<br>- Video recording<br>- Electro-goniometer<br>- Game calibration (if required)<br>AROM test-2	Researcher	60 min

	Check-in conversation		
Intervention Practice Week 2	Play as per self-identified schedule	Participant	~30 * 3-5 sessions
Intervention 2nd Visit	AROM test-3<br>Play session - same as home practice, but with researcher<br>- Video recording<br>- Electro-goniometer<br>- Game calibration (if required)<br>AROM test-4<br>Check-in conversation	Researcher	60 min
Intervention Practice Week 3	Play as per self-identified schedule	Participant	~30 * 3-5 sessions
Intervention 3rd Visit	AROM test-5<br>Play session - same as home practice, but with researcher<br>- Video recording<br>- Electro-goniometer<br>- Game calibration (if required)<br>AROM test-6<br>Check-in conversation	Researcher	60 min
Intervention Practice Week 4	Play as per self-identified schedule	Participant	~30 * 3-5 sessions
Intervention 4th Visit	AROM test-7<br>Play session - same as home practice, but with researcher<br>- Video recording<br>- Electro-goniometer<br>- Game calibration (if required)<br>AROM test-8<br>Check-in conversation	Researcher	60 min
	Post-Intervention		
Post-Intervention 1st visit<br><br>< 1-week past 4th intervention visit	Assessments<br>- AHA<br>- Box and Blocks<br>Exit conversation (with person other than original)<br>- Reflection of COPM goals	Therapist<br><br>Researcher	60-90

10.2 Appendix 2. Guiding the initial dialogue

During the initial dialogue, the COPM data will be obtained. COPM is a semi-structured interview tool used to identify goal areas, generally related to: personal care, productivity, and leisure [52]. COPM goal areas are framed in the context of what the intervention addresses. The COPM goals will address reaching and grasping functions since the intervention facilitates practice of wrist extensions and thumb-finger pinching at specific times (reach/grasp elements). During the intervention, participant do many repetitions of these actions and at specific times and frequencies to successfully navigate the game. Through this practice, participants' goals, related to reaching and grasping, will be addressed.

COPM goals can be developed during Solution Focused Coaching conversations. A framework for this has been developed at Holland Bloorview Kids Rehabilitation Hospital Toronto, Canada (unpublished by Sarah Keenan, in her role leading the SFC coaching training at Holland Bloorview). Team members in the initial conversation will use the following resource to guide the conversation:

Introduction - framing the conversation and showing the purpose of the study:

1. Therapist: We have a video game that you control by moving your hand. In the game, you lift your hand or pinch your fingers together to get your character to avoid the obstacles. It is all set to music, and there are hidden awards and characters to find as you play. *[show/try example, and answer questions]*
2. Therapist: The reason we made this game is so that there was a fun way to get better at using your non-dominant hand. When you play this game, you are actually doing parts of movements that you might already be doing every day for lots of different things like: removing a lid from a bottle, opening a back-pack, unlocking a door, using your phone. …. is there anything else like these things you do often? *[looking to know that they understand that it is reach and grasp related tasks…]*

Example of developing COPM goal areas through SFC conversation:

1. Therapist: Given what you do in the game and why we made it, and the kinds of goals we have talked about, what are your **best hopes** from your participation in the study? [53]
2. So, suppose **tomorrow** your best hopes had been realized, what would be different? [53]
3. Immediately ask **scaling questions** → yields COPM Performance and Satisfaction
    a. On a **scale** of 10 to 1, where 10 is you are [goal specific], and 1 is the opposite, where are you now? …And how satisfied are you with that 4, on a scale of 1 to 10?
    b. You were a 4 on that scale – how did you manage to get to a 4, and not lower? [54]
    c. Suppose you were half a point higher on that scale. What would you be doing? [54]
4. Continue with SFC, adding to/refining goals as you are listening.
5. *At the end of the conversation*: goal confirmation as part of summary feedback
    a. At the end of our month of playing together, what will have made this worthwhile? → check in with whether that's what they want, does it inform goals?

Developing an action plan:

1. Imagine you can look into the future, but on which days in the coming week could you see yourself playing?
2. When I see you next time, and I ask you on which days you played what will you tell me?

3. Would it be helpful if we looked at your schedule this coming week and find times when it works well for you to play? We have a calendar here and you can it in as you like? [16]

The following table details the specific data elements to be captured during the initial conversation and when participants can expect to hear these responses form the participant.

10.3 Data collection link to questions

SFC-peds	Questions	Data Collection
**Introduction - framing the conversation and showing the purpose of the study:**		
Setting the stage	1. Therapist: We have a video game that you control by moving your hand. In the game, you lift your hand or pinch your fingers together to get your character to avoid the obstacles. It is all set to music, and there are hidden awards and characters to find as you play. *[show/try example, and answer questions]*	Informed consent
Setting the stage	2. Therapist: The reason we made this game is so that there was a fun way to get better at using your non-dominant hand. When you play this game, you are actually doing parts of movements that you might already be doing every day for lots of different things like: removing a lid from a bottle, opening a back-pack, unlocking a door, using your phone. …. is there anything else like these things you do often? *[looking to know that they understand that it is reach and grasp related tasks…]*	Inform goals
**Addressing COPM goals:**		
Envisioning a Preferred Future	1. Therapist: Given what you do in the game and why we made it, and the kinds of goals we have talked about, what are your **best hopes** from your participation in the study?	Inform goals
Envisioning a Preferred Future	2. So, suppose **tomorrow** your best hopes had been realized, what would be different?	Inform goals
Goal Discovery	3. Immediately ask **scaling questions** → yields COPM Performance and Satisfaction	COPM - Performance
Goal Discovery/ Strategy Creation	a. On a **scale** of 10 to 1, where 10 is you are [goal specific], and 1 is the opposite, where are you now? …And how satisfied are you with that 4, on a scale of 1 to 10?	COPM - Satisfaction
Goal Discovery/ Strategy Creation	b. You were a 4 on that scale – how did you manage to get to a 4, and not lower?	Identifying past success
Goal Discovery/ Strategy Creation	c. Suppose you were half a point higher on that scale. What would you be doing?	Identifying possible next steps
Goal Discovery/ Strategy Creation	4. Continue with SFC, adding to/refining goals as you are listening.	Inform goals
Plan Confirmation	5. *At the end of the conversation*: goal confirmation as part of summary feedback	Inform goals

Action and Reflection Cycle	a. At the end of our month of playing together, what will have made this worthwhile? → check in with whether that's what they want, does it inform goals?	Inform goals
**Developing an action plan:**		
Strategy Creation/Plan Confirmation/ Action	1. Imagine you can look into the future, on which days in the coming week could you see yourself playing?	Expected training schedule
Strategy Creation/Plan Confirmation/ Action	2. When I see you next time, and I ask you on which days you played what will you tell me?	Expected training schedule
Strategy Creation/Plan Confirmation/ Action	3. Would it be helpful if we looked at your schedule this coming week and find times when it works well for you to play? We have a calendar here and you can fill it in as you like? (see Appendix 4b)	Expected training schedule

10.4 Appendix 3: Check-in conversation resource

The participant will meet with the coach/researcher once per week to play the game and perform functional tests (AROM). During this time, the coach will have a short 'check-in' conversation to determine if the participant is satisfied with their progress, gauge their motivation, and help them modify the action plan if necessary. This short conversation will follow the commonly used solution-focused method known as EARS (elicit, amplify, reinforce, and start over) [55]. This conversation guide is used to help clients reflect on and build from past positive experiences.

*Solution-Focused Coaching: **EARS** [55] Check-in conversation pattern*

When you think about your practice goals, how do feel you have done this past week, on a scale of 1 to 10, with 10 being what you want, and 1 being the opposite, where are you on the scale right now?

1. **E**licit the Exception
    a. What has worked *to help make sure you practice*?
    b. What is better since I last saw you?

2. **A**mplify the Exception
    a. What did you do *to help make sure you practice*? How did you do that?
    b. How have you managed *to make sure you practiced this past week*?
    c. What might others say you've done to help *make sure you practiced this past week*?
    d. What was helpful for you along the way? What else…?
    e. What is different for you as a result of this? What else…?

3. **R**einforce the Successes and Strengths
    a. What can help you maintain these improvements *in how you have been practicing*?
    b. What have you learned as a result?

4. **S**tart Over
    a. Given what you've been learning, what's needed now *to make sure you meet your practice goal next week*?

10.5 Appendix 4a: Conversation additional resources

The following list will be provided to team members completing the conversations with participants. The resource consists of Solution-Focused questions [16,54].

Additional Questions

1. Coaching Agreement/Contracting
    a. What needs to happen in our conversation today, so that it will turn out to be really useful for you?
    b. Suppose this turns out to be useful for you, what will you do differently then?
    c. OR: What are your best hopes from our conversation?
    d. Confirm the contract: So, if in this conversation we were able to … would that be helpful?

2. Exploring the Preferred Future

a. Supposing you woke up tomorrow and your hopes about this have somehow been realized. What's the first thing you might notice yourself doing differently?
    i. Who else might notice this has happened?
    ii. What will they notice is different?
    iii. What else might you or others notice?

3. Exploring Precursors/Exceptions
    a. When was a most recent example that a small piece of what you are hoping for is already happening?
    b. What are other examples?
    c. How did you help that to happen?
    d. When you think of when things are as you hope, on a scale of 1 to 10, with 10 being what you want, and 1 being the opposite, where are you on the scale right now?
        i. What do you do that helps you be there and not a lower number?

4. Progress clues
    a. Suppose you were one point higher on the scale, how will you notice that?
        i. What will you be doing differently that you're not doing now?
        ii. What might other people notice?
    b. I am impressed by…
    c. After this conversation today, what will be a small sign that you are already making progress?
    d. What can give you strength and confidence along the way?

Researches will also be able to use the following checklist to ensure that all of the required data elements have been captured during the discussion with participants.

Checklist: Data elements and points of interest

COPM

1) Hand function specific goals identified in line with intervention opportunities
    a) Performance rating identified for each goal
    b) Satisfaction rating identified for each goal
    c) Importance rating identified for each goal

Action plan

1) Participant has identified their intended practice
    a) Template schedule completed
    b) Number of days to play next week ____
    c) Number of minutes to play each time ____

Motivation

1) Participant has identified something they do well related to activity/ function
2) Participant has identified something they would like to improve

3) Participant has identified their goal for volunteering for the intervention
    i) On weekly follow-up, the participant has discussed their motivation
        (a) from the past week
        (b) for the coming week

10.6 Appendix 4b: Conversation additional resources (Figure 1) – Example action plan

June 5 - 11, 2017

	Jun 5 Mon	Jun 6 Tue	Jun 7 Wed	Jun 8 Thu	Jun 9 Fri	Jun 10 Sat	Jun 11 Sun
7:00							
8:00	Play game		Play game				
9:00	School	School	School	School	School		
10:00							Play game
11:00							
12:00							
13:00							
14:00						At Grandmas	
15:00							
16:00							
17:00		At friends		Therapy			
18:00	Rehersal			Play game			
19:00						Play game	
20:00							
21:00							
**Notes**	Can play for a while before school, after breakfast					Can show grandma the game I am playing	

An example template schedule built by the participant based on conversation with the coach. They identified their regular routine and identified with the Play game block where they could see themselves playing in the coming week.

10.7 Appendix 5: Discussion resources

These questions will be used to inform the Final discussion to gain subjective impression of the game. The researcher can draw from any of these questions and past experiences with the participant to gain an understanding of their subjective impressions of the game.

Participant ID#: _______________

Game Feedback Questionnaire

This questionnaire is based on validated questionnaires for measuring usability (SUS[56]) and enjoyment (Flow short[57]) and a custom questionnaire used in a previous study investigating ICP therapy games with this population[58].

USABILITY: How much do you agree to each of the following statements?	not at all		partly		very much
Learning to play the game was easy[56,58]					
The goal of the game was clear and simple[56,58]					
The controls in the game were very responsive[58]					

FUN (challenge and immersion): How much do you agree to each of the following statements?	not at all		partly		very much
I felt just the right amount of challenge[57]					
My score/success in the game was fair[58]					
The game was fun[58]					
I was totally absorbed in what I was doing in the game[57]					
The difficulty of the game felt appropriate for me[57]					
I would enjoy playing this game frequently[56]					

Open questions to facilitate conversations about subjective impressions of the game:

1. What parts of the game did you find most fun?
2. What helped to make the game easier to play?
3. What helped to make the game harder to play?
4. Was there anything that you used when playing the game to help you succeed?
5. What was the most important part of the game for you?
6. Did the score change how you played the game?
7. Did the characters change how you played the game?
8. What was your strategy to avoid the obstacles?
9. Did you feel like you were in control of the game?
10. Did anything hold you back from playing the way you wanted?
11. Name the game you have played that is most similar to this game.
12. What was your favorite moment or interaction?
13. What was your least favorite moment or interaction?
14. What was your least favorite moment or interaction?
15. If you could change any aspect of the game or your experience, what would it be? Unlimited budget and time.

This Self-Reported Experiences of Activity Settings (SEAS) questionnaire will be used here to evaluate the participant's interaction, engagement, and control while playing the ICP game at home. SEAS is a 22-item questionnaire that takes less than 15 minutes to complete. It offers a way to gain situation-specific understanding of youth with disabilities participation in leisure activities. The questionnaire has good internal consistency (Cronbach's alpha from 0.71 to 0.88). The complete list of questions can be seen below and the formatted questionnaire in the associated documents.

SEAS question list

Number	Question	Used in this study
1	"I learned a new skill"	✓
2	"I became better at something"	✓
3	"I was challenged"	✓
4	"I tried something new"	✓
5	"I grew or changed"	✓
6	"I discovered things about myself"	✓
7	"I was having fun"	✓
8	"I felt in a good mood"	✓
9	"I was interested"	✓
10	"I felt excited"	✓
11	"I got along with others"	×
12	"I belonged (i.e. I was part of the group)"	×
13	"I was supported and encouraged by others"	×
14	"I was valued by others"	×
15	"I talked about my thoughts and feelings"	×
16	"I shared ideas about things important to me"	×
17	"I had good conversations with others"	×
18	"I shared something special"	✓
19	"I could choose what to do for the most part"	✓
20	"I was in control"	✓
21	"I had a say in things"	✓
22	"I was free of pressure"	✓

10.8 Appendix 6: Template data collection forms

Demographics Data Collection Form

REB#	
Completed by:	
Date collected:	
Participant ID:	
Birth year and month:	
Gender:	Complete or leave as desired: ____
Diagnosis	

ROM Data Collection Form

REB#	
Completed by:	
Date collected:	
Participant ID:	
AROM Measure 1	
PROM	
Grip Strength	
AROM Measure 2	
PROM	
Grip Strength	

10.9 Appendix 7: Post-intervention phone interview resource

Sample questions of intervention use, perceived opportunities and risks based on previous work (Chaudron et al 2015)

1. How did MyoDashy fit into your family's routine? (when/how much/with who did your child play)
2. How independent was your child in his/her use of MyoDashy?
3. Do you think any aspects of MyoDashy were particularly "positive" or "negative" for your children? - Which ones? Why?
4. Are you worried in any ways about your children`s experiences with MyoDashy (e.g. children spending too much time)? Why?
5. How important do you think technologies like MyoDashy are for your child? How important are new technologies like MyoDashy for you? And for family life?
6. Do you think that your child's use of MyoDashy affected in any way (positive and/or negative) your family life? (E.g. family interaction is decreasing or increasing)
7. Do you feel that parenthood is helped or influenced or affected in any ways by the use of MyoDashy at home? How?
8. Do you feel that your child benefitted from using MyoDashy? Why?
9. Do you have any worries or concerns about your child using MyoDashy? Or about the use of MyoDashy at home? If you do, what do you do about it?
10. Has anyone in your family experienced a positive/exciting/enlightening situation using MyoDashy?
11. What happened? What did you/your child do about it?
12. Has anyone in your family experienced a difficult/unpleasant situation with MyoDashy? What happened? What did you/your child do about it?
13. Anything else to share? Unusual/unexpected/surprising

Chaudron, S. Young Children (0-8) and Digital Technology: A qualitative exploratory study across seven countries. Technical report by the Joint Research Centre of the European Commission (2015). doi:10.2788/00749
