# Supplementary material for: A biofeedback-enhanced therapeutic exercise video game intervention for young people with cerebral palsy: A randomized single-case experimental design feasibility study
Source: PLoS One. 2020 Jun 22;15(6):e0234767. doi: 10.1371/journal.pone.0234767 (PMC7307764; doi:10.1371/journal.pone.0234767)
Supplement: S7 File — (PDF) [file pone.0234767.s007.pdf]

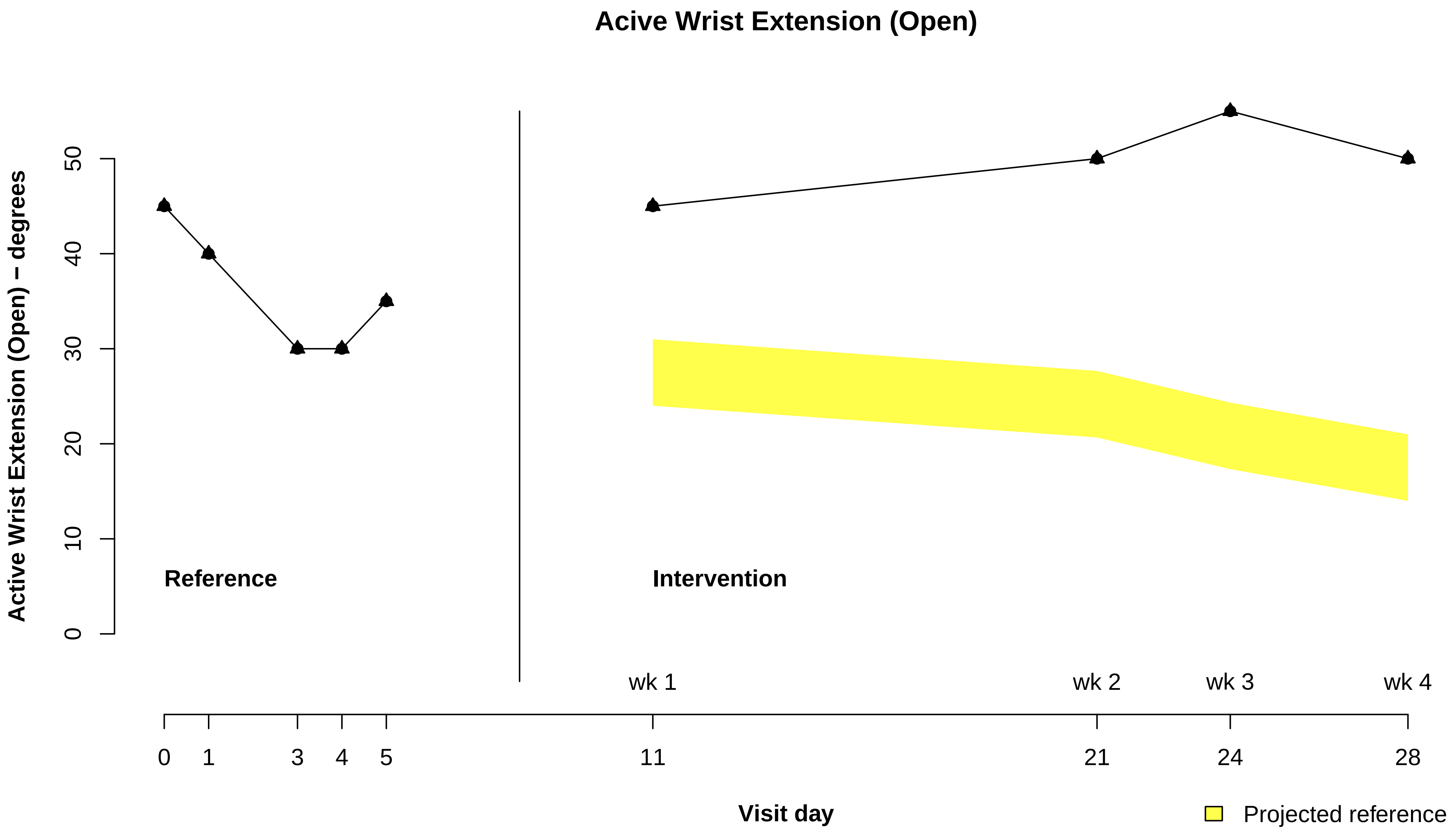

A

Reference phase trend: minimal (−2 deg/visit)
Reference phase stability: stable (80% in envelope)
Level change estimate: large
Intervention phase 19 deg higher than Reference phase
Average of 4 degree increase across Intervention visits
0% overlap between phases (PAND)

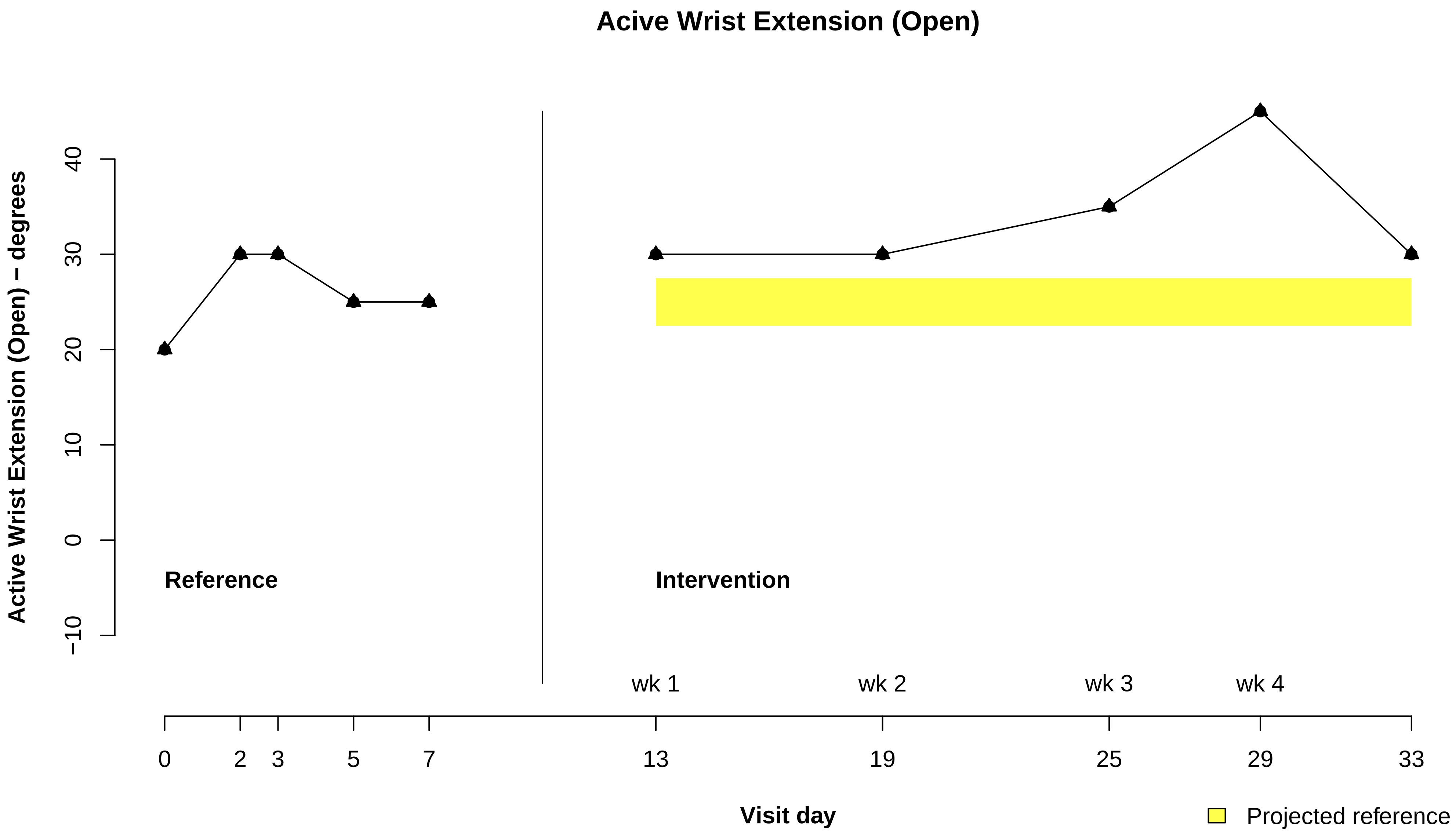

B
Reference phase trend: minimal (1 deg/visit)
Reference phase stability: stable (100% in envelope)
Level change estimate: minimal
Intervention phase 4 deg higher than Reference phase
Average of −1 degree decrease across Intervention visits
30% overlap between phases (PAND)

Active Wrist Extension (Open)

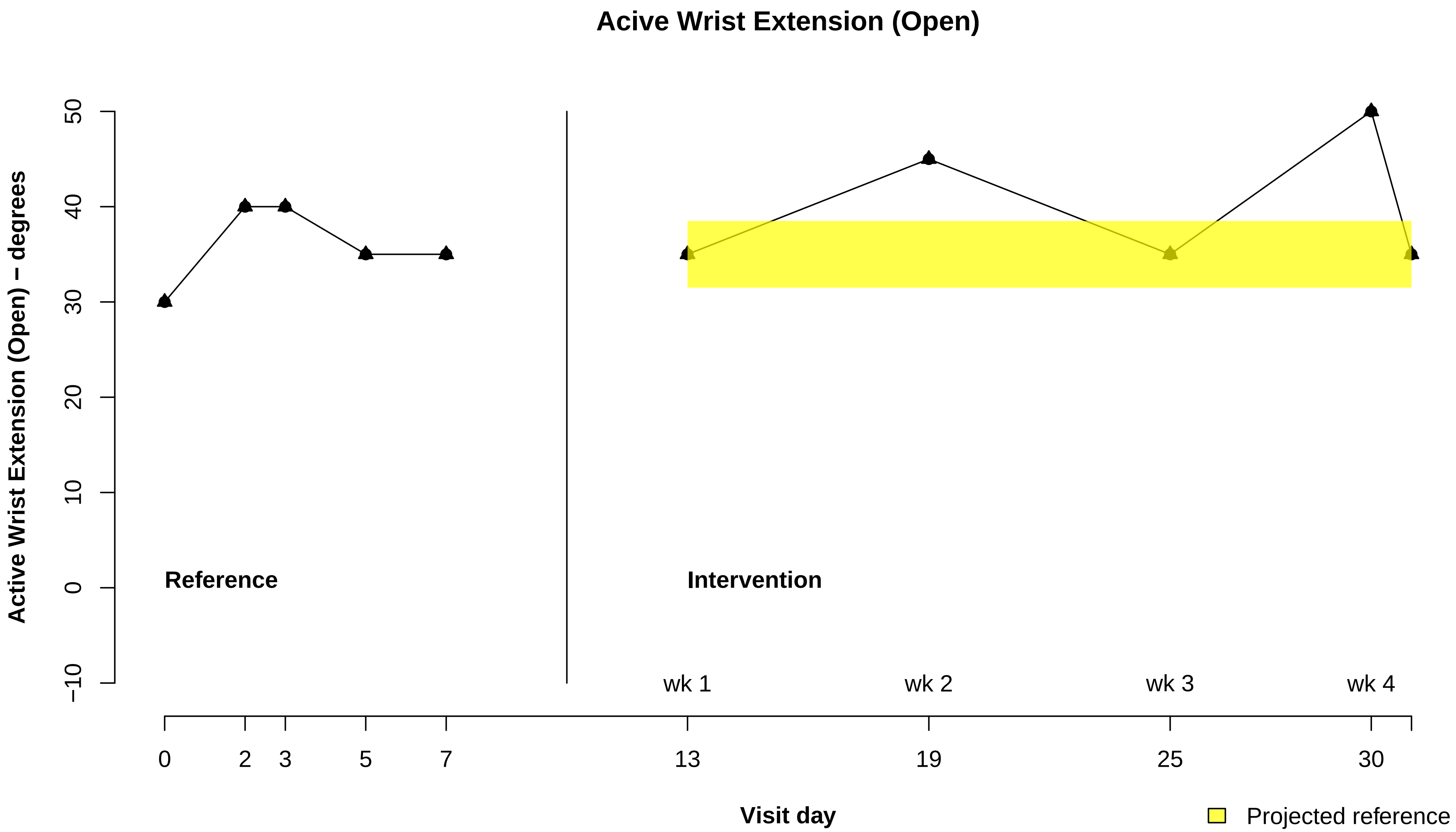

C
Reference phase trend: minimal (1 deg/visit)
Reference phase stability: stable (100% in envelope)
Level change estimate: minimal
Intervention phase 0 deg higher than Reference phase
Average of −1 degree decrease across Intervention visits
40% overlap between phases (PAND)

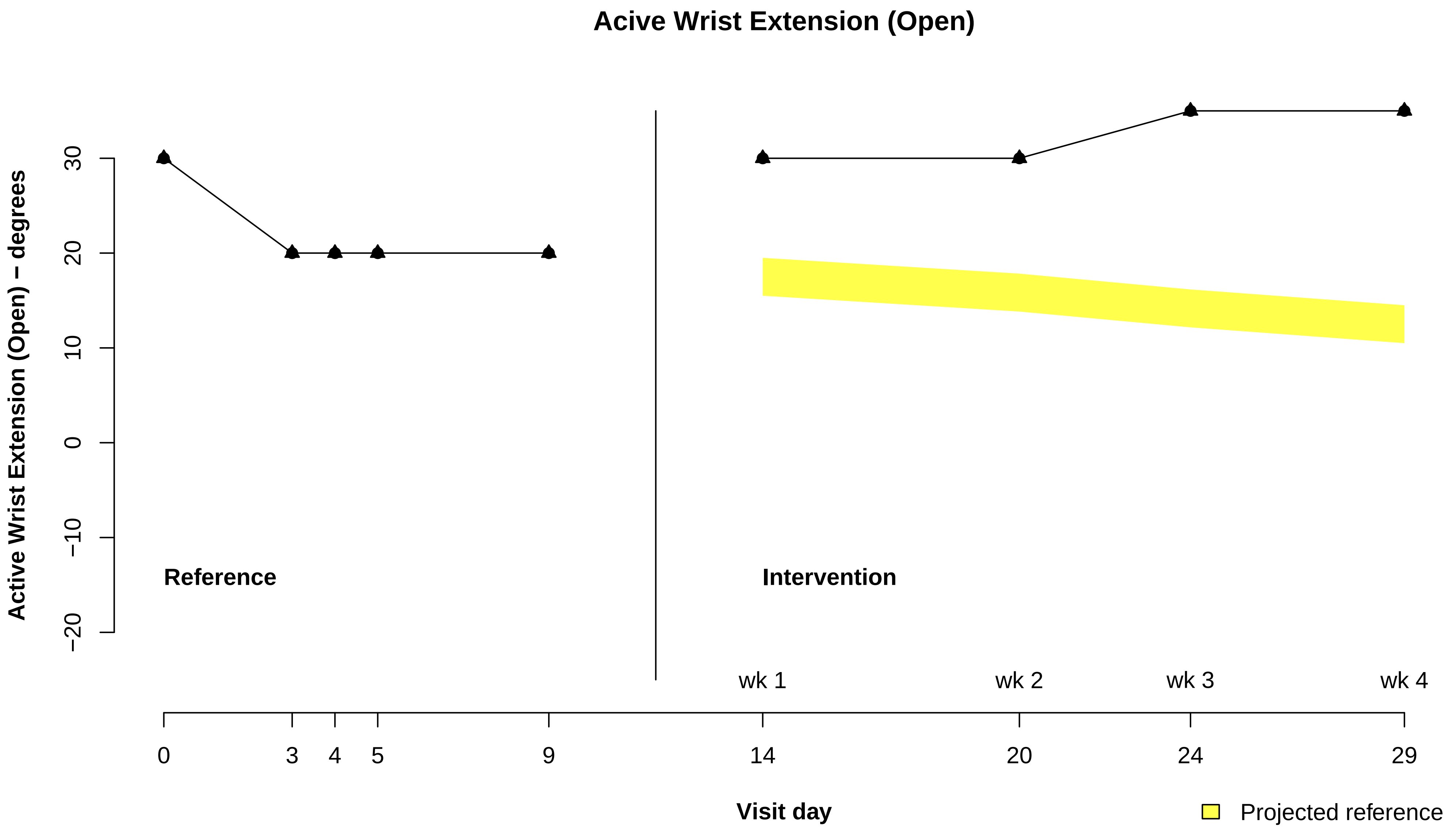

D
Reference phase trend: minimal (−2 deg/visit)
Reference phase stability: highly variable (0% in envelope)
Level change estimate: large
Intervention phase 16 deg higher than Reference phase
Average of 4 degree increase across Intervention visits
0% overlap between phases (PAND)

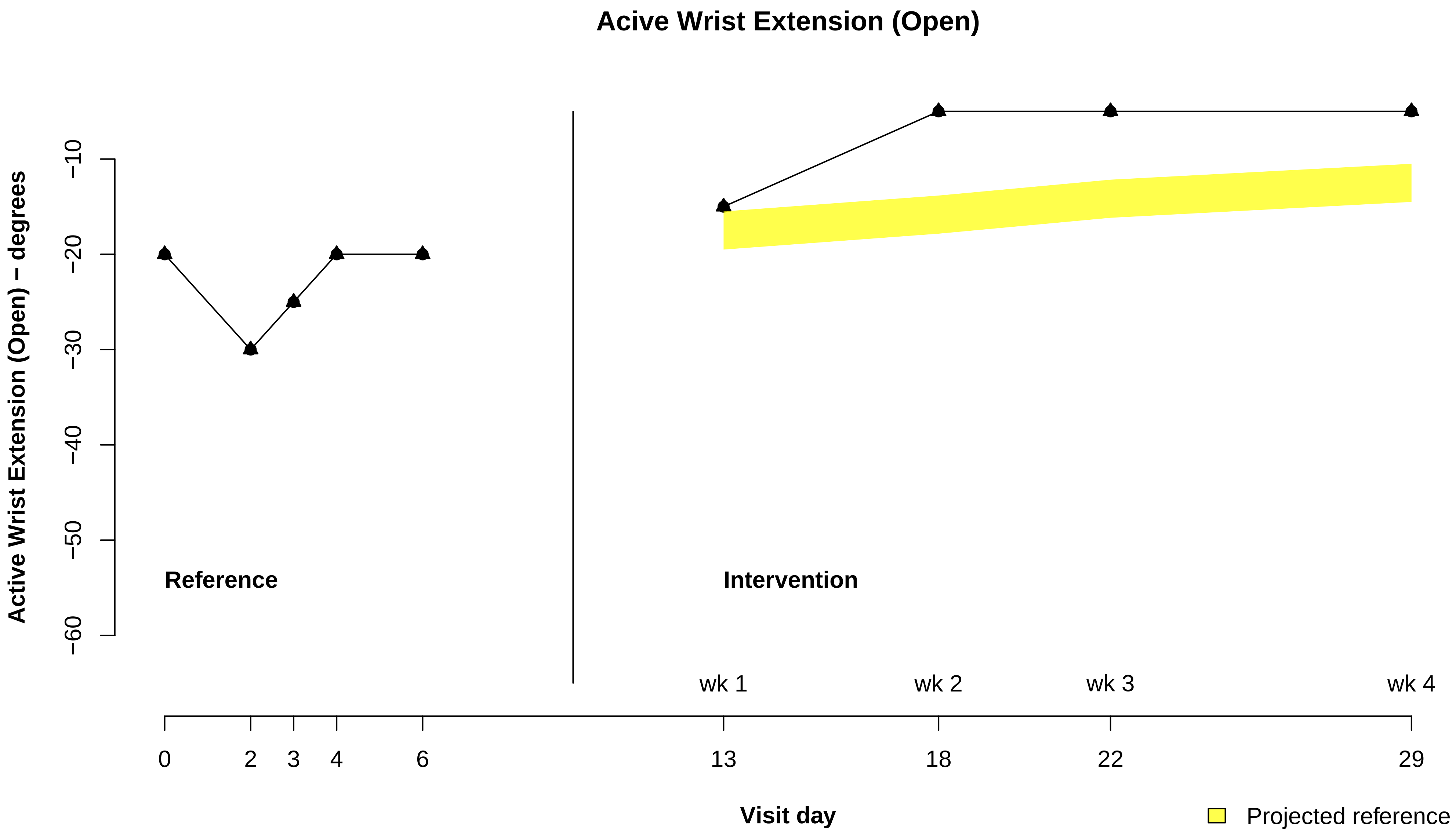

E
Reference phase trend: minimal (0 deg/visit)
Reference phase stability: somewhat variable (60% in envelope)
Level change estimate: moderate
Intervention phase 10 deg higher than Reference phase
Average of 3 degree increase across Intervention visits
0% overlap between phases (PAND)

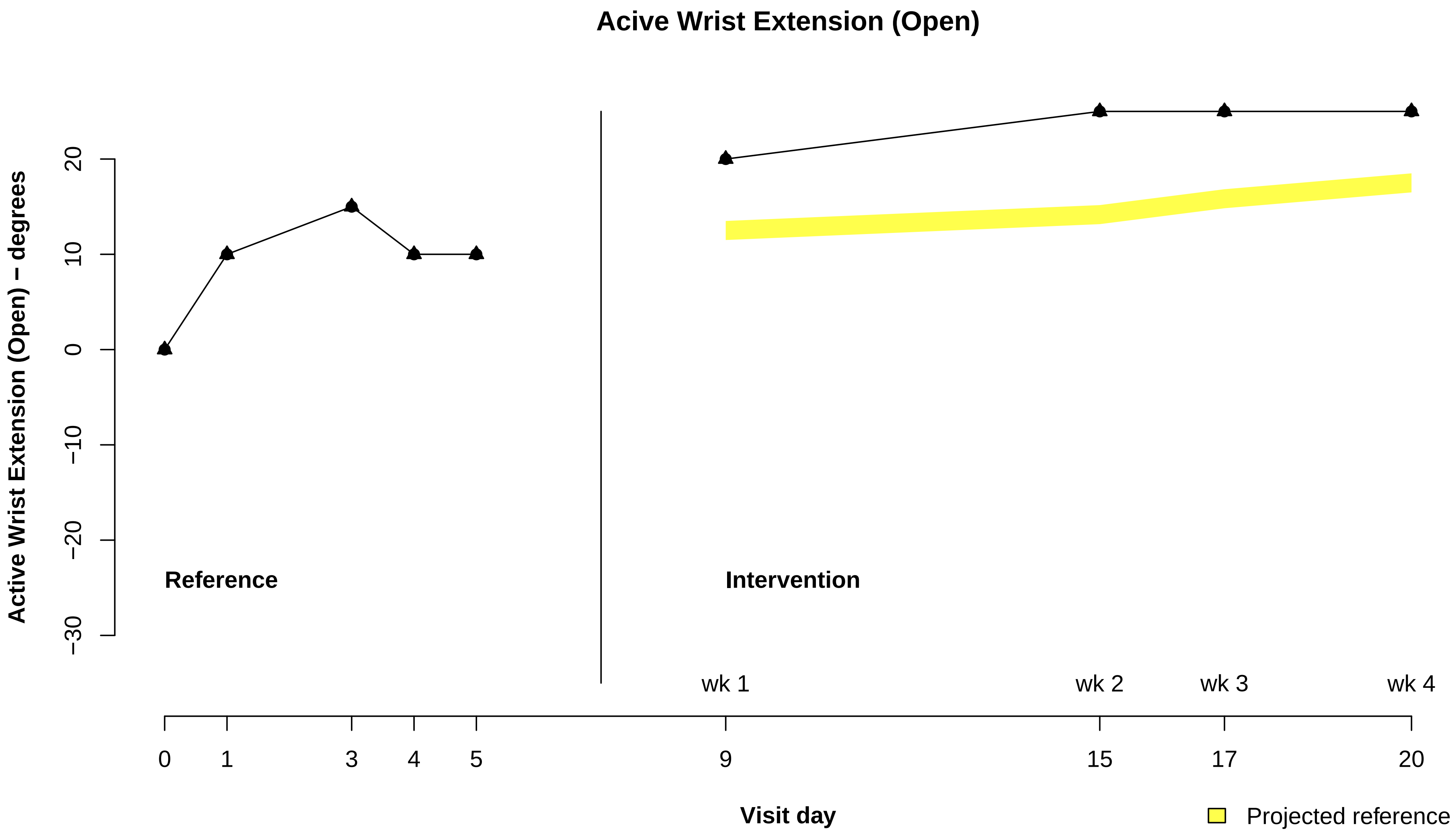

F
Reference phase trend: minimal (2 deg/visit)
Reference phase stability: highly variable (0% in envelope)
Level change estimate: minimal
Intervention phase 5 deg higher than Reference phase
Average of −1 degree decrease across Intervention visits
22% overlap between phases (PAND)

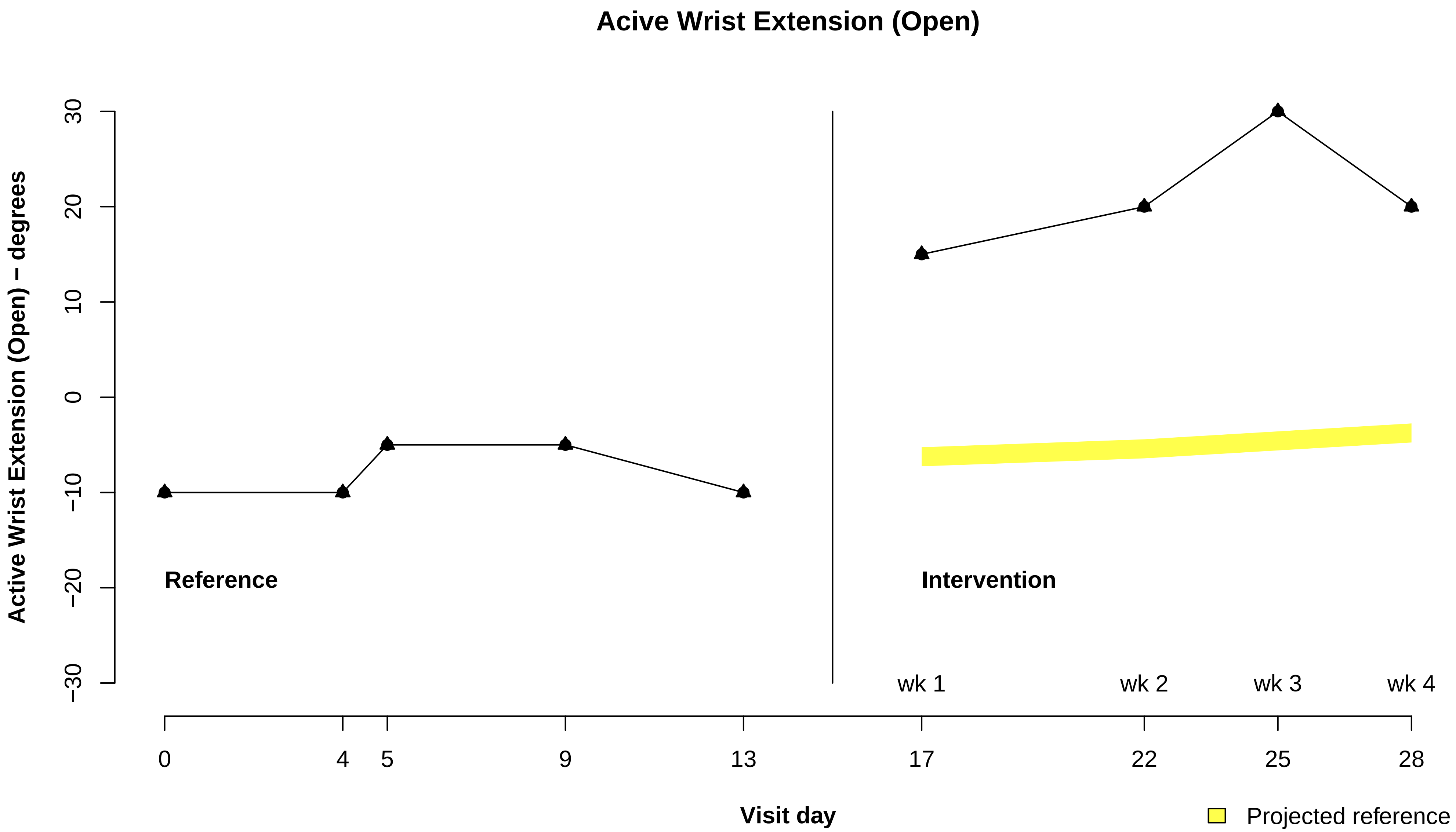

G
Reference phase trend: minimal (0 deg/visit)
Reference phase stability: stable (100% in envelope)
Level change estimate: large
Intervention phase 27 deg higher than Reference phase
Average of 2 degree increase across Intervention visits
0% overlap between phases (PAND)

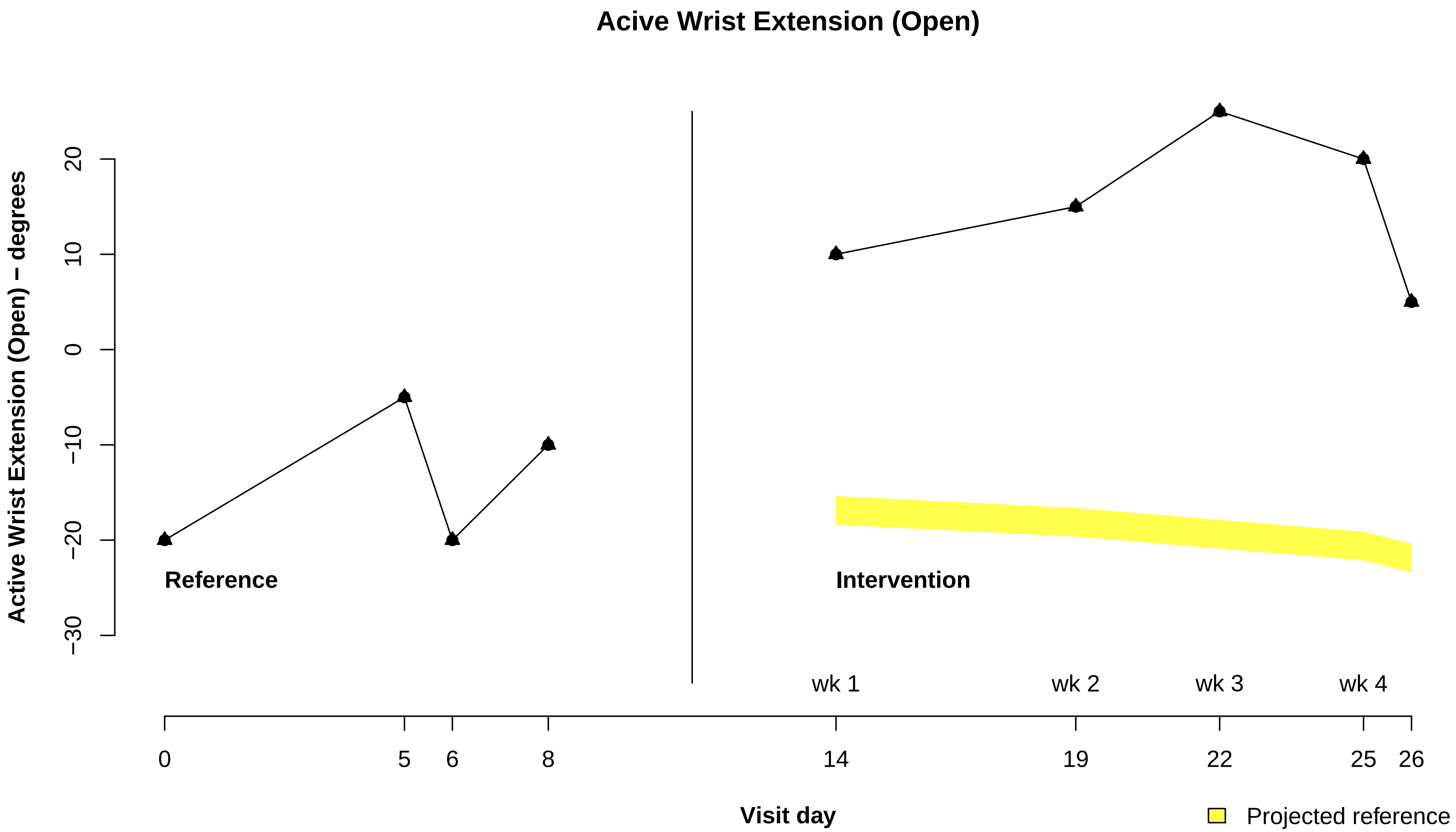

H
Reference phase trend: minimal (3 deg/visit)
Reference phase stability: stable (100% in envelope)
Level change estimate: large
Intervention phase 23 deg higher than Reference phase
Average of −5 degree decrease across Intervention visits
11% overlap between phases (PAND)

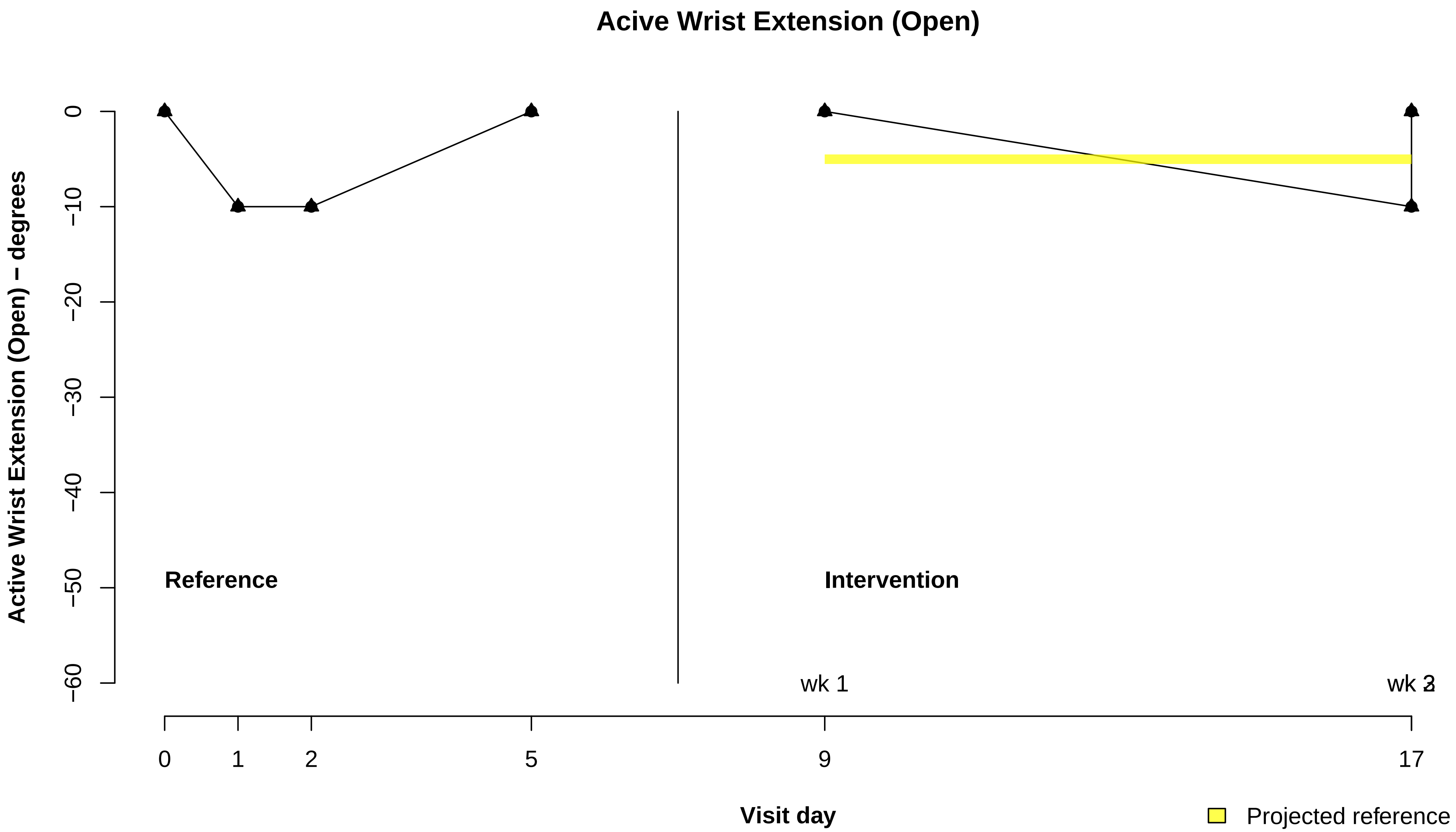

I
Reference phase trend: minimal (0 deg/visit)
Reference phase stability: stable (100% in envelope)
Level change estimate: minimal
Intervention phase 2 deg higher than Reference phase
Average of 0 degree increase across Intervention visits
43% overlap between phases (PAND)

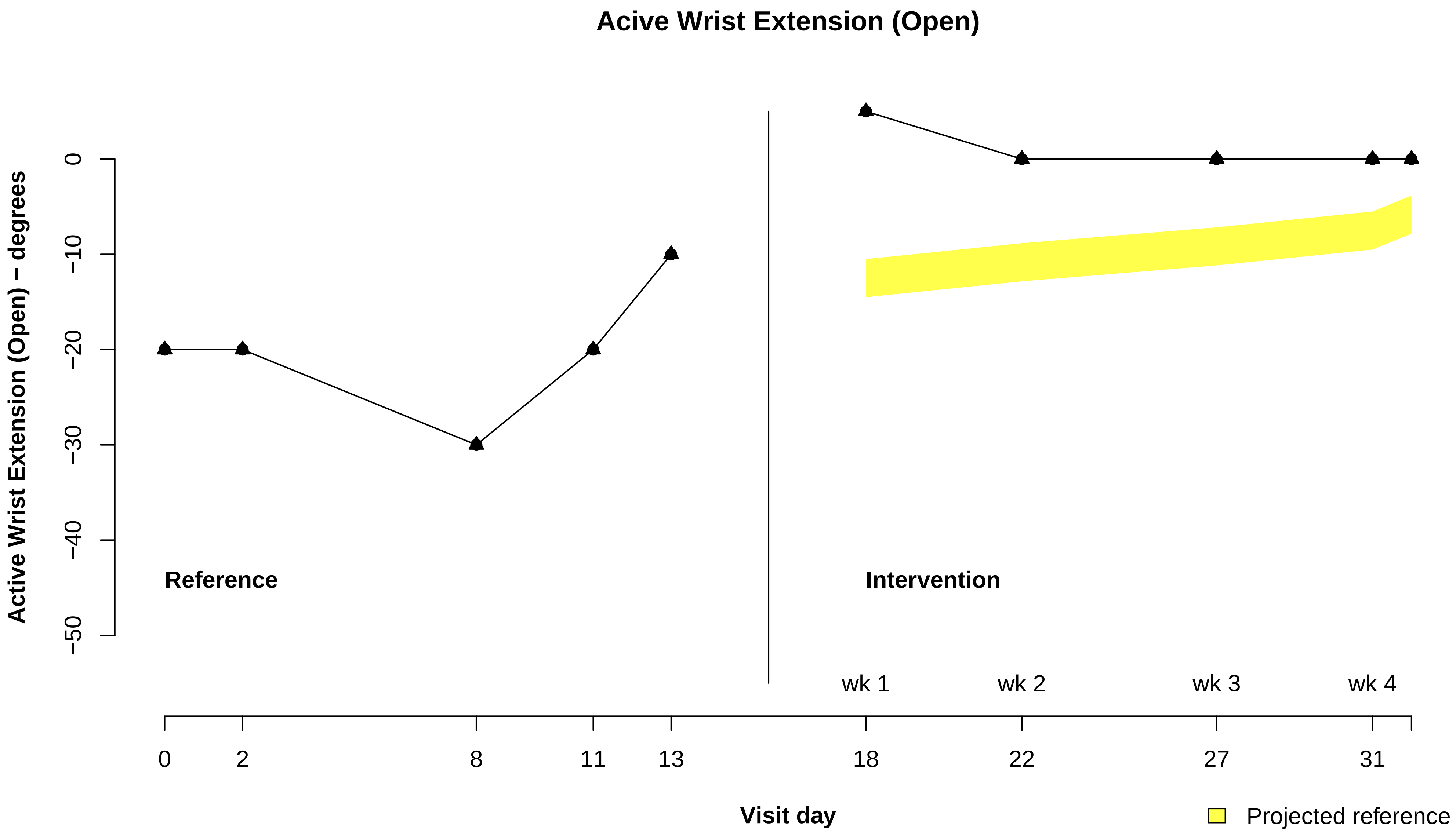

J
Reference phase trend: minimal (2 deg/visit)
Reference phase stability: highly variable (0% in envelope)
Level change estimate: large
Intervention phase 16 deg higher than Reference phase
Average of −4 degree decrease across Intervention visits
20% overlap between phases (PAND)

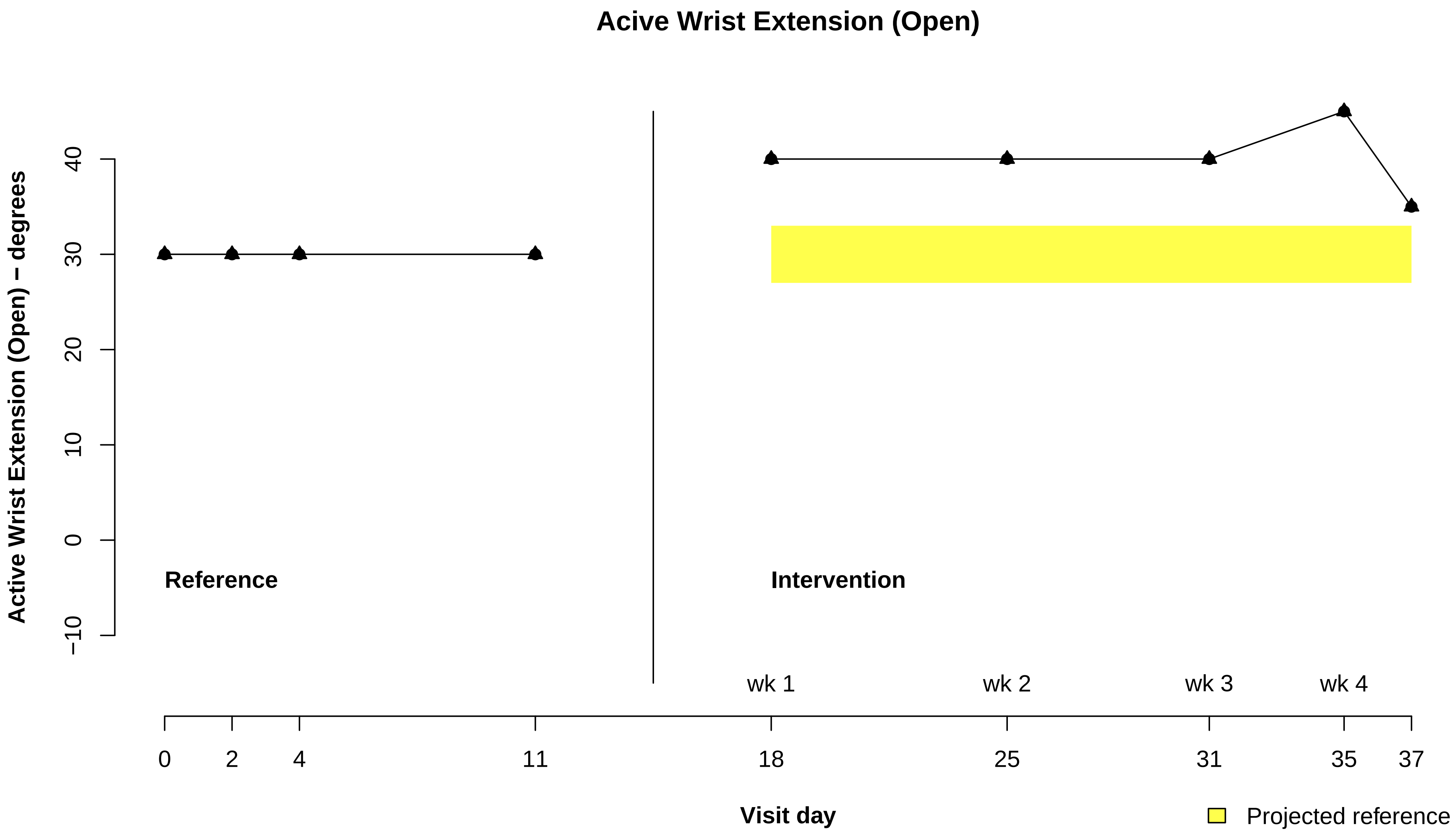

K
Reference phase trend: minimal (0 deg/visit)
Reference phase stability: stable (100% in envelope)
Level change estimate: moderate
Intervention phase 12 deg higher than Reference phase
Average of −1 degree decrease across Intervention visits
0% overlap between phases (PAND)

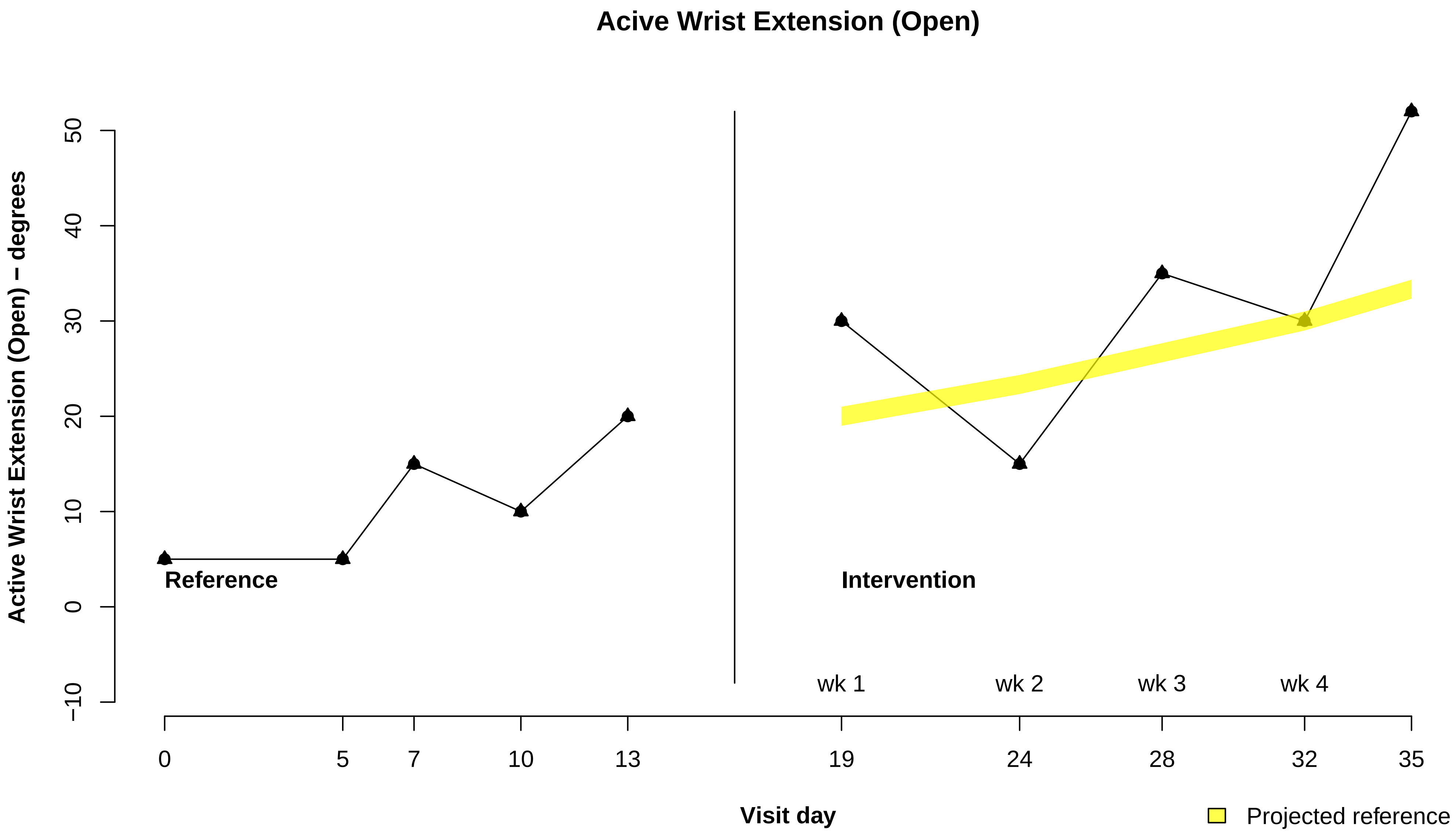

L
Reference phase trend: minimal (4 deg/visit)
Reference phase stability: somewhat variable (60% in envelope)
Level change estimate: minimal
Intervention phase −1 deg lower than Reference phase
Average of 2 degree increase across Intervention visits
20% overlap between phases (PAND)

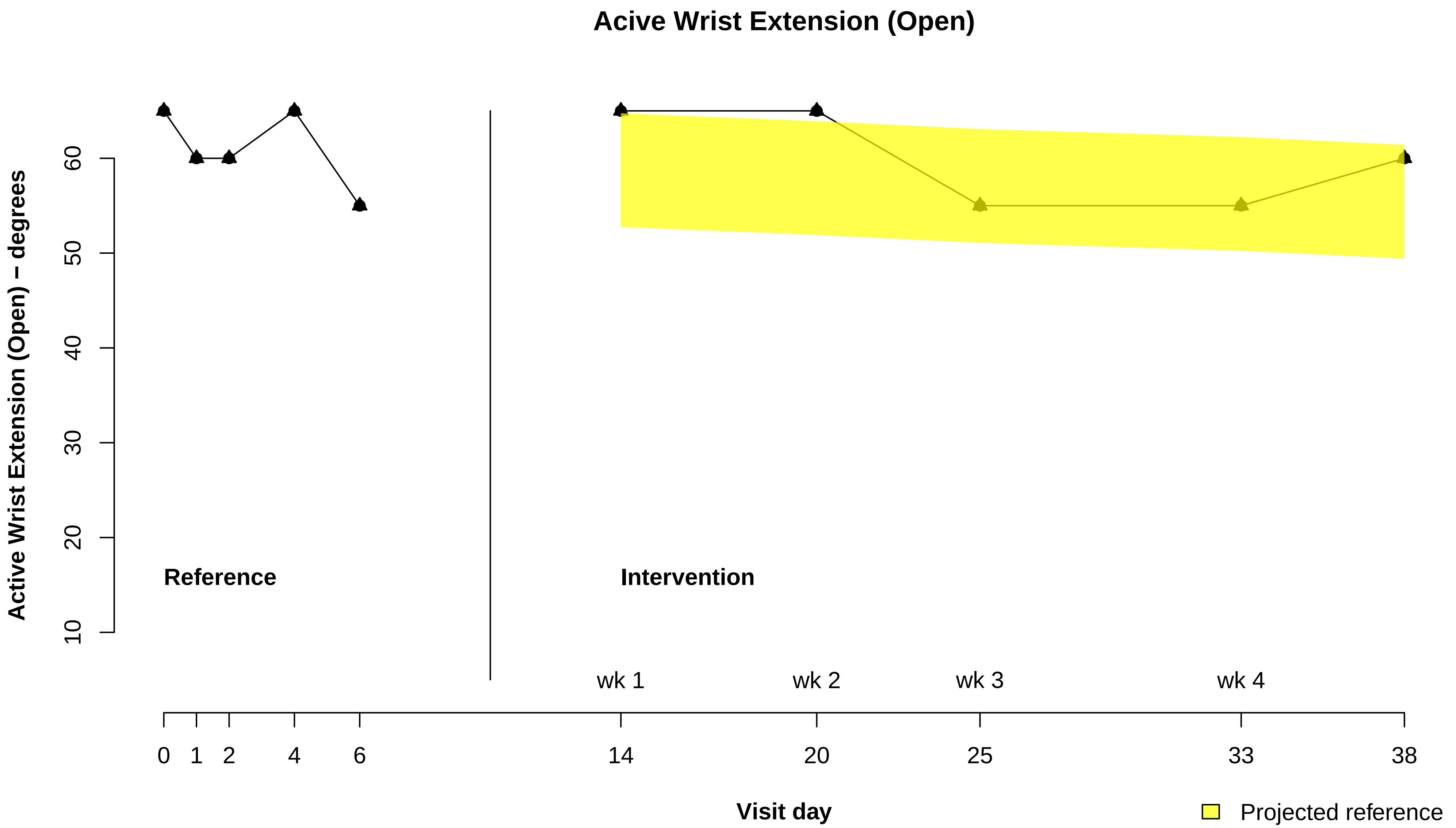

M
Reference phase trend: minimal (−2 deg/visit)
Reference phase stability: stable (100% in envelope)
Level change estimate: moderate
Intervention phase 9 deg higher than Reference phase
Average of 1 degree increase across Intervention visits
10% overlap between phases (PAND)

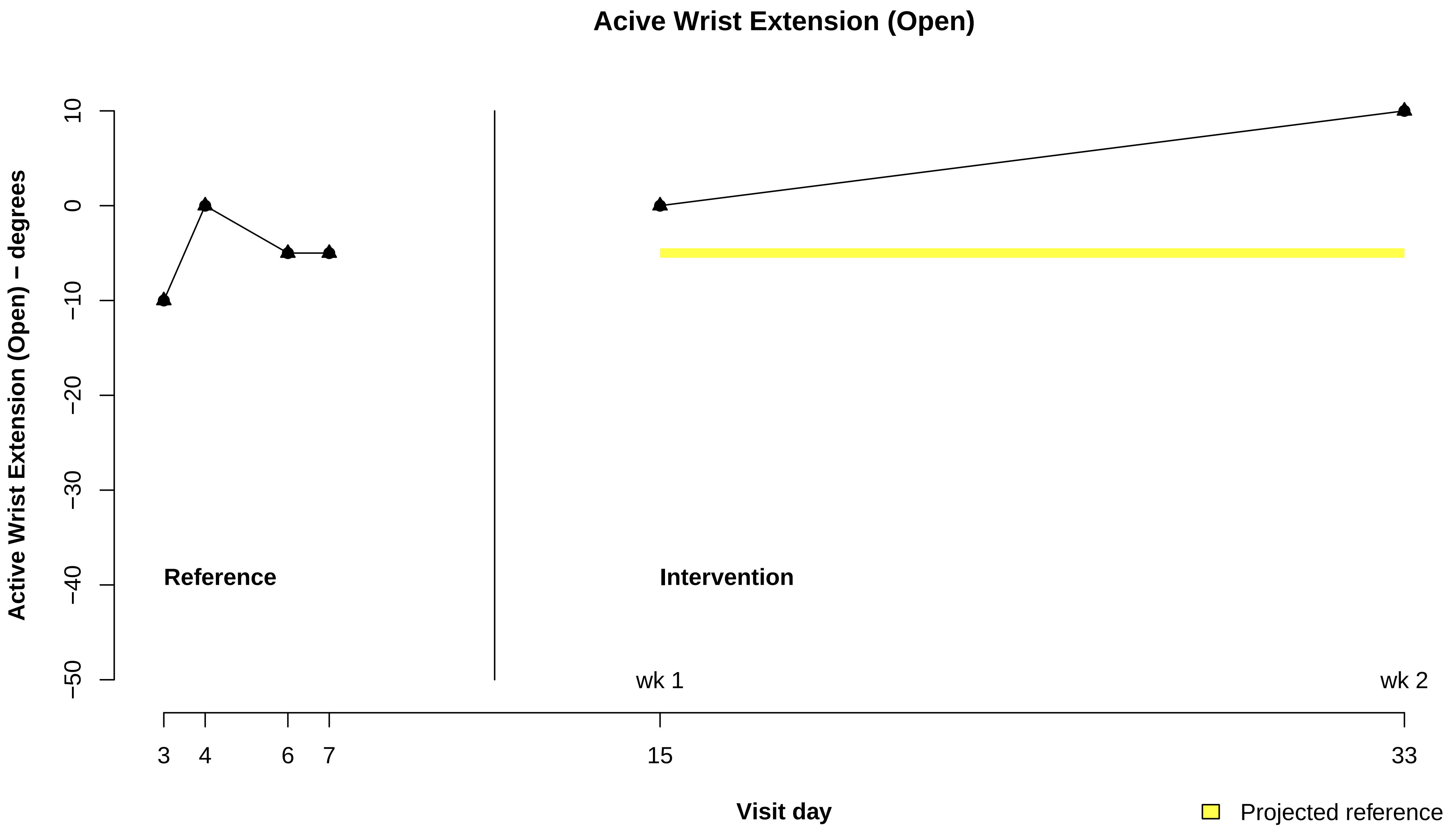

N

Reference phase trend: minimal (2 deg/visit)

Reference phase stability: highly variable (50% in envelope)

Level change estimate: minimal

Intervention phase 1 deg higher than Reference phase

Average of 8 degree increase across Intervention visits

17% overlap between phases (PAND)

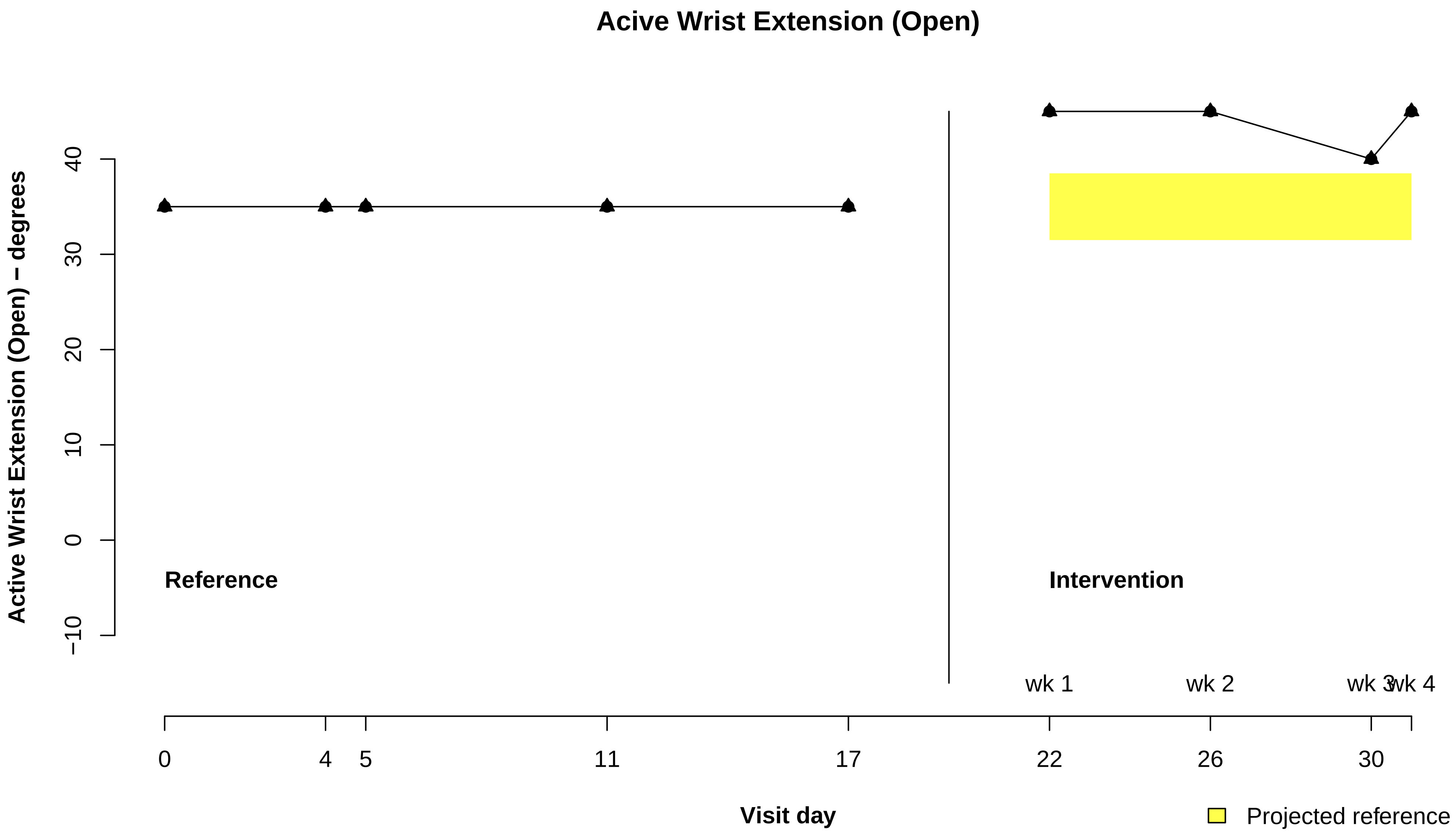

O
Reference phase trend: minimal (0 deg/visit)
Reference phase stability: stable (100% in envelope)
Level change estimate: moderate
Intervention phase 9 deg higher than Reference phase
Average of 0 degree increase across Intervention visits
0% overlap between phases (PAND)

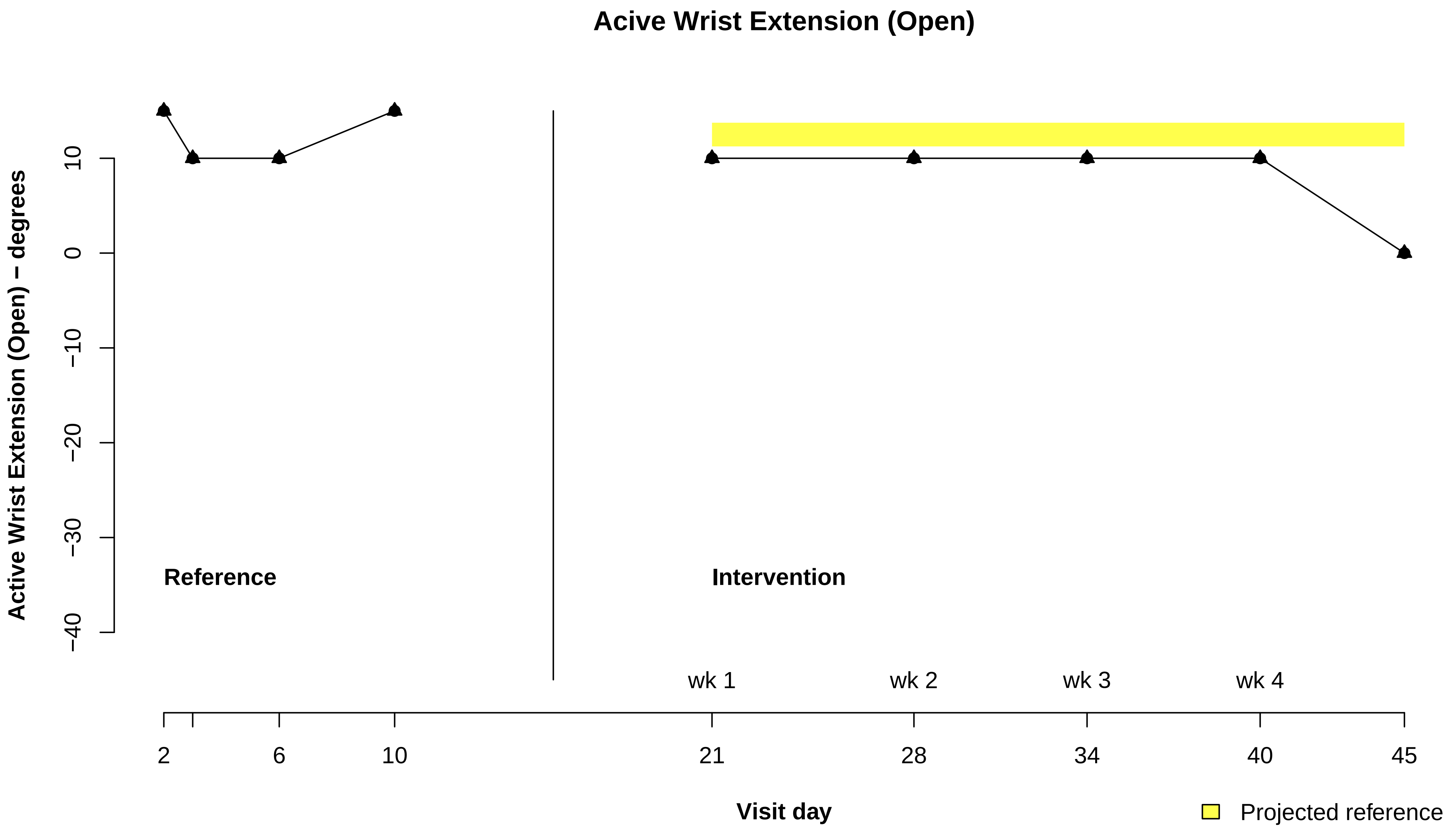

P
Reference phase trend: minimal (0 deg/visit)
Reference phase stability: stable (100% in envelope)
Level change estimate: minimal
Intervention phase 0 deg higher than Reference phase
Average of −2 degree decrease across Intervention visits
44% overlap between phases (PAND)

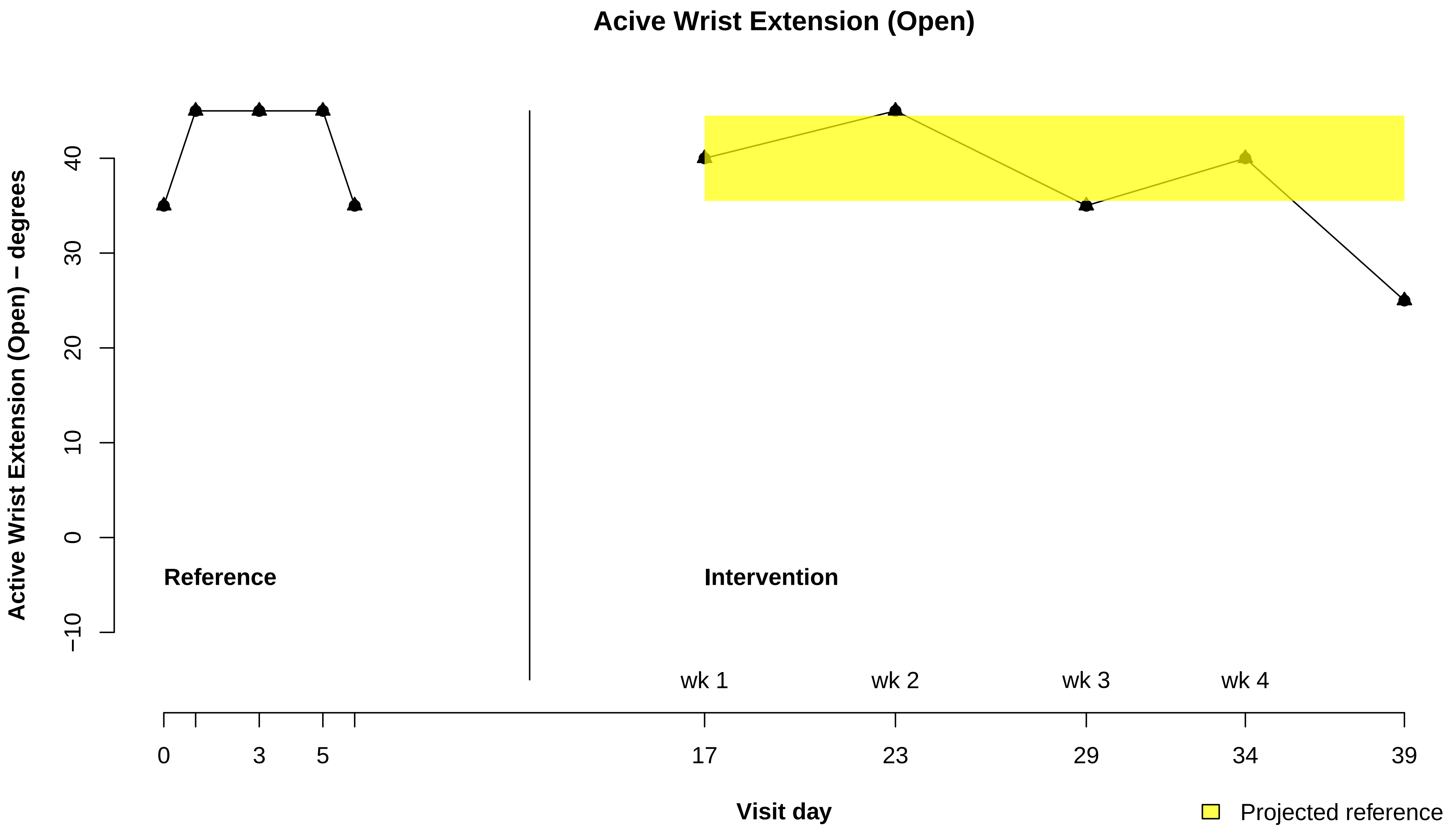

Q
Reference phase trend: minimal (0 deg/visit)
Reference phase stability: stable (100% in envelope)
Level change estimate: minimal
Intervention phase 4 deg higher than Reference phase
Average of −4 degree decrease across Intervention visits
50% overlap between phases (PAND)

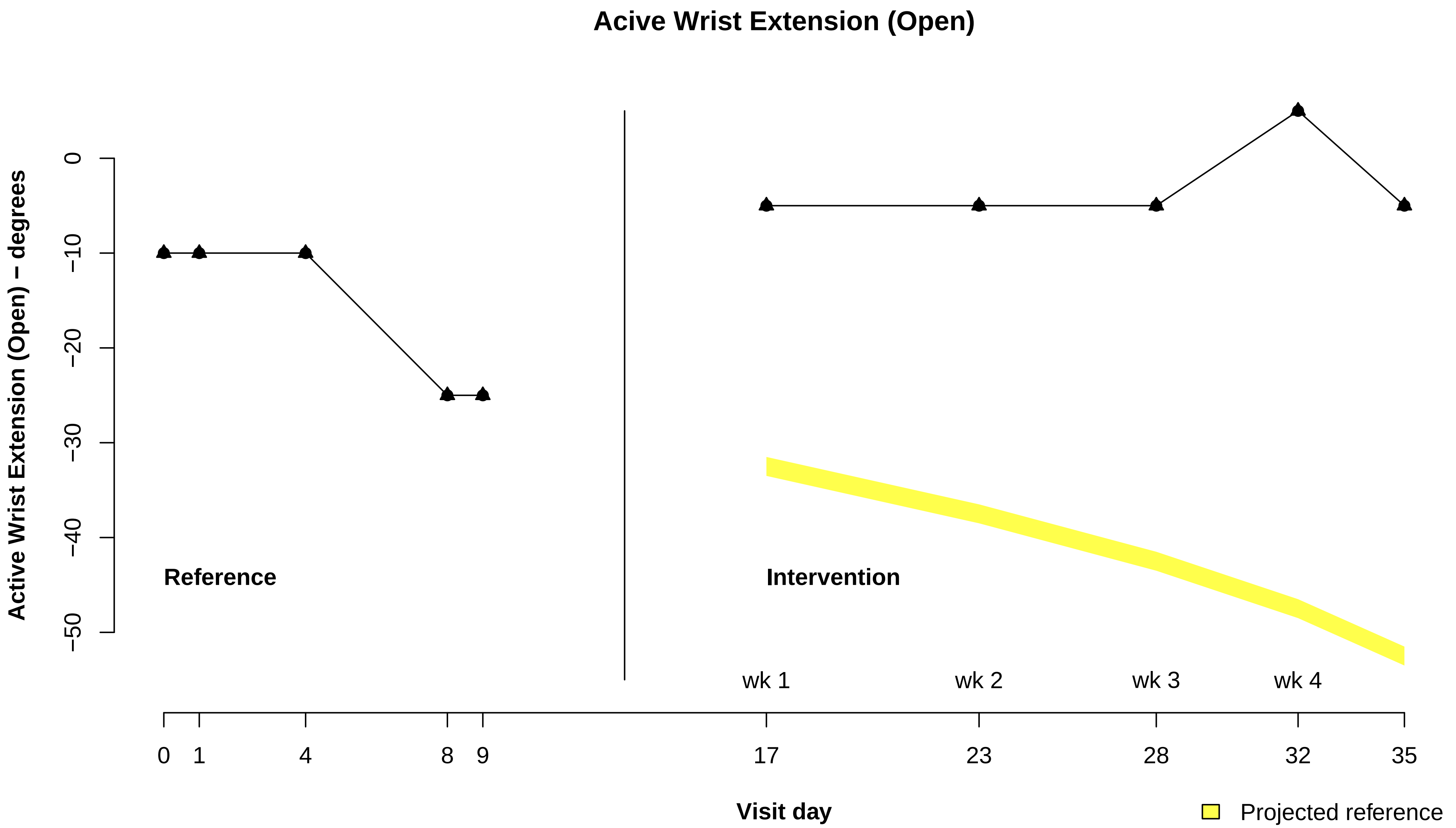

R
Reference phase trend: minimal (−4 deg/visit)
Reference phase stability: stable (100% in envelope)
Level change estimate: large
Intervention phase 24 deg higher than Reference phase
Average of 4 degree increase across Intervention visits
0% overlap between phases (PAND)

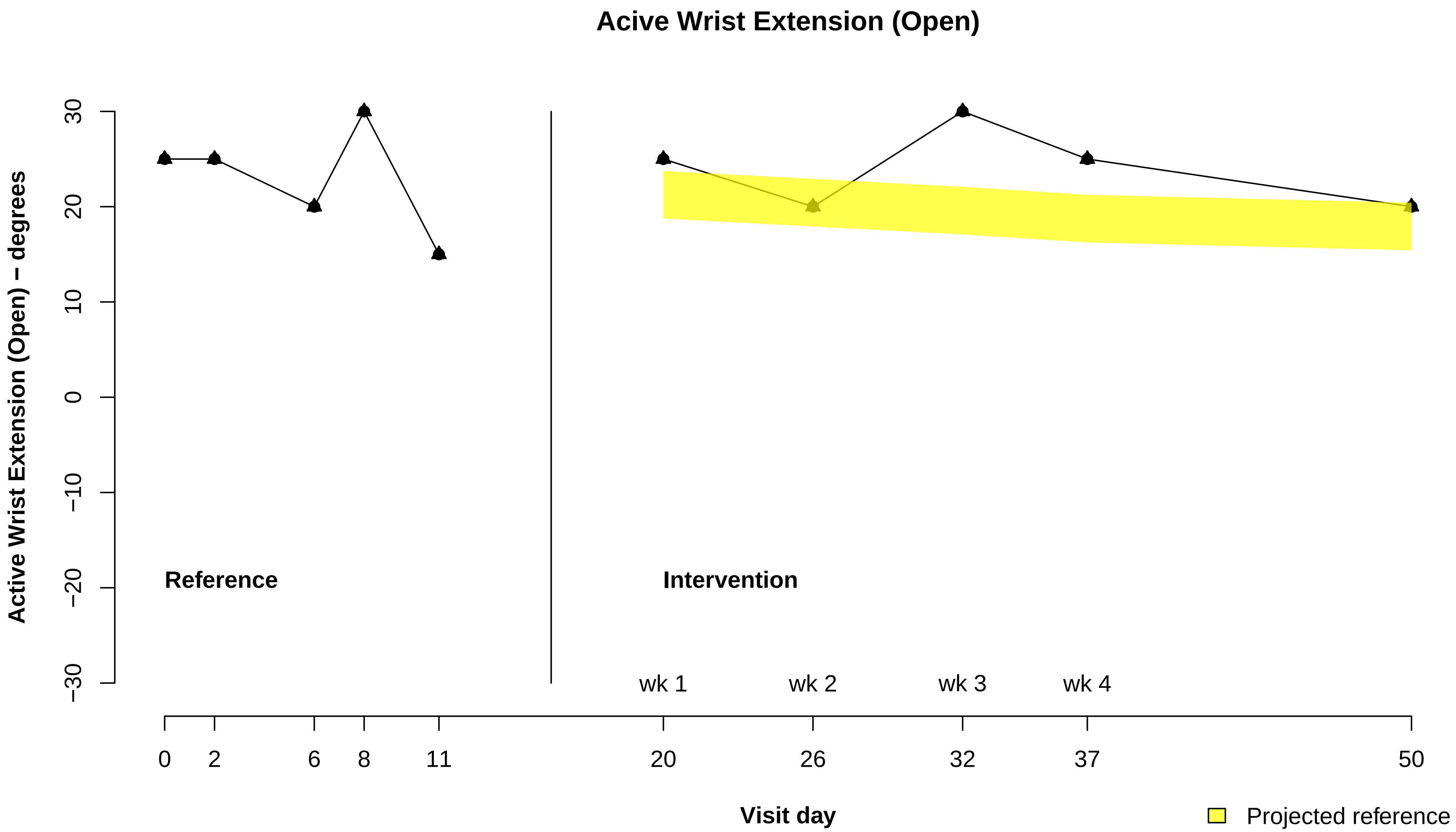

S
Reference phase trend: minimal (−2 deg/visit)
Reference phase stability: stable (80% in envelope)
Level change estimate: moderate
Intervention phase 11 deg higher than Reference phase
Average of 1 degree increase across Intervention visits
10% overlap between phases (PAND)

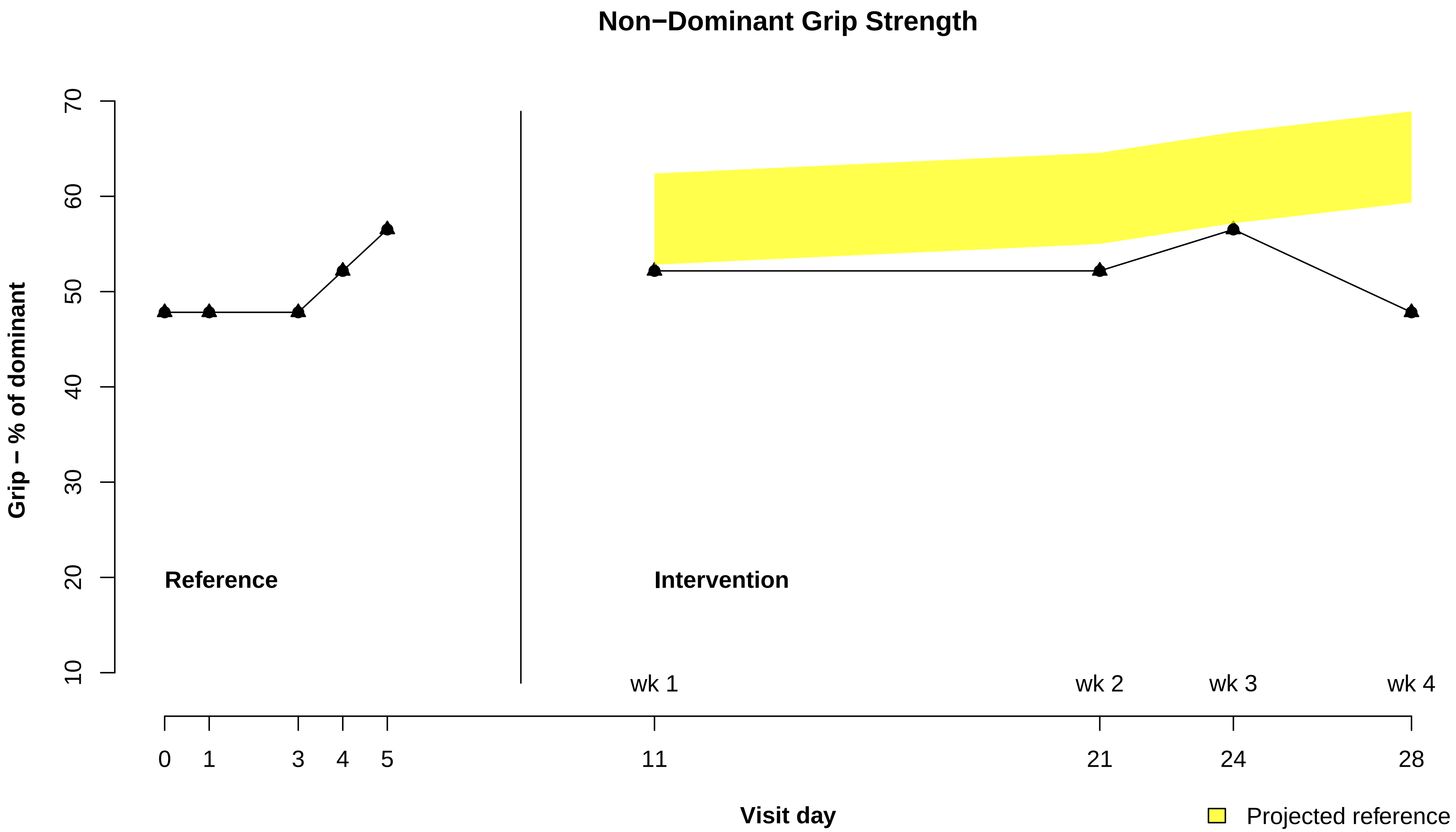

A
Reference phase trend: minimal (2 %/visit)
Reference phase stability: highly variable (40% in envelope)
Level change estimate: minimal
Intervention phase −3 % lower than Reference phase
Average of −4 % decrease across Intervention visits
44% overlap between phases (PAND)

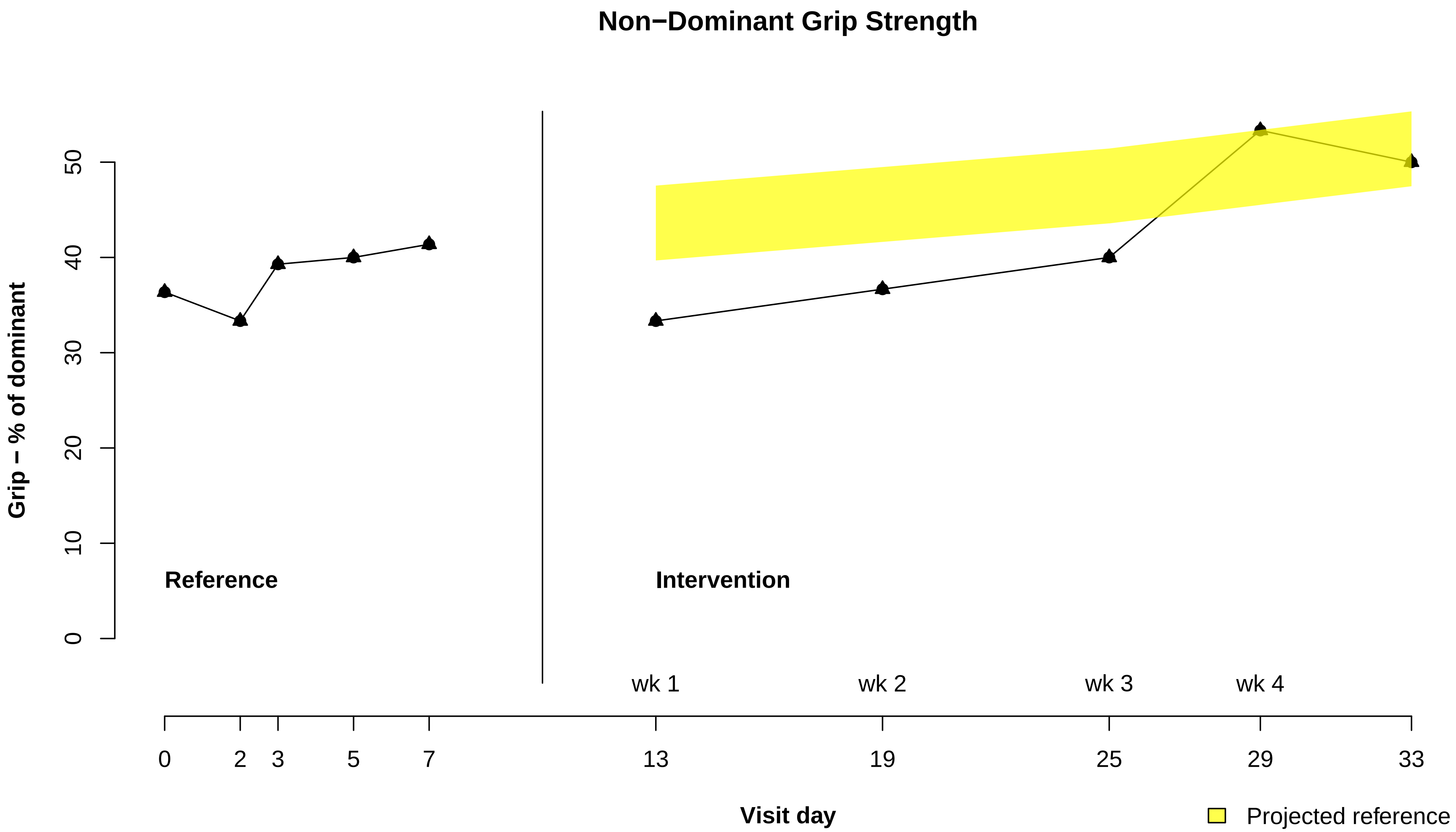

B
Reference phase trend: minimal (1 %/visit)
Reference phase stability: somewhat variable (60% in envelope)
Level change estimate: moderate
Intervention phase −8 % lower than Reference phase
Average of 3 % increase across Intervention visits
30% overlap between phases (PAND)

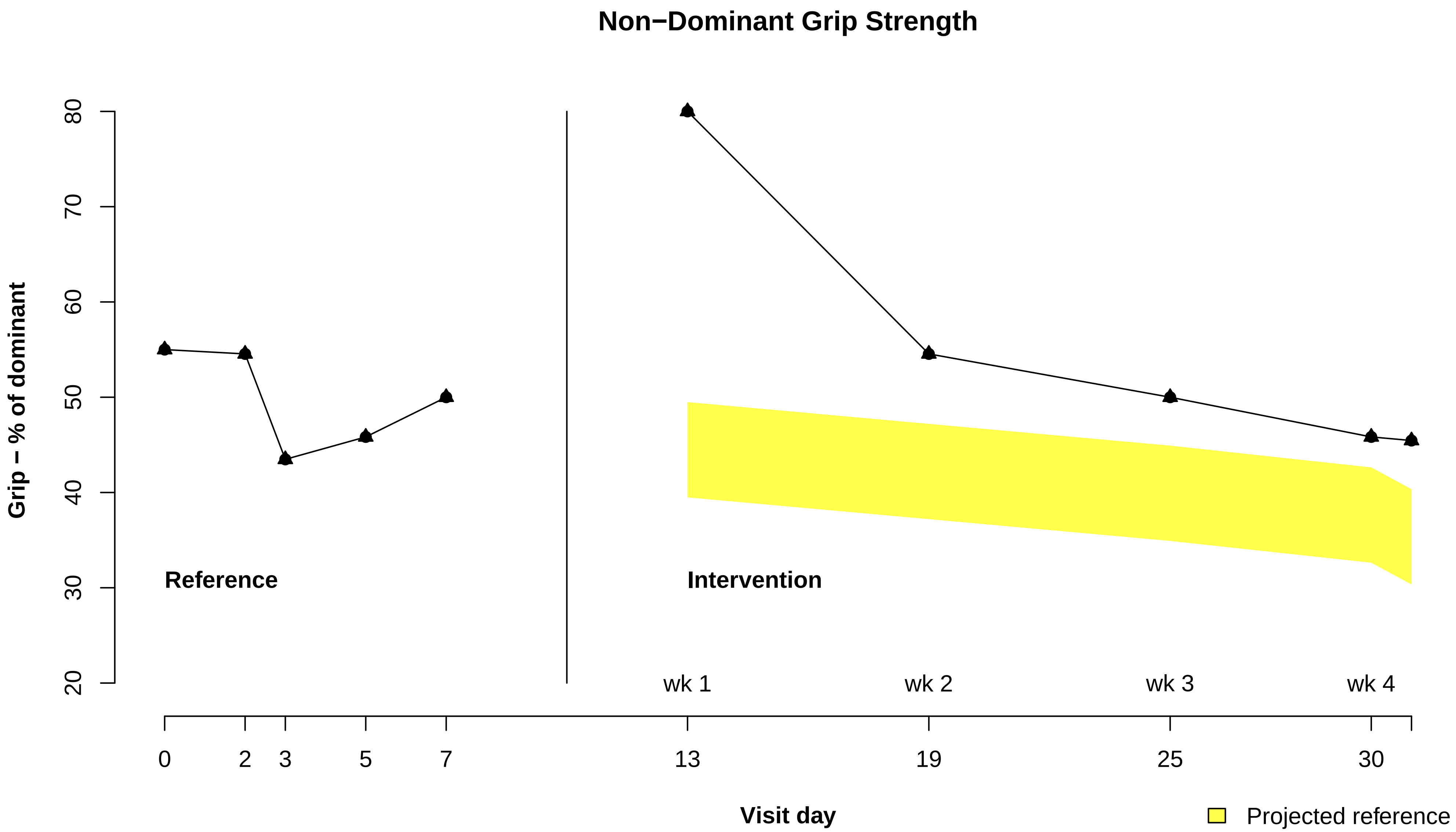

C
Reference phase trend: minimal (−1 %/visit)
Reference phase stability: stable (100% in envelope)
Level change estimate: large
Intervention phase 26 % higher than Reference phase
Average of −7 % decrease across Intervention visits
0% overlap between phases (PAND)

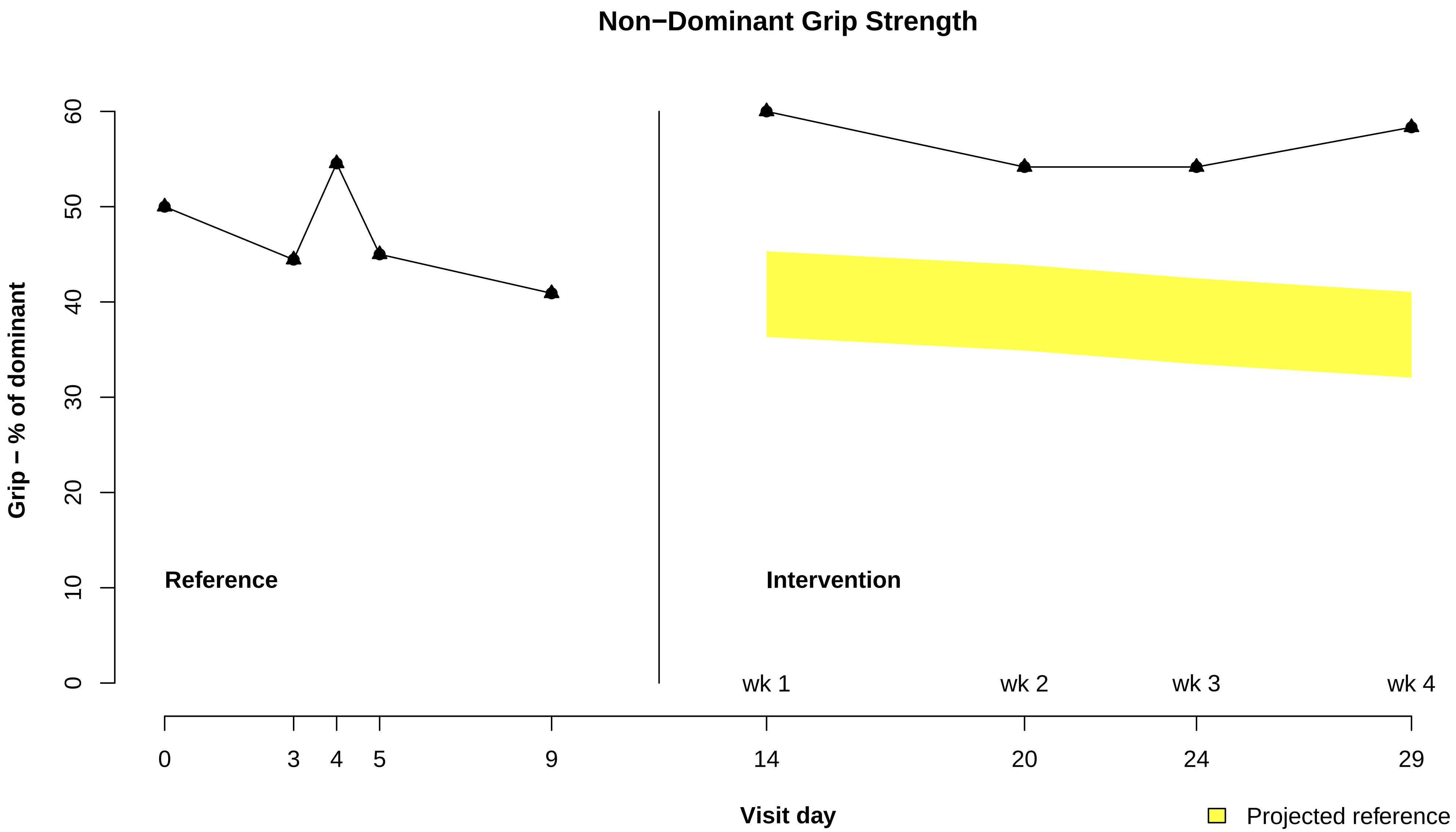

D
Reference phase trend: minimal (−2 %/visit)
Reference phase stability: somewhat variable (60% in envelope)
Level change estimate: large
Intervention phase 17 % higher than Reference phase
Average of 2 % increase across Intervention visits
0% overlap between phases (PAND)

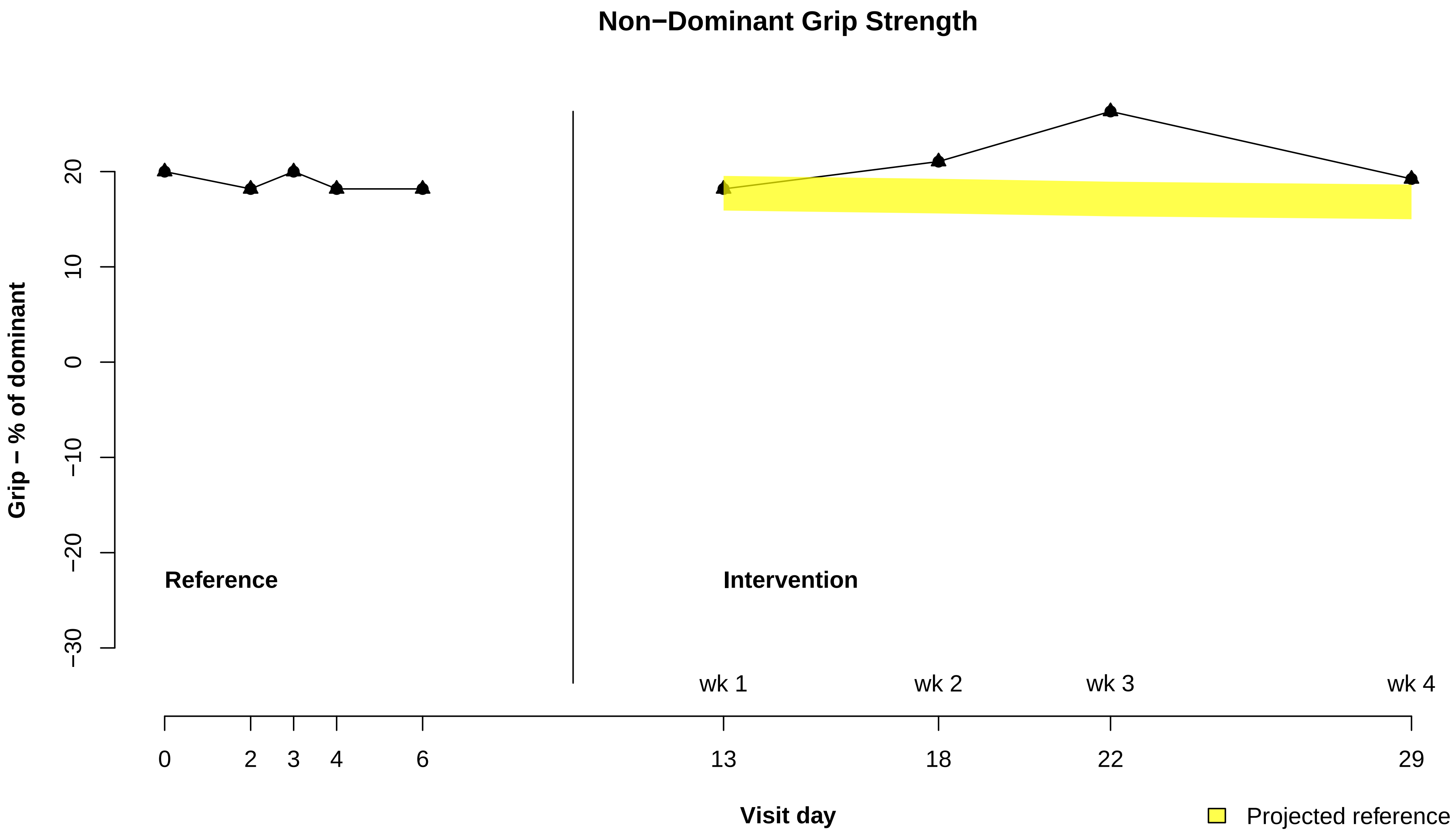

E
Reference phase trend: minimal (0 %/visit)
Reference phase stability: stable (100% in envelope)
Level change estimate: minimal
Intervention phase 3 % higher than Reference phase
Average of 1 % increase across Intervention visits
11% overlap between phases (PAND)

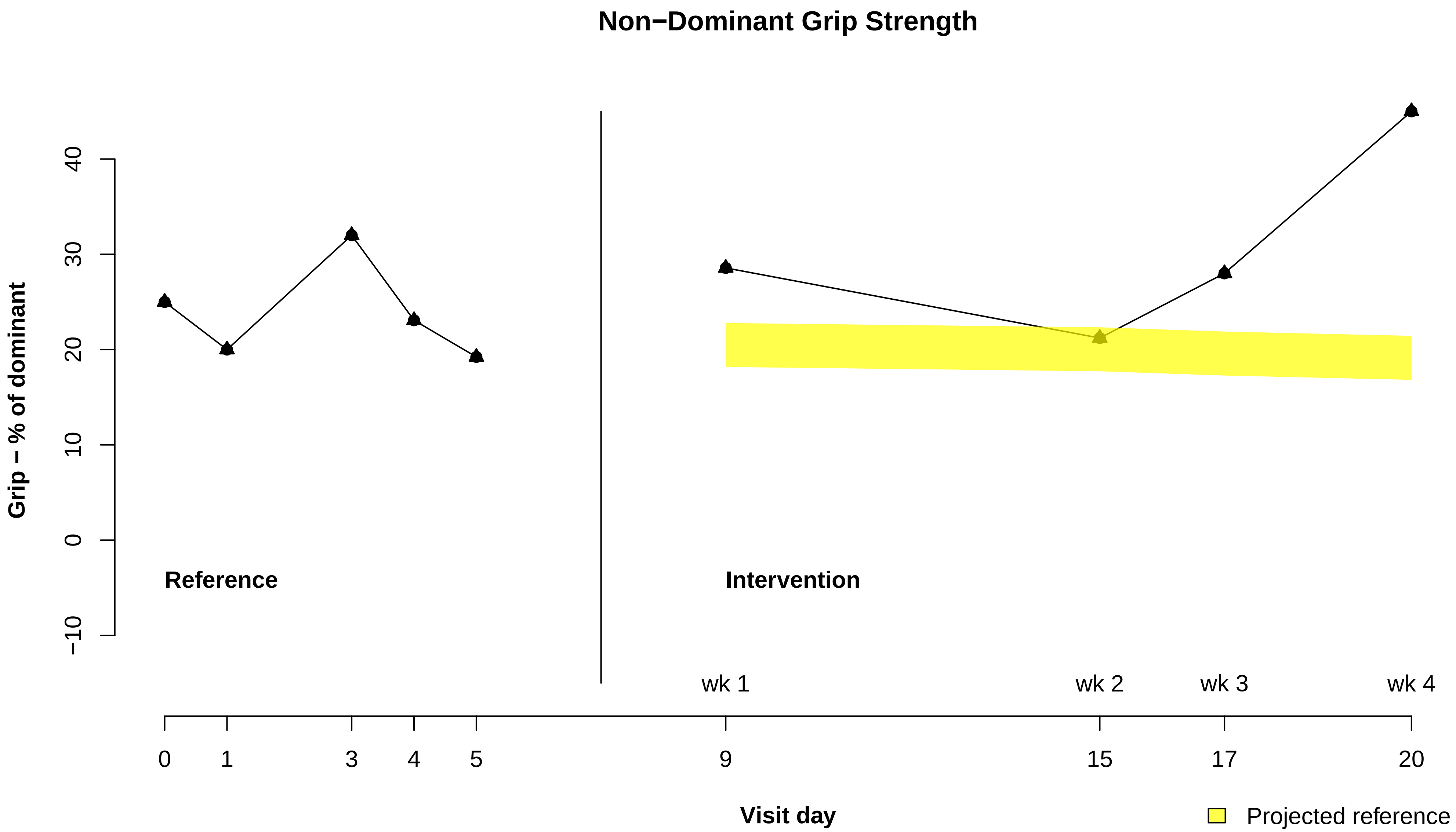

F
Reference phase trend: minimal (−1 %/visit)
Reference phase stability: stable (80% in envelope)
Level change estimate: minimal
Intervention phase 3 % higher than Reference phase
Average of 7 % increase across Intervention visits
11% overlap between phases (PAND)

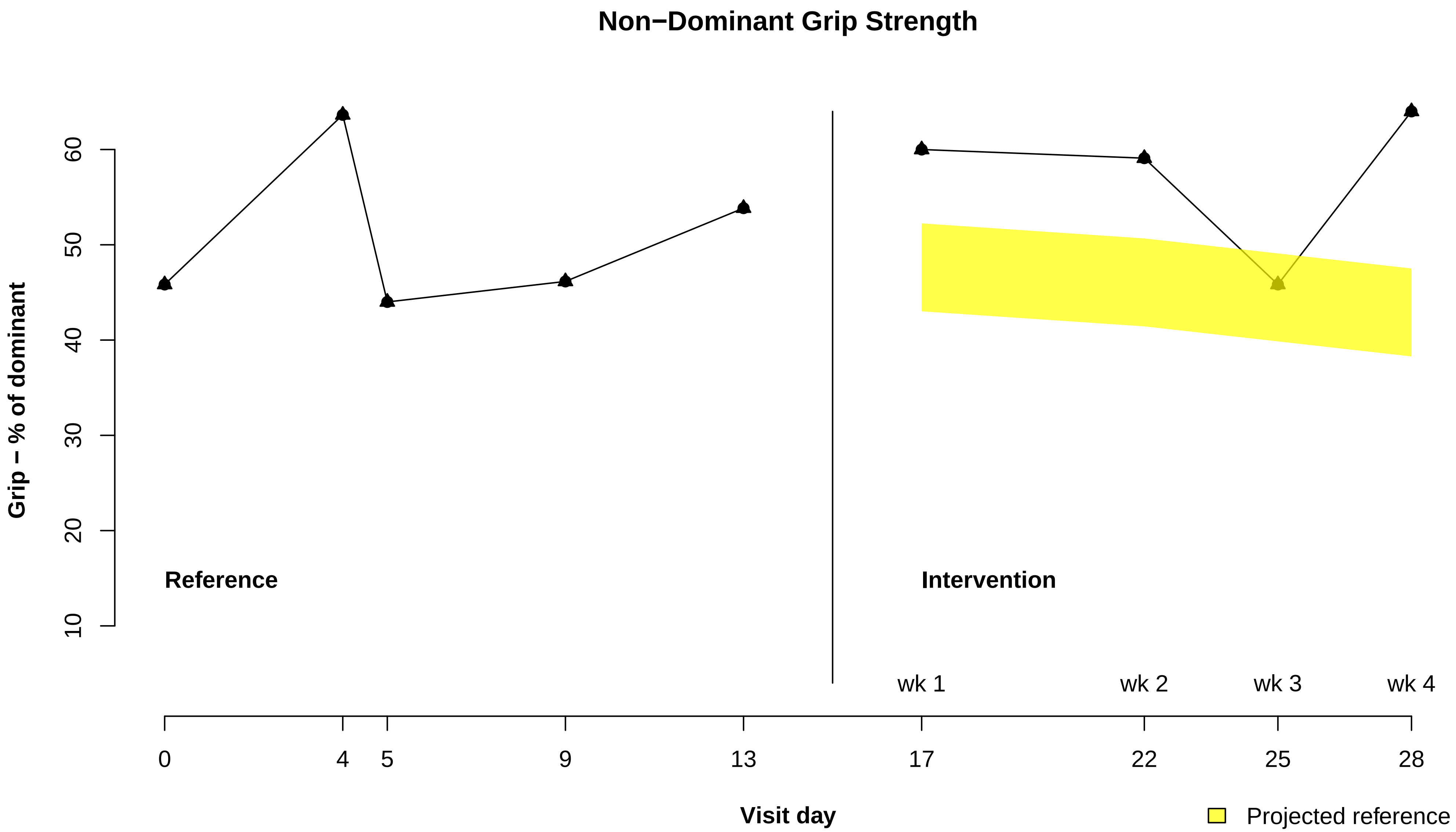

G
Reference phase trend: minimal (2 %/visit)
Reference phase stability: stable (80% in envelope)
Level change estimate: minimal
Intervention phase −1 % lower than Reference phase
Average of −1 % decrease across Intervention visits
22% overlap between phases (PAND)

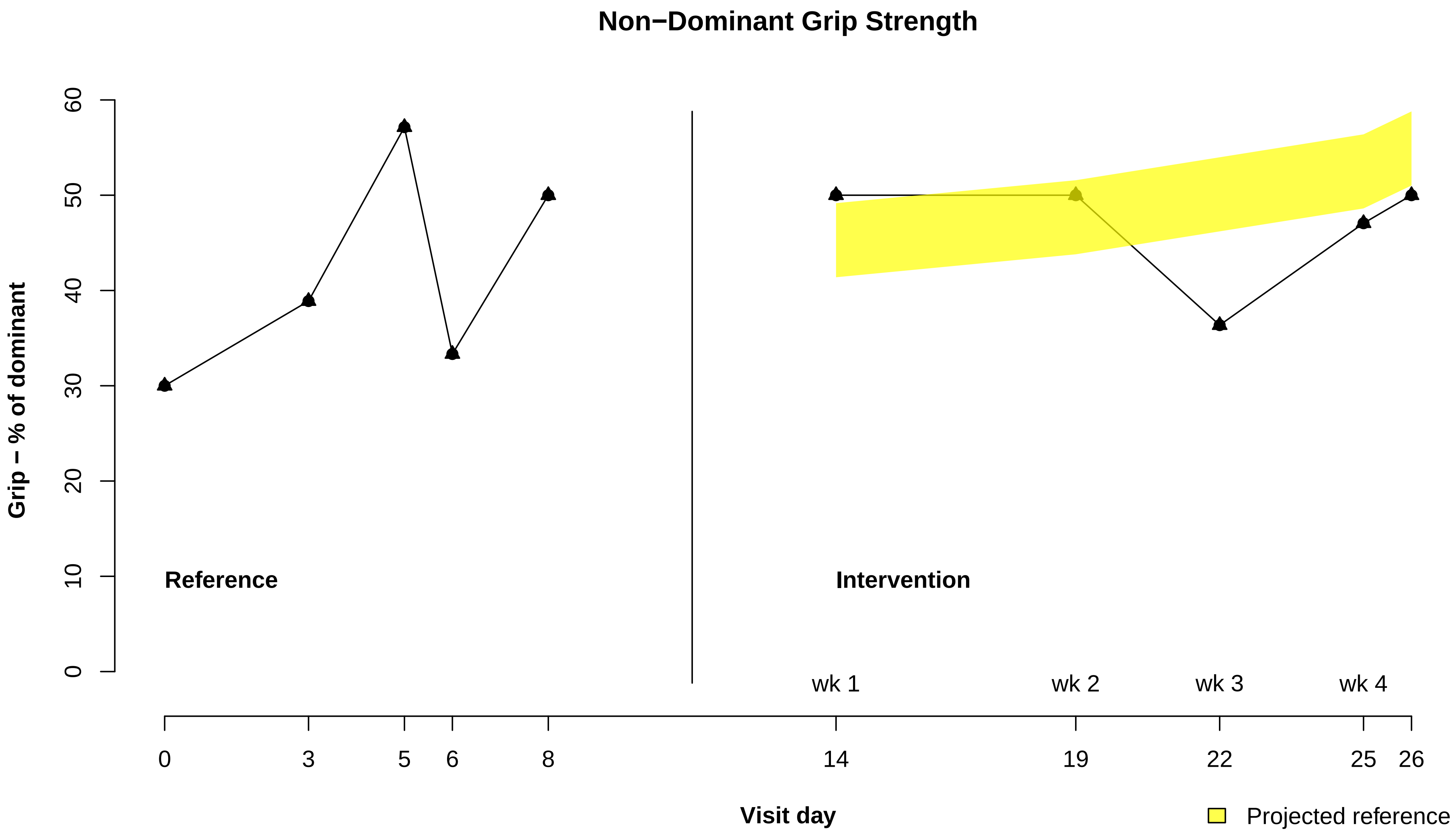

H
Reference phase trend: minimal (5 %/visit)
Reference phase stability: stable (100% in envelope)
Level change estimate: moderate
Intervention phase −10 % lower than Reference phase
Average of −5 % decrease across Intervention visits
50% overlap between phases (PAND)

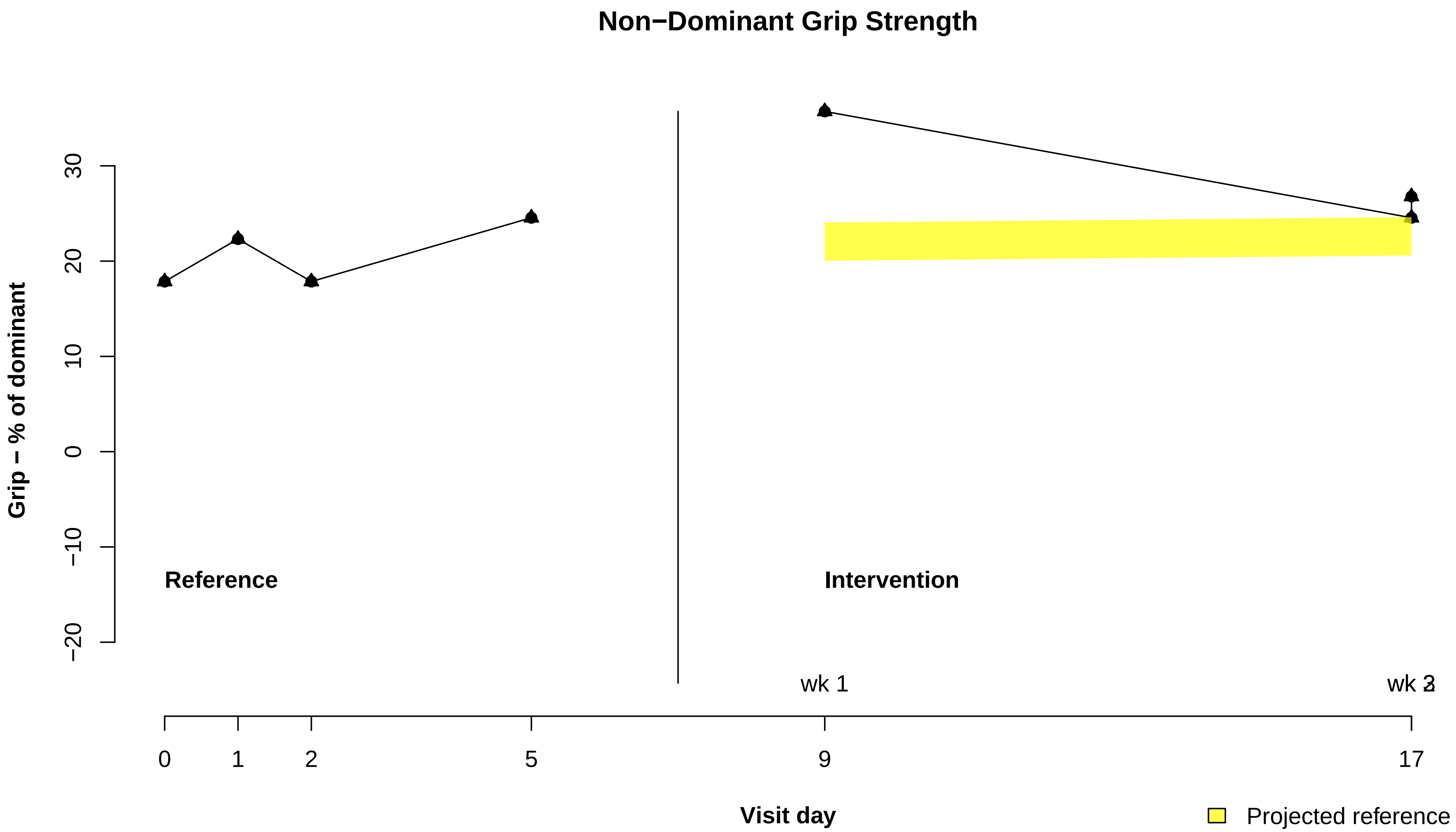

I

Reference phase trend: minimal (2 %/visit)

Reference phase stability: stable (100% in envelope)

Level change estimate: moderate

Intervention phase 7 % higher than Reference phase

Average of −7 % decrease across Intervention visits

29% overlap between phases (PAND)

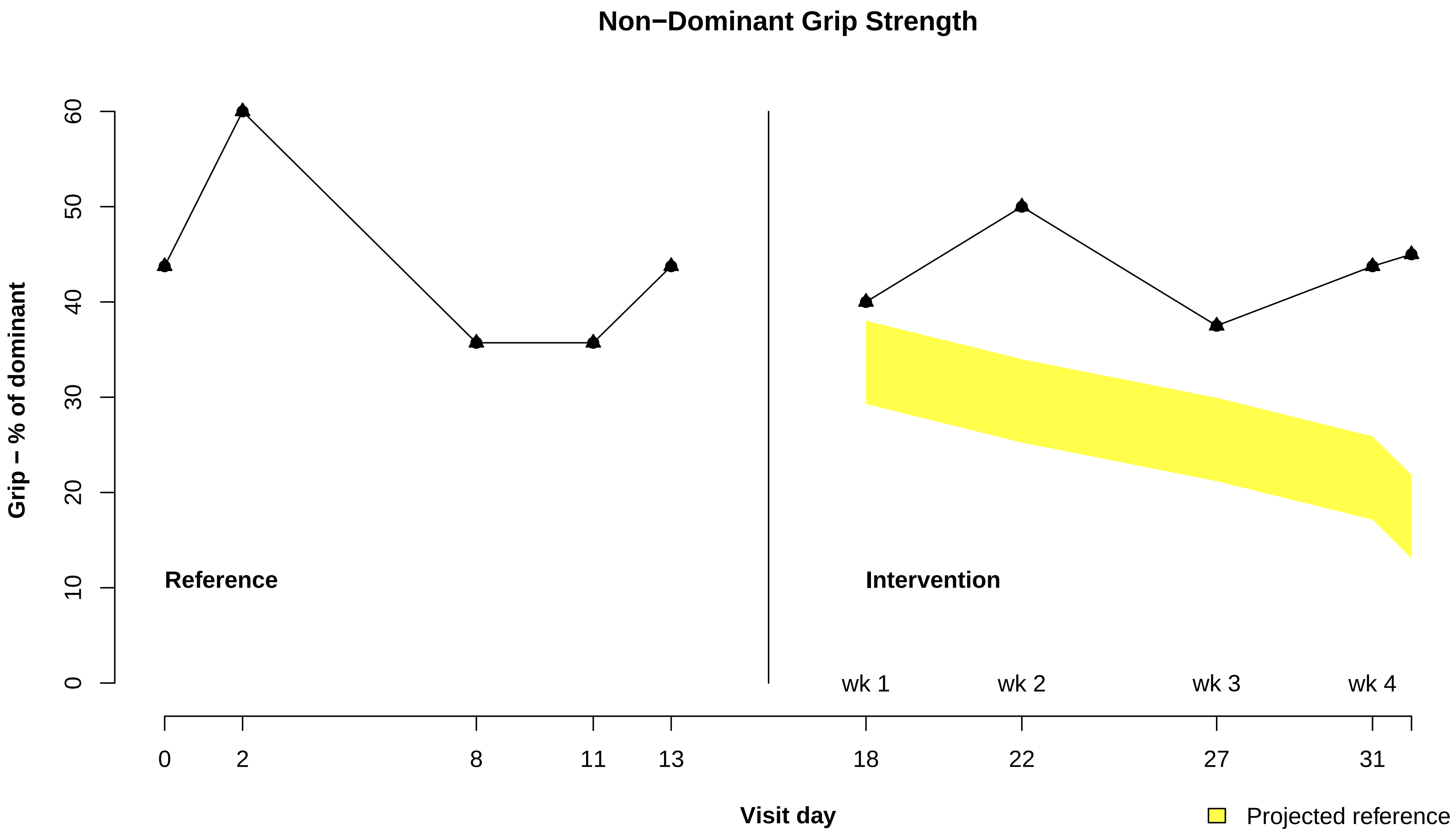

J
Reference phase trend: minimal (0 %/visit)
Reference phase stability: stable (80% in envelope)
Level change estimate: minimal
Intervention phase −3 % lower than Reference phase
Average of 1 % increase across Intervention visits
30% overlap between phases (PAND)

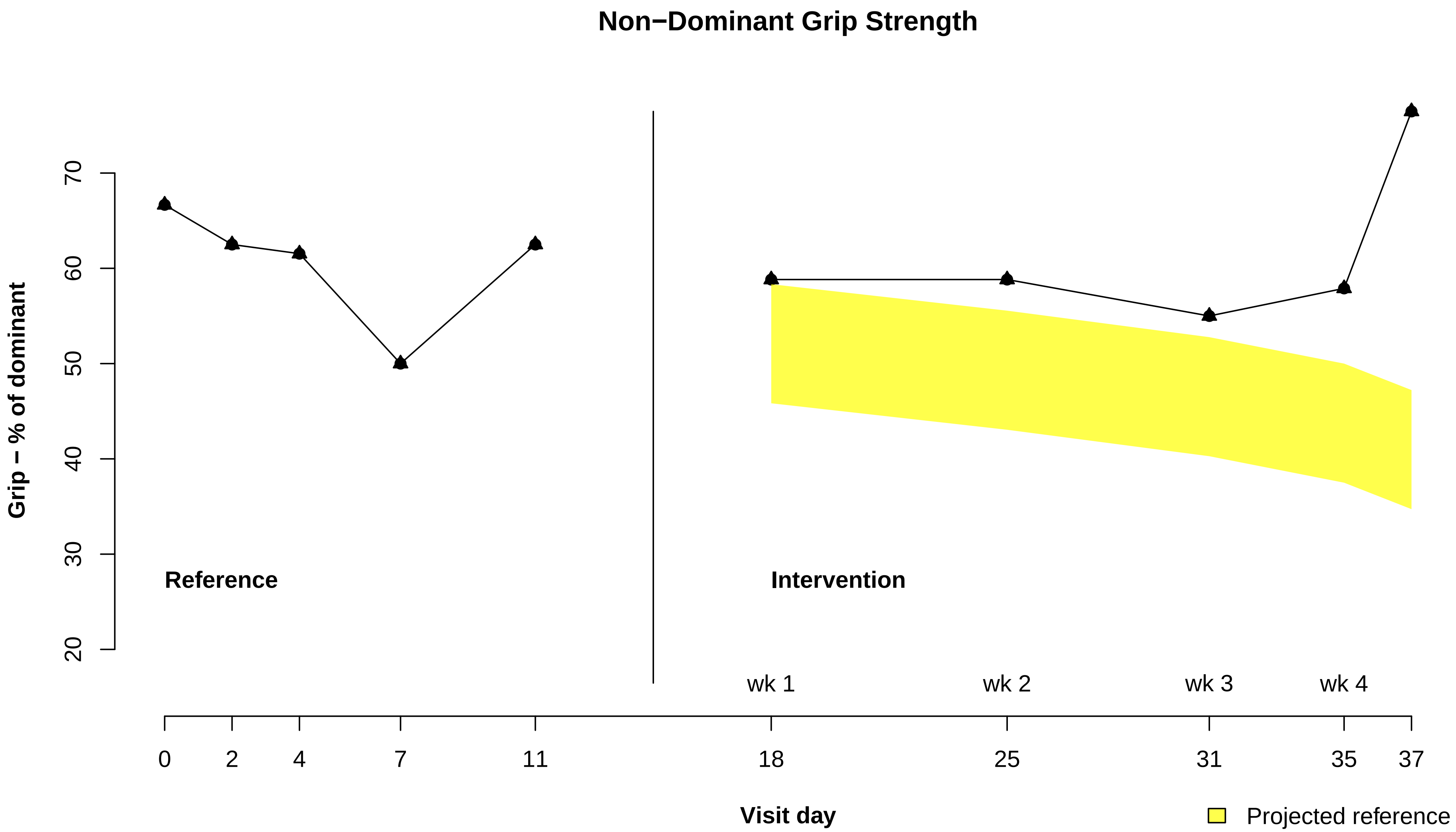

K
Reference phase trend: minimal (−1 %/visit)
Reference phase stability: highly variable (0% in envelope)
Level change estimate: minimal
Intervention phase −5 % lower than Reference phase
Average of 5 % increase across Intervention visits
30% overlap between phases (PAND)

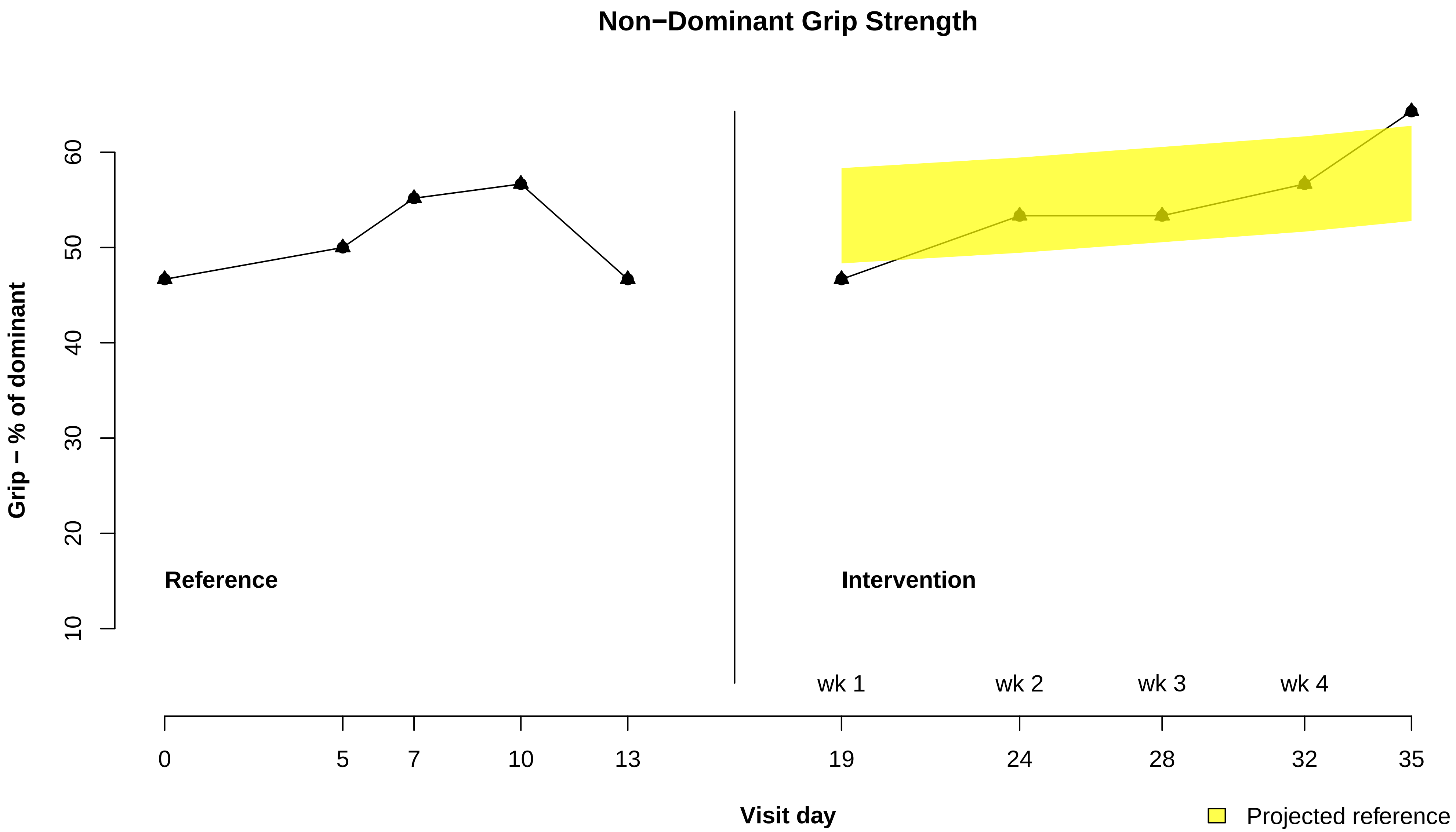

L
Reference phase trend: minimal (0 %/visit)
Reference phase stability: stable (100% in envelope)
Level change estimate: minimal
Intervention phase −5 % lower than Reference phase
Average of 4 % increase across Intervention visits
30% overlap between phases (PAND)

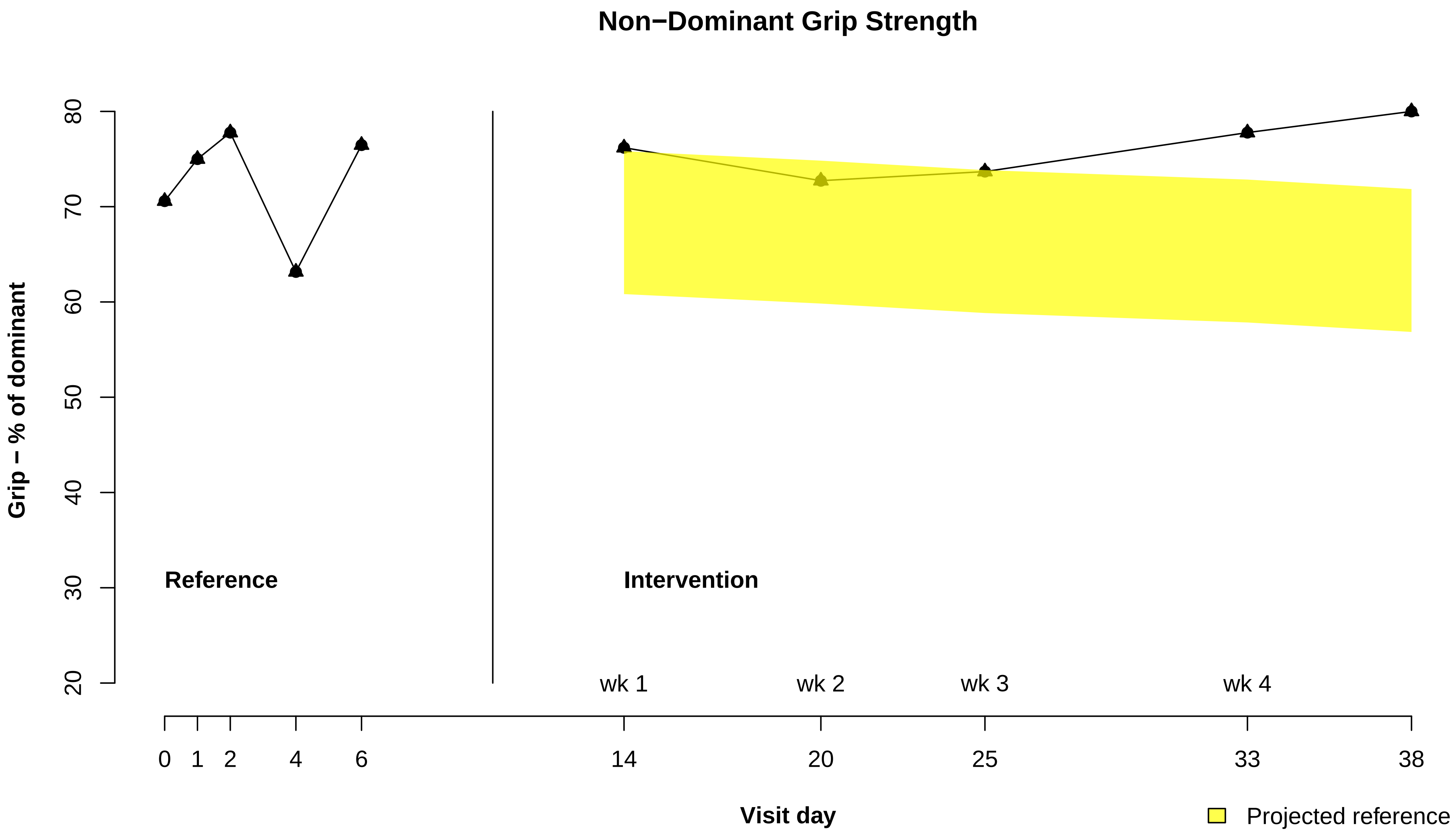

M
Reference phase trend: minimal (1 %/visit)
Reference phase stability: stable (80% in envelope)
Level change estimate: minimal
Intervention phase −3 % lower than Reference phase
Average of −1 % decrease across Intervention visits
40% overlap between phases (PAND)

Non−Dominant Grip Strength

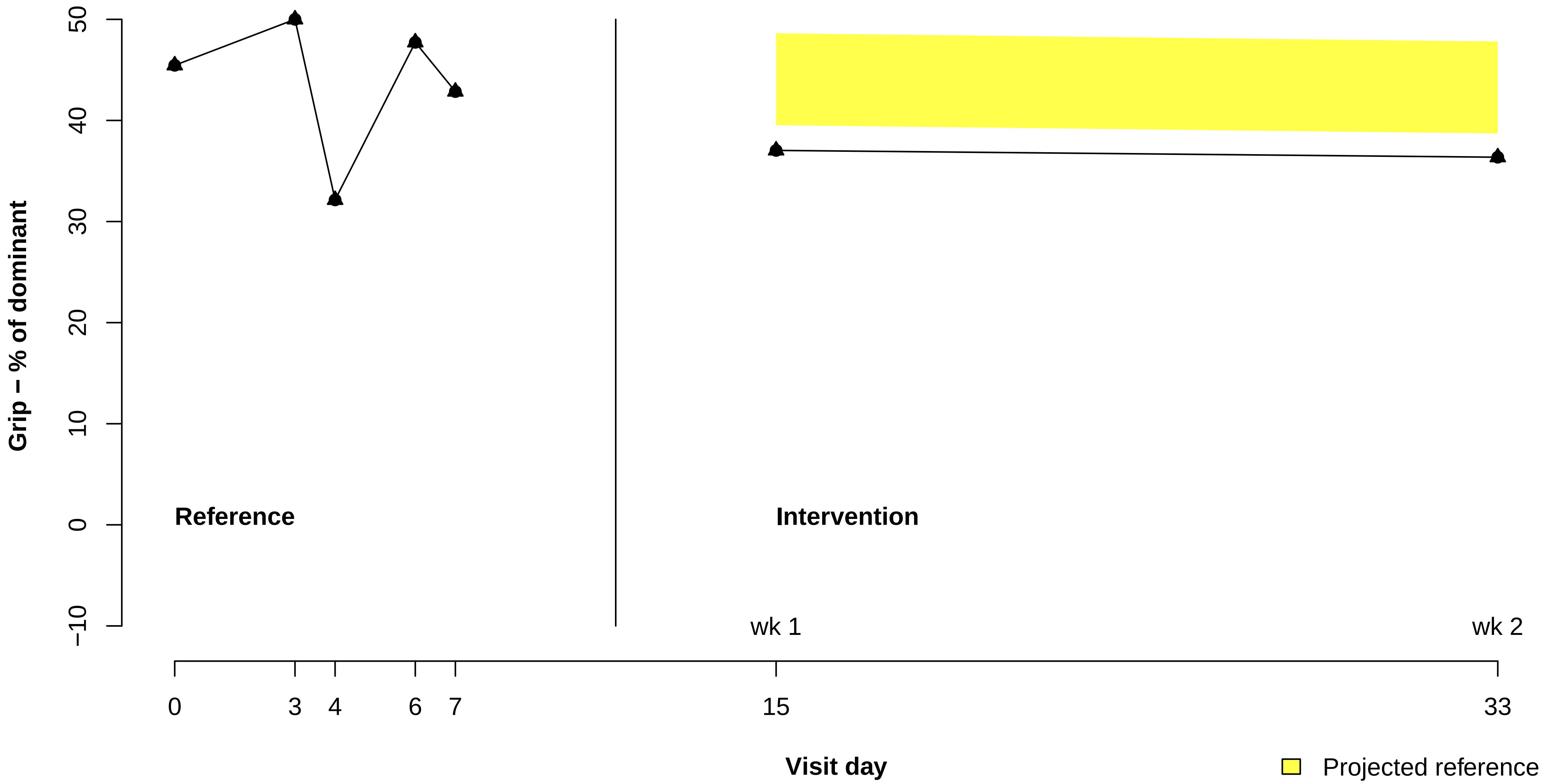

N
Reference phase trend: minimal (−1 %/visit)
Reference phase stability: stable (80% in envelope)
Level change estimate: minimal
Intervention phase −5 % lower than Reference phase
Average of 0 % decrease across Intervention visits
29% overlap between phases (PAND)

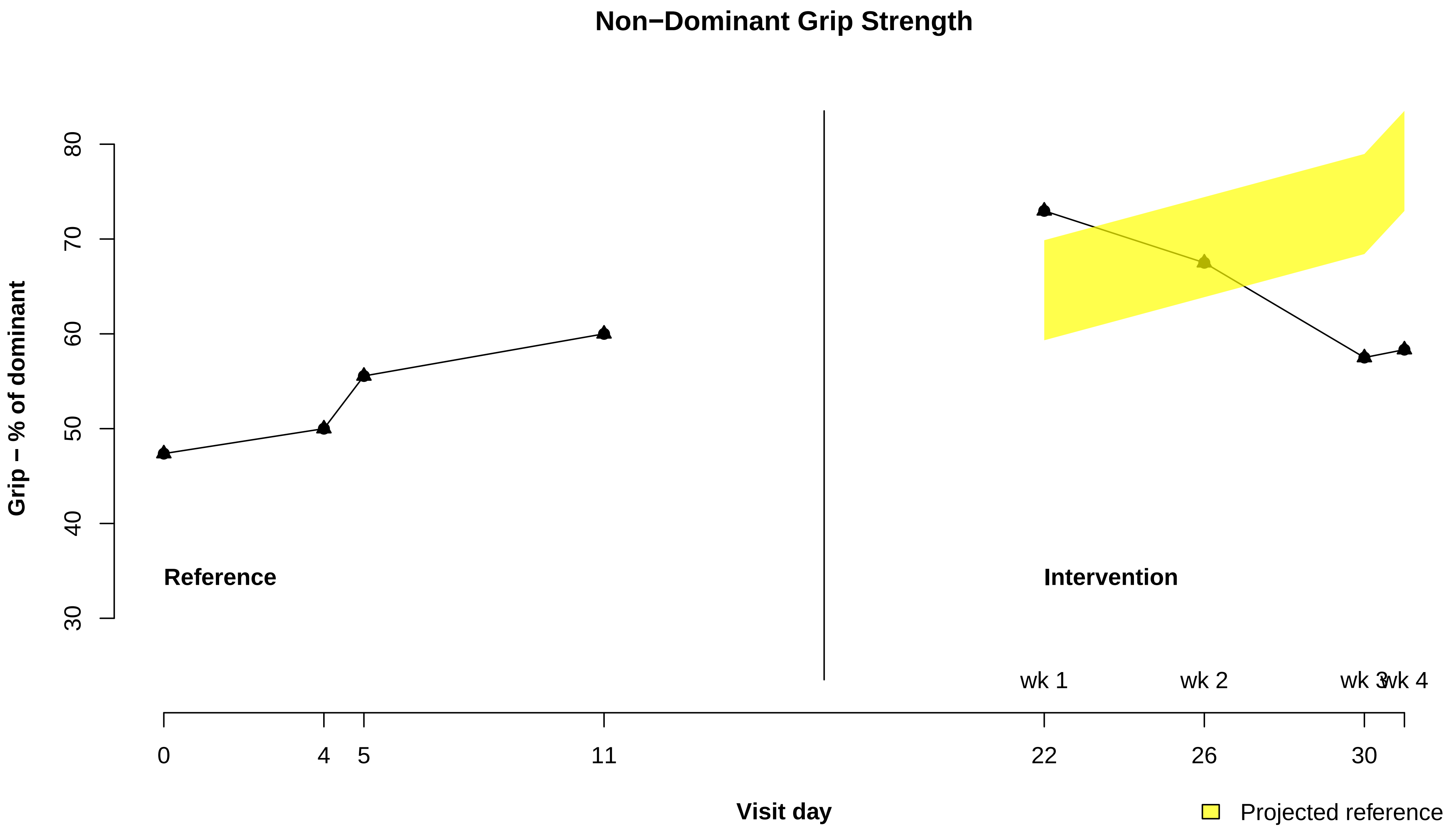

O
Reference phase trend: minimal (4 %/visit)
Reference phase stability: highly variable (50% in envelope)
Level change estimate: moderate
Intervention phase 8 % higher than Reference phase
Average of −9 % decrease across Intervention visits
38% overlap between phases (PAND)

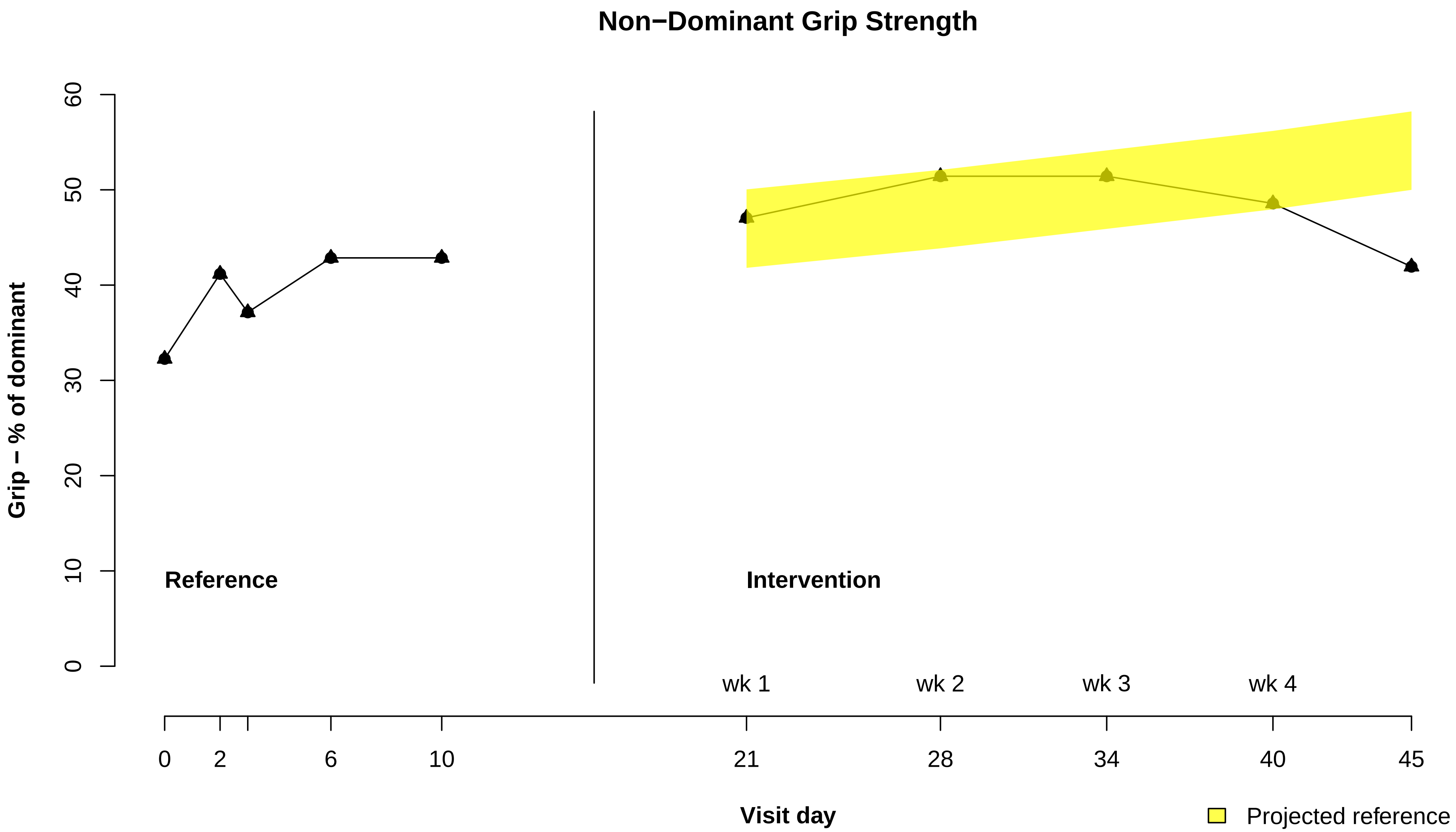

P
Reference phase trend: minimal (3 %/visit)
Reference phase stability: somewhat variable (60% in envelope)
Level change estimate: minimal
Intervention phase 3 % higher than Reference phase
Average of −4 % decrease across Intervention visits
40% overlap between phases (PAND)

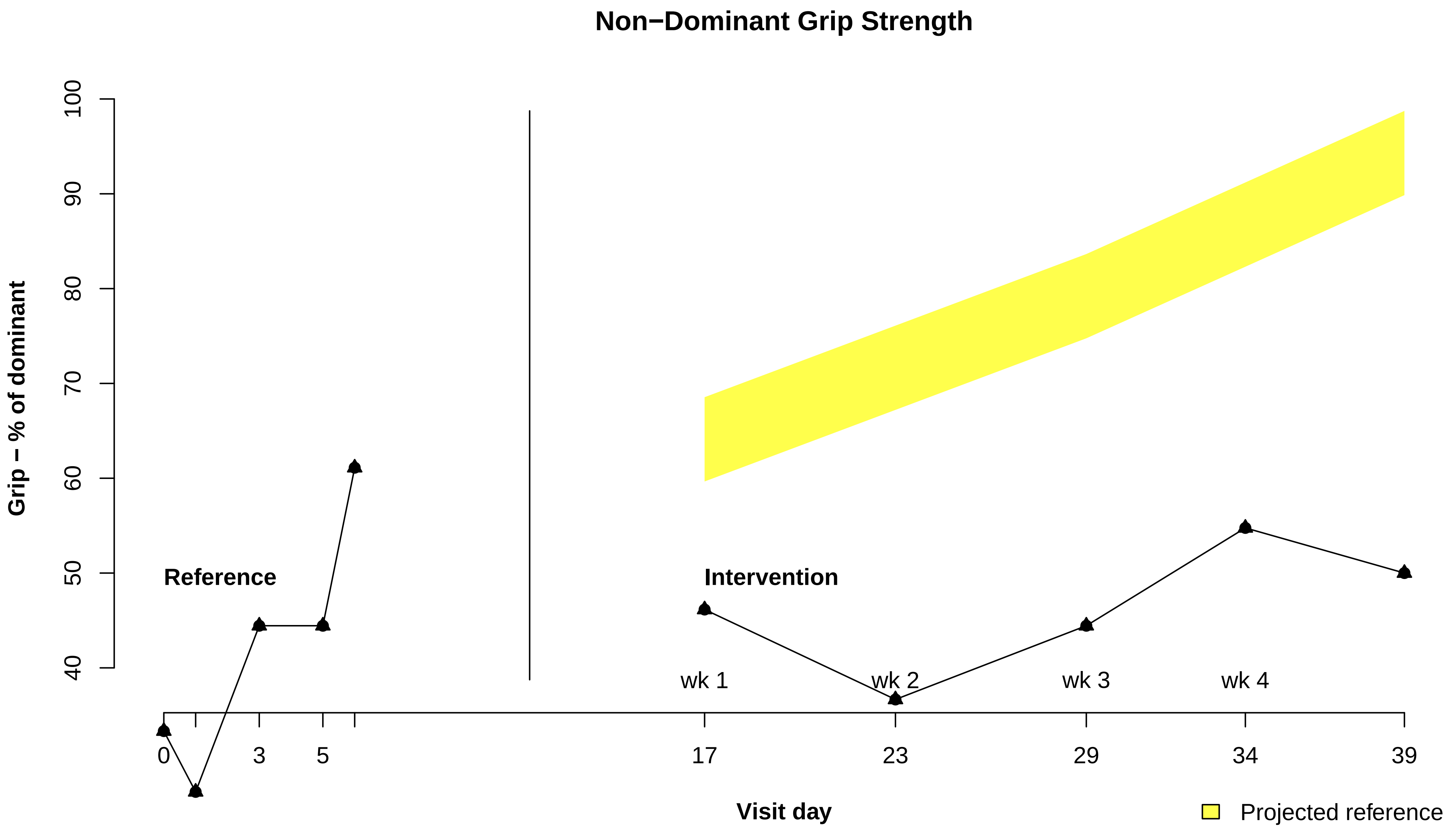

Q
Reference phase trend: moderate (7 %/visit)
Reference phase stability: highly variable (20% in envelope)
Level change estimate: large
Intervention phase −18 % lower than Reference phase
Average of −6 % decrease across Intervention visits
50% overlap between phases (PAND)

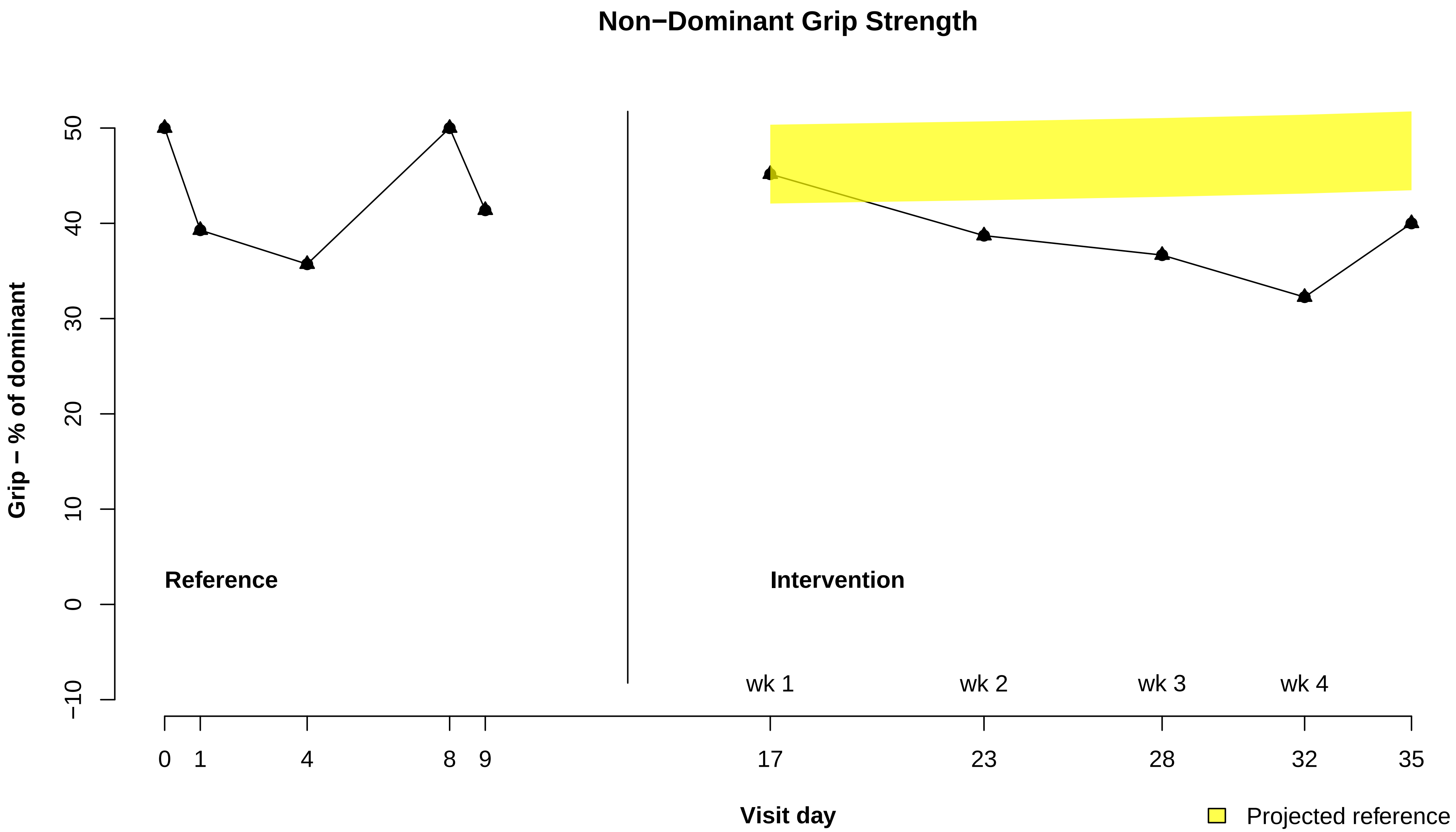

R
Reference phase trend: minimal (−2 %/visit)
Reference phase stability: stable (100% in envelope)
Level change estimate: minimal
Intervention phase 4 % higher than Reference phase
Average of 1 % increase across Intervention visits
20% overlap between phases (PAND)

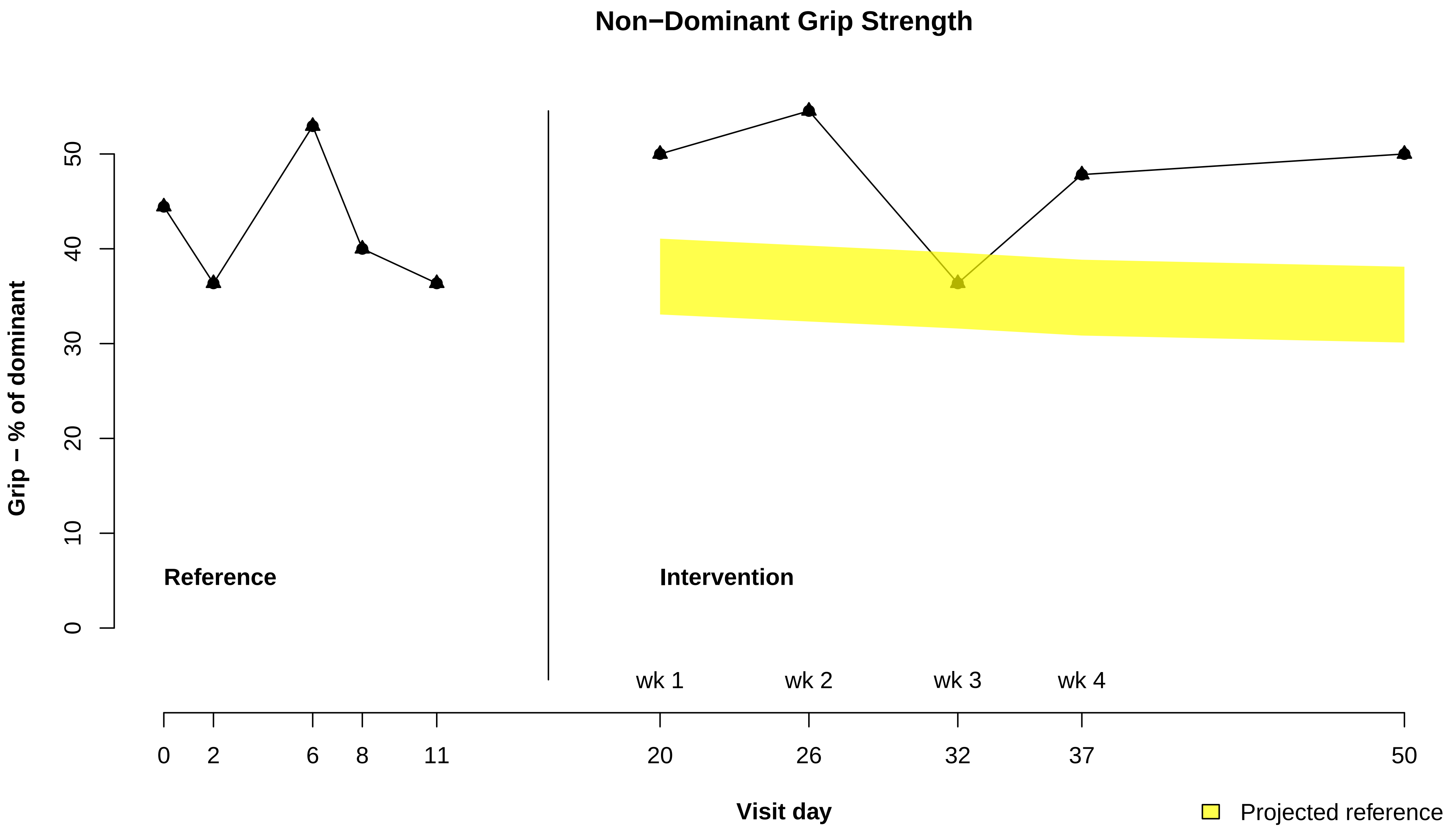

S
Reference phase trend: minimal (−2 %/visit)
Reference phase stability: stable (80% in envelope)
Level change estimate: moderate
Intervention phase 12 % higher than Reference phase
Average of 2 % increase across Intervention visits
10% overlap between phases (PAND)
