# Supplementary material for: A biofeedback-enhanced therapeutic exercise video game intervention for young people with cerebral palsy: A randomized single-case experimental design feasibility study
Source: PLoS One. 2020 Jun 22;15(6):e0234767. doi: 10.1371/journal.pone.0234767 (PMC7307764; doi:10.1371/journal.pone.0234767)
Supplement: S1 Table — (DOCX) [file pone.0234767.s008.docx]

S9 Table 1 COPM Goals

| **ID** | **Primary COPM goal** | **Goal setter** |
| --- | --- | --- |
| A | Spread thumb easier to use joystick when playing video games | P |
| B | Get hand in good position to clasp earring | P |
| C | Tie the first knot of his sweat pant waist laces | C |
| D | Use helping hand to tie hair back and twist elastic | P |
| E | Open hand to hold yogurt cup while opening | P |
| F | Pull shoelaces tighter | P |
| G | Hold hand straight position to text using both thumbs | P |
| H | Stabilize fork while cutting thick meat | P |
| I | Hold toothbrush while putting on toothpaste | P |
| J | Hold toothbrush while putting on toothpaste | C |
| K | Pull over shirt faster using both hands | C |
| L | Sweep hard with tight grip in good position for curling | P |
| M | Hold monkey-bars longer, let go with my hand and grab the next rung | P |
| N | Stabilize fork while cutting soft food | P |
| O | Hold mug with liquid while pouring with the dominant | C |
| P | Hold game controller with better alignment for longer while playing games | P |
| Q | Use helping hand to tie hair back and twist elastic | P |
| R | Hold full water bottle while uncapping lid | P |
| S | Carry shopping bag for longer before getting tired | C |

Participant-P, Caregiver-C
