# Supplementary material for: A biofeedback-enhanced therapeutic exercise video game intervention for young people with cerebral palsy: A randomized single-case experimental design feasibility study
Source: PLoS One. 2020 Jun 22;15(6):e0234767. doi: 10.1371/journal.pone.0234767 (PMC7307764; doi:10.1371/journal.pone.0234767)
Supplement: S2 Table — (DOCX) [file pone.0234767.s009.docx]

S10 Table 2 SEAS subscale scores

|  | **Week 1** | **Week 4** |
| --- | --- | --- |
| **Overall** | 2.00 (1.25) | 2.00 (1.25) |
| **Personal growth** | 2.25 (0.75) | 2.00 (0.75) |
| **Psychological Engagement** | 3.00 (0.50) | 2.50 (0.50) |
| **Meaningful Interactions** | 2.00 (0.00) | 2.00 (0.00) |
| **Choice and Control** | 1.25 (1.25) | 2.00 (0.75) |
|  |  |  |

Median (IQR) for SEAS overall and subfields scores across participants (N=19). 7-point Likert scale scored from +3 (strongly agree) to -3 (strongly disagree). SEAS assessed at the end of week 1 and 4, Intervention phase.
