# Supplementary material for: A biofeedback-enhanced therapeutic exercise video game intervention for young people with cerebral palsy: A randomized single-case experimental design feasibility study
Source: PLoS One. 2020 Jun 22;15(6):e0234767. doi: 10.1371/journal.pone.0234767 (PMC7307764; doi:10.1371/journal.pone.0234767)
Supplement: Attachment — Submitted filename: Response to Reviewers.docx [file pone.0234767.s010.docx]

**Response to Reviewers - PONE-D-19-22575**

| **#** | **Review** | **Response** |
| --- | --- | --- |
|  | **Editor’s comments** |  |
| 1 | The coherence between the study declared goal and the actual presentation of results has been questioned, as well as the way in which the results are presented, and the significance evaluated- Please address these concerns properly. | Thank you for your comments. In our responses to reviewer 2 you will see that their concerns regarding the presentation of the results and the evaluation of significance have been addressed.  In summary, regarding the main aim of this study, we assess feasibility according to recommendations from Thabane et al 2010, and the CONSORT extension for reporting feasibility studies (Eldridge et al 2016). This framework highlights that the aim of feasibility testing can be towards assessing any of four classifications: process, resources, management and scientific. “Scientific” here refers to treatment safety and estimation of treatment effect and its variance. Motor function assessments were completed for this reason.  It is also worth noting that statistical analyses were intentionally not conducted. In fact, the feasibility reporting guide we follow specifically discourages emphasizing statistical significance in studies of this type mainly due to low powered samples. We therefore follow the Single-Case Reporting Guideline In BEhavioural Interventions ([SCRIBE](https://psycnet.apa.org/fulltext/2016-17384-001.html#appendix_arc_4_1_10)), which recommends the presentation of a mixed ‘visual and statistical’ approach (Manolov et al 2014). The methods are considered complementary rather than mutually exclusive. We acknowledge that there is no universal gold-standard for single-case design studies, and it is for this reason we provide a mixed analysis approach.  Separating primary and secondary aims seems to have caused this confusion. To clarify, we have explicitly stated which aspects of feasibility criteria we address and have:   - Added context around feasibility to the introduction - Re-organized the methods to show that the current investigation focuses on process and scientific feasibility. - Added a summary table of results as Reviewer 2 requested   You can find the specific changes made to the manuscript in the response to Reviewer 2, general comments # 1 and 3. |
| 2 | Please ensure that your manuscript meets PLOS ONE's style requirements, including those for file naming. The PLOS ONE style templates can be found at <http://www.journals.plos.org/plosone/s/file?id=wjVg/PLOSOne_formatting_sample_main_body.pdf> and <http://www.journals.plos.org/plosone/s/file?id=ba62/PLOSOne_formatting_sample_title_authors_affiliations.pdf> | We have reviewed the style guidelines and confirm they meet the requirements. |
| 3 | Thank you for submitting your clinical trial to PLOS ONE and for providing the name of the registry and the registration number. The information in the registry entry suggests that your trial was registered after patient recruitment began. PLOS ONE strongly encourages authors to register all trials before recruiting the first participant in a study. | Thank you for reviewing this. We apologize for the confusion here. We think this may be because the ‘Last Update’ date was posted June 18, 2019. This update was related to an annual renewal of the study protocol on the ClinicalTrials.gov registry.  The trial, registered at: <https://clinicaltrials.gov/ct2/show/NCT03677193> indicates that this study was first posted September 19, 2018. Further, the initial release date according to the ClinicalTrials.gov Protocol Registration and Results System was dated July 31, 2018. Note, this is before the start of the study enrollment.  Please see the screenshots at the bottom of this document to confirm (Appendix A). If for some reason, the study still appears to be registered after recruitment began, we will indeed contact the registry directly to correct the error. Thank you for bringing this to our attention. The trial registration number may have also been incorrect. We have verified that the proper registration number is in the manuscript. |
| 4 | As per the journal’s editorial policy, please include in the Methods section of your paper: 1) your reasons for your delay in registering this study (after enrolment of participants started); 2) confirmation that all related trials are registered by stating: “The authors confirm that all ongoing and related trials for this drug/intervention are registered”. | Regarding item 1) as described in response #3, above, the study was registered July 31, 2018 on ClinicalTrials.gov. The first participant was enrolled on September 29^th^, 2019.  Regarding item 2) the required statement was added to the section: Methods, Design. |
| 5 | Please also ensure you report the date at which the ethics committee approved the study as well as the complete date range for patient recruitment and follow-up in the Methods section of your manuscript, and clarify why the protocol is dated 23 November 2018. | Methods have been adjusted accordingly and now read as follows:  “Ethical approval was obtained by Holland Bloorview’s Research Ethics Board (approved July 27, 2018, amended November 23, 2018, to include participants with mixed tone and mild dystonia) and the French national Comité de protection des personnes (CPP) (approved July 6, 2018).” – Section: Methods, Design  “Recruitment in Canada took place from September-November 2018. … Recruitment in France took place from November 2018 - January 2019.”- Section: Methods, Participants, Recruitment |
|  |  |  |
|  | **Reviewer #1 comments:** |  |
| 1 | The manuscript describes a technically sound piece of scientific research. The statistic analysis has been conducted rigorously The data support the conclusions and I totally agree with the resource limitation well described at paragraph 430 | Thank you for your kind remarks. |
|  |  |  |
|  | **Reviewer #2** |  |
|  | **General comments** |  |
| 1 | The authors present the results of a study to assess the feasibility of an at-home video game intervention. However, the authors seem to mix a bit the purposes of this study and the paper is difficult to follow. The main aim is to determine the feasibility (adherence, complications) but then the authors also assess the motor functions of the participants before and after the intervention. | We would first like to thank Reviewer 2 for their detailed comments.  Regarding the main aim of this study, we assess feasibility according to recommendations from Thabane et al 2010, and the CONSORT extension for reporting feasibility studies (Eldridge et al 2016). This framework highlights that the aim of feasibility testing can relate to any of four broad classifications: process, resources, management and scientific. Scientific here refers to treatment safety and estimation of treatment effect and its variance. Motor function assessments were completed for this reason. We decided to separate the scientific, effect size and variance, estimate to the secondary aim as we used six outcome measures (since no single measure addresses all aspects of capacity and participation). However, we apologize, as this seems to have caused confusion. To clarify, all the reported outcomes, including those related to motor function are made towards assessing feasibility of the intervention.  It is also worth noting that for this reason, statistical analyses were intentionally not conducted. In fact, the feasibility reporting guide we follow discourages emphasizing statistical significance in studies of this type (Thabane et al 2010, Eldridge et al 2016). The goal here is to inform future randomized controlled trial (RCT) research and not verify the significance of the response, as the study is underpowered to do so.  We have made changes in the main text to clarify our use of the feasibility framework:  “This paper addresses intervention feasibility as articulated by Thabane et al (2010) [18]. This framework highlights that the aim of feasibility testing can be related to one or more of the following four classifications: process, resources, management and scientific. The objective here is to support development towards future randomized controlled trials and not to define statistical or clinical effectiveness of the intervention [18]. In this study, we concentrate on two of the four feasibility classifications:  First, we assess *process feasibility* of the biofeedback-enhanced therapy video game intervention protocol for young people with CP. The objective here is to determine the ability to enroll participants, enable home-based practice, and retain their activity during a 1-month intervention. To this purpose, *a priori* success criteria were established for the recruitment and response rates, adherence, and frequency of technical difficulties impeding home practice [18].  Second, we assess the *scientific feasibility* of the intervention by estimating the effect size and variance for six participant-centred outcome measures for the hand and wrist. The measures are aligned to the Body Functions and Activities and Participation chapters of the ICF (international classification of functioning disability and health) [19,20].” – Section: Introduction, Aim |
| 2 | Then they used unusual methods for presenting the results. The article would become more readable if the authors only present the results of the feasibility part and shorten the presentation of the pre-post results since it’s not the aim of this particular study. | As indicated in the comment above, we assess feasibility according to recommendations from Thabane et al 2010, and the CONSORT extension for reporting feasibility studies (Eldridge et al 2016). This framework highlights that the aim of feasibility testing can relate to any of four broad classifications: process, resources, management and scientific. Scientific here refers to treatment safety and estimation of treatment effect and its variance. Motor function assessments were completed for this reason. All the reported outcomes, including those related to motor function (the pre-post results) are made towards assessing feasibility of the intervention.  To clarify this, we have:   - Added context around feasibility in the aim (please see the quoted text in the comment above). - Re-organized the methods to not distinguish the work in terms of primary and secondary aims, but in reference to *process feasibility* and *scientific feasibility.* You will see this change throughout the aim, methods and results of the manuscript. |
| 3 | A table with the results of the different tests before and after the intervention with results of the statistics would be more suited and easy to interpret, the other analysis can be moved to supplementary materials. | For clarity, we have provided a table of the results of the different tests. Please see Table 3 under the section: Results, Scientific Feasibility.  Please note, as we followed the CONSORT extension for reporting feasibility studies (Eldridge et al 2016) and the Single-Case Reporting Guideline In BEhavioural Interventions ([SCRIBE](https://psycnet.apa.org/fulltext/2016-17384-001.html#appendix_arc_4_1_10)), we have intentionally not reported significance tests. We do however report the combined statistical and visual analysis along with estimates of effect size and their variance in the form of 95% confidence intervals where appropriate.  We hope the reviewer can understand the choice to use this methodology, and not to focus on statistical significance. The goal here is to inform future RCT research and not verify the significance of the response, as the study is underpowered to do so. To this point we have added the following statement:  “At the observed effect size, 0.29, in bimanual performance (AHA change score), 127 participants would be required for the definitive RCT assuming alpha significance set to 0.05 and a desired power = 0.9. AHA change score was chosen to estimate the sample size instead of active wrist extension capacity since it measures functional bimanual performance and has more direct relationship with ability to perform activities of daily living.” – Section: Discussion, Recruitment and adherence |
|  | **Specific comments** |  |
|  | **Introduction** |  |
| 1 | Line 45: The authors should precise the inconsistency in the existing studies and what is currently missing to strengthen their study. | The statement has been changed to:  “These types of studies have shown moderate evidence towards improving balance and overall motor skill but weak evidence towards improving upper extremity skills, joint control, gait and strength [9]” - Introduction. |
| 2 | Line 68: patient instead of client | We have removed the use of the terms: patient and client throughout the manuscript. Our institution’s convention is to use client instead of patient, however we realize this is not true everywhere. To be as inclusive as possible we have used the term participant, person or individual where appropriate. |
|  | **Methods** |  |
| 3 | Intervention: Was the total amount of training (defined during the self-defined practice) significantly different between the participants? The total duration of training should be presented in the results. | Individual practice did vary across participants. While the average amount of training was 16±4 days over the 1-month intervention, 7/19 participants were outside 1 standard deviation from this mean. Similarly, while participants practiced an average of 17±9 minutes/day, 10/19 participants were outside 1 standard deviation from this mean.  Individual total amount of training can now be seen in Table 3 under section: Results, Scientific feasibility. We have also added the following statement:  “System usage varied across individuals but averaged 4±1 days/week (8-24 days total), 17±9 minutes/day (37-333 minutes total), and 163±59 gesture repetitions/day (997-5698 total).” - Section: Results, Participant and recruitment characteristics |
|  | **Results** |  |
| 4 | Table 1: only 2 patients have physiotherapy sessions? It seems low compared to the recommendation. Should be discussed. | We have added the following to the discussion:  “Participants in Canada and France were off treatment blocks but visited their care provider for regularly scheduled check-ups, either annually or quarterly. Two participants, B and F, took additional weekly therapy sessions outside of the rehabilitation centre and one participant, J, attended the school at the rehabilitation centre and would see the occupational therapist as needed.” - Section: Discussion, Implementation  Both Canada and France have publicly funded healthcare but services for individuals in our recruitment demographic (older age group and mild to moderate impairment) are not as frequently utilized. As such, it is not uncommon to see older children and teenagers with CP only occasionally be on active blocks of manual therapy training. |
| 5 | Table 2: 19 patients but for the technical issues 6/39? Please clarify | Technical issues could be reported multiple times during the 1-month intervention. For instance, a participant might report a technical issue once in the first week and twice more in the third week. As such, it is possible to have more technical issues than participants enrolled.  To clarify this metric, we have added the following text to Methods, Outcomes #4:  “Participants may report multiple issues during the 1-month intervention.” |
| 6 | Figure 2 – 3: Forest plots are a very unusual way of presenting individuals' data making the interpretation confusing! How did you compute CI for the SMD based only on 1 individual data? What’s the point of using a random effect model since it’s a controlled study? Participants should all have the same weight. A simple t-test (or Wilcoxon if the data are not normally distributed, which is not precised) is more adapted (eventually with line plots if you want to present individual’s results). | Thank you for pointing this out. As described in the response to this reviewer’s general comments numbers 1 and 3, we are not presenting statistical tests such as t-tests or Wilcoxon to conform with the CONSORT feasibility testing and SCRIBE Single-Case design reporting frameworks.  We do however acknowledge that this is an unusual presentation of individual results. Given the individual outcomes are now reported in Table 3, we have removed Fig 2 and Fig 3 and reported group level SMD and confidence intervals descriptively (Section: Methods, Scientific feasibility). |
| 7 | Figure 4: again visual analyses, the authors state that there did not compute the significance but still present a lot a statistics, this is a bit confusing. | As described in the response to this reviewer’s general comments numbers 1 and 3, significance tests comparing pre and post outcomes were not conducted in accordance with the CONSORT feasibility testing and SCRIBE Single-Case design reporting frameworks.  However, statistical analyses were conducted to establish effect sizes and variances as recommended by CONSORT feasibility testing and SCRIBE Single-Case design reporting. SCRIBE recommends the presentation of a mixed ‘visual and statistical’ approach (Manolov et al 2014). The methods are considered complementary rather than mutually exclusive. We acknowledge that there is no universal gold-standard for SCED design studies, and it is for this reason we provide a mixed analysis approach. |
| 8 | Was there any difference between the experiments in France and in Canada? | No, the experiments in France and Canada were completed the same way. The same researcher trained the participants, completed all in-home assessments and measures of range of motion and grip strength. The only difference was in recruitment where:  “In Canada, eligible participants were also identified through the hospital’s centralized recruitment database, connect2research. In Canada the researcher telephoned potential participants after sending an invitation letter by mail. Then, the researcher screened interested participants and obtained written informed consent. In France, the developmental pediatricians invited eligible individuals to participate. They obtained written informed consent from interested participants.”  This is currently expressed in the section: Methods, Participants, Recruitment. |
|  | **Discussion** |  |
| 9 | Body function: Against it is a bit confusing to read “In the current study, all but two participants increased active wrist extension” since no proper statistics were done. | This statement has been further specified for clarity and now reads:  “In the current study, 12 of 17 participants increased active wrist extension by at least 5 degrees.” – Section: Discussion, Body Function  Additionally, we have added the following to the methods:  “values are recorded to the nearest five degrees, the minimal detectable difference [42].” – Section: Methods, Outcomes |
| 10 | “Accordingly, we saw 18% average improvement with +5% gains in 12/19 participants.” Idem, what about the 7 other participants? | 5 participants had a grip strength change of <5%, 2 participants did not complete enough assessments to calculate grip strength or range of motion during the intervention (see Table 3 under the section: Results, Scientific feasibility).  To provide additional context, the discussion has been changed to the following:  “Accordingly, we saw 18% average improvement in non-dominant grip strength relative to the dominant side across participants. However, normative data for children’s grip strength, as compared to the dominant side using the modified sphygmomanometer are not available”. – Section: Discussion, Body Function  further, we have added the following limitation:  “Next, level-change groupings (small, moderate, and large) for active wrist extension were based on a minimal detectible difference of five degrees [42]. However, similar level-change groupings in grip strength were undeterminable since minimal detectible differences for grip strength, as compared to the dominant hand, do not yet exist for young people with CP. Scores are reported as a percent of the dominant side since improved capacity for bimanual activities is a primary goal for many young people with CP. For context, raw score increases in the affected hand's grip strength averaged 17 mmHg and all but two participants saw an improvement of greater than 10 mmHg. A minimal detectible difference of seven mmHg and a within-subject Standard Error of Measurement of three mmHg has recently been reported in individuals with Parkinson's Disease using a similar modified sphygmomanometer test [71]. These visual analyses summaries are not provided as a definitive evaluation but to aid the reader in their interpretation of the effect size in this single-case design intervention [23].”– Section: Discussion, Limitations |
| 11 | Since it is a feasibility study the author should discuss the feasibility of implementing such kind of interventions in “real life” conditions. Do they think it is possible to have the pre-session and the different control sessions organized in practice (and who is going to pay for that). This is particularly important since apparently only 2 of the 19 participants have physiotherapy sessions (so basically no intervention). | The reviewer raises an important concern. We have added the following text to address the reviewers question surrounding ‘real life’ implementation:  “It is important to note that this is the first investigation of the novel home-based gaming intervention and accordingly focused on *process* and *scientific feasibility*. Specifically, the study focuses on determining the extent to which the intervention could be used at home and estimating the effect size and variance it might have. Towards conducting a complete RCT, more studies and development would be needed to ascertain real-world feasibility, addressing resources and management. This includes a proper health economics analysis and evaluation of the logistic organization with respect to an institute’s existing therapeutic practices.” – Discussion, Implementation  Main points related to process feasibility are in the discussion under the sections: Recruitment and adherence, Motivation and Limitations. To further comment on *scientific feasibility*, as described by Thabane et al 2010, we discuss Body Functions and Activity and Participation outcomes in the discussion as well. |

**References**

Thabane L, Ma J, Chu R, Cheng J, Ismaila A, Rios LP, et al. A tutorial on pilot studies: the what, why and how. BMC Med Res Methodol. 2010;10: 1.

Eldridge SM, Chan CL, Campbell MJ, Bond CM, Hopewell S, Thabane L, et al. CONSORT 2010 statement: extension to randomised pilot and feasibility trials. Pilot Feasibility Stud. 2016;2: 64. doi:10.1186/s40814-016-0105-8

Manolov R, Gast DL, Perdices M, Evans JJ. Single-case experimental designs: Reflections on conduct and analysis. Neuropsychol Rehabil. 2014;24: 634–660. doi:10.1080/09602011.2014.903199

**Appendix A: Screenshots of ClinicalTrials.gov showing trial registration verification date before recruitment began.**

Screenshot from ClinicalTrials.gov PRS showing ‘Record Verification’ date: July 2018.
