# Supplementary material for: A biofeedback-enhanced therapeutic exercise video game intervention for young people with cerebral palsy: A randomized single-case experimental design feasibility study
Source: PLoS One. 2020 Jun 22;15(6):e0234767. doi: 10.1371/journal.pone.0234767 (PMC7307764; doi:10.1371/journal.pone.0234767)
Supplement: Attachment — Submitted filename: Response to Reviewers.docx [file pone.0234767.s011.docx]

**Response to Reviewers - PONE-D-19-22575**

| **#** | **Review** | **Response** |
| --- | --- | --- |
|  | **Reviewer 3** |  |
| 1 | This is a very nicely done, and well-reported study, on an important topic. I only have a few comments; overall, I enjoyed reading the paper and commend the authors on their clear presentation of the results. | Thank you for your comments we appreciate your suggestions and the time spent reviewing. |
| 2 | l.72 "participants'" not "participant's" The plural is implied later in the sentence with pronoun "their." Throughout the paper, the authors should check pronoun agreement between subject and verb. In a number of places, a singular subject is followed by a plural pronoun; e.g., he...their (sorry, ex-grammar teacher). | Thank you for this comment since English language grammar is not a particular strength of our group. The manuscript has been reviewed for pronoun agreement and the corrections have been made as needed, including editing l.72 from “participant’s” to “participants”. |
| 3 | l. 145. Why is "Normal or corrected-to-normal vision and hearing" an EXCLUSION criteria? Is that correct? | This is indeed a typo. That item has been moved from the exclusions to the inclusions as it should be. Thank you for noticing the mistake. |
| 4 | In several places, the authors give an abbreviation, followed by the full name that is being abbreviated in parentheses (ll.100-01, ll.164-65, ll.170-71). This order should be reversed (as it is elsewhere in the paper). | The location of abbreviations have been corrected to appear in brackets after the full name at each of the lines identified by the reviewer. |
| 5 | Table 3 summarizes Reps, Minutes Active, and Minute in System using means and standard deviations. Judging by the size of the standard deviations relative to the means, these do not appear to be normally distributed, and would be better summarized with medians and interquartile ranges to give a more accurate picture of the data distributions. | Table 3 has been updated to include median and interquartile range as the reviewer suggests giving a more accurate picture of the data distributions. |
| 6 | 25% of the participants did not reach their goals due to a loss of interest. This seems fairly high. Did the authors factor this into their assessment of feasibility? I know they talk about the need to vary programs for different ages and over time to avoid this, but is THAT feasible? I.e., how much money and time go into designing and implementing a single game? | The reviewer addresses an important aspect here. Adherence is a central issue. In the current study we found that adherence was relatively high compared to other studies which have shown participants complete 53-78% of practice goals, as compared to 75% in the current work. (l.381-383). From a feasibility perspective, the reviewer also raises a salient issue. The cost of activity development relative to the expected use and benefit will dictate quantity and quality. We mitigated some of this cost and improved feasibility by finding a suitable game and subsequently collaborating with directly with the developer. We have expanded the limitations section to include this point and it now read as follows:  “…greater choice and game variety, while challenging to implement in rehabilitation protocols, would help maintain novelty and interest in the activity. Collaboration with independent game developers can improve feasibility by offering content with relatively quick and flexible modification abilities, as was the case in the current study with the adapted commercial video game (Dashy Square). [71]” – l.472. |
